# Supplementary material for: Self-assembly of an anion receptor with metal-dependent kinase inhibition and potent in vitro anti-cancer properties
Source: Nat Commun. 2021 Jun 23;12:3898. doi: 10.1038/s41467-021-23983-3 (PMC8222254; doi:10.1038/s41467-021-23983-3)
Supplement: Supplementary file 1 — Supplementary Information [file 41467_2021_23983_MOESM1_ESM.pdf]

Self-Assembly of an Anion Receptor with Metal-dependent Kinase Inhibition and Potent *In Vitro* Anti-Cancer Properties.

Simon J. Allison,[1]* Christopher J. Clemett,[1] Jaroslaw Bryk,[1] Robert A. Faulkner,[1] Michael Ginger,[1] Hollie B. S. Griffiths,[1] Jane Harmer,[1] P. Jane Owen-Lynch,[1] Emma Pinder,[1] Heiko Wurdak,[2] Roger M. Phillips[1]* and Craig R. Rice.[1]*

[1]School of Applied Sciences, University of Huddersfield, Queensgate, Huddersfield, HD1 3DH, UK

[2]School of Medicine, University of Leeds, Leeds, LS2 9JT, UK

Electronic Supplementary Information

A. Chemical Synthesis and Analysis.

Unless otherwise stated, all solvents and materials were purchased from either Sigma Aldrich, Fisher Scientific or Fluorochem and were used without further purification. ^{1}H, ^{13}C, DEPT-135 and DEPT-90 NMR data was recorded on either a Bruker Fourier 300 MHz or Bruker Avance III (AVIII) 400 MHz spectrometer or a Bruker Avance Neo 600 MHz NMR spectrometer. Mass spectra were obtained on an Agilent 6210 TOF MS with electrospray ionisation operating in positive ion mode and mass spectra of metal complexes were obtained on a Bruker Micro TOF-q LC mass spectrometer with electrospray ionisation operating in positive ion mode. Phosphorylated 'SAMS' peptide HMRSAMS*GLHLVKRR (*phosphorylated on the serine residue) was obtained from PeptideSynthetics, Peptide Protein Research Ltd (>95% purity).

1. Ligand synthesis.

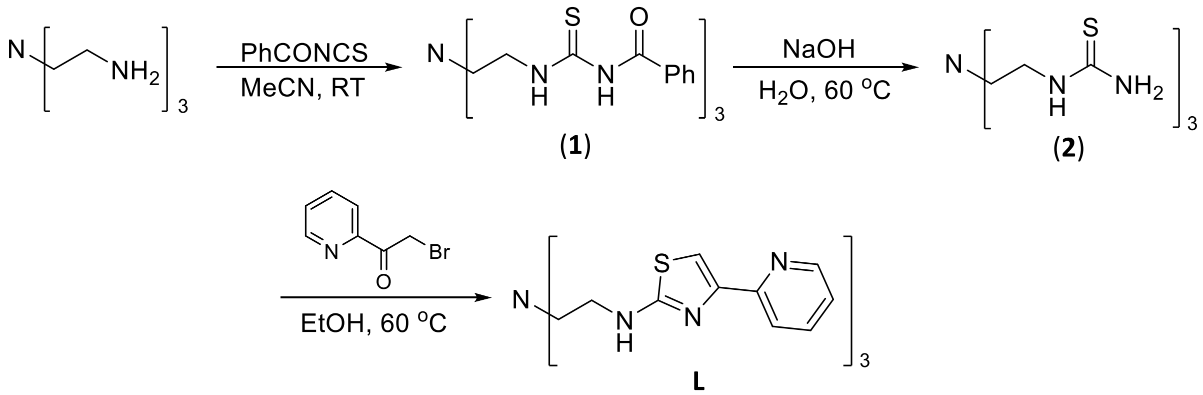

Supplementary Figure 1.1 Synthesis of Ligand **L**

The ligand was prepared as described previously.[1] However, procedures for the synthesis of the benzoylated thioamide (**1**) and the thioamide (**2**) were slightly modified.

Synthesis of (**1**).

To a solution of tris(2-aminoethyl)amine (1.0 g, 6.84 mmol) in acetone (50 mL) under an atmosphere of dinitrogen was added benzoyl isothiocyanate (3.7 g, 22.6 mmol) at such a rate to cause the reaction to gently reflux. After addition the reaction was stirred overnight during which time a colourless

precipitate formed. The precipitate was isolated by filtration and washed with acetone (3 × 5 mL) giving (**1**) as a white solid. Yield = 2.05 g (47 %). ^1H NMR (400 MHz, DMSO-*d*^{6}) δ (ppm) 11.20 (s, 3H, -NH), 11.0 (t, 3H, *J* = 4.8, -CH_2NH), 7.81 (d, 6H, *J* = 7.2, Ph), 7.56 (t, 3H, *J* = 7.4, Ph), 7.38 (t, 6H, *J* = 7.6, Ph), 3.75 (q, 6H, *J* = 5.7, -CH_2CH_2NH), 2.89 (t, 6H, *J* = 6.0 Hz, -CH_2CH_2NH). ^{13}C NMR [100 MHz, DMSO-*d*^{6}]: δ_C = 180.5 (C=S), 168.2 (C=O), 133.1 (CH), 132.7 (Q), 128.9 (CH), 128.6 (CH), 51.9 (CH_2), 42.6 (CH_2). ESI-MS *m/z* 636 (M + H^+), HR ESI-MS found 636.1882 $C_{30}H_{33}N_7S_3O_3$ requires 636.1880 (error 0.46 ppm).

Synthesis of (**2**)

The benzoylated urea derivative (**1**) (1.3 g, 2.05 mmol) was suspended in water (20 mL) and NaOH (820 mg, 20.5 mmol) added. The reaction was then heated to 60°C and after 48 hrs the temperature was incrementally decreased allowing the solution to slowly cool to room temperature, avoiding formation on an oil and resulting in the formation of a colourless precipitate. Isolation by filtration and washing with ice cold water (2 x 1 mL) gave the tri-thiourea (**2**) as a colourless solid. Yield = 503 mg (76 %). ^1H NMR (400 MHz, DMSO-*d*^{6}) δ (ppm) 7.55 (brs, 3H, -NH), 7.08 (brs, 6H, -NH_2), 3.44 (brs, 6H, -CH_2CH_2NH), 2.58 (brs, 6H, -CH_2CH_2NH). ^{13}C NMR [100 MHz, DMSO-*d*^{6}]: δ_C = 183.5 (C=S), 52.9 (CH_2), 42.3 (CH_2). ESI-MS *m/z* 324 (M + H^+), HR ESI-MS found 324.1089 $C_9H_{21}N_7S_3$ requires 324.1093 (error 1.03 ppm).

Synthesis of complexes.

Synthesis of [**L**Zn]$(ClO_4)_2$. To a solution of $Zn(ClO_4)_2{\cdot}6H_2O$ (10 mg. 0.027 mmol) in Me_2CO (1 ml) was added a suspension of ligand **L** (12 mg, 0.019 mmol) in Me_2CO and the reaction sonicated until a clear solution had formed. To this was added water (~1 ml) and the solution slowly allowed to evaporate, during which time pale yellow crystals were formed which were isolated by filtration and dried (yield = 9 mg. 53%*). The [**L**Mn]$(ClO_4)_2$ complex was prepared in an analogous fashion using $Mn(ClO_4)_2{\cdot}6H_2O$ and MeCN giving yellow crystals (yield = 10 mg, 60%*). *percentage yield based on the moles of ligand used. C, 39.03; H, 3.44; N, 15.04%; $C_{30}H_{30}N_{10}S_3ZnCl_2O_8{\cdot}2H_2O$ requires C, 38.87; H, 3.7; N, 15.11%.

Synthesis of [$\mathbf{L}_2Zn_3(SO_4)$]$(ClO_4)_4$. To a solution of $Zn(ClO_4)_2{\cdot}6H_2O$ (10 mg. 0.027 mmol) in MeCN (1 ml) was added a suspension of ligand **L** (12 mg, 0.019 mmol) in MeCN and the reaction sonicated until a clear solution had formed. To this was added water (~1 ml) containing Bu_4NHSO_4 (3.1 mg, 0.009 mmol) and the solution slowly allowed to evaporate during which time colourless crystals were formed which were isolated by filtration and dried (yield = 11 mg, 60%). The [$\mathbf{L}_2Mn_3(SO_4)$]$(ClO_4)_4$ complex was prepared in an analogous fashion using $Mn(ClO_4)_2{\cdot}6H_2O$ giving yellow crystals (yield = 9 mg, 50%). ^1H NMR (400 MHz, 10% CD_3OD in CD_3CN): δ_H (ppm) = 8.20 (m, 12H, py), 7.99 (dt, 6H, *J* = 4.24, 1.1 Hz, py), 7.59 (s, 6H, thiazole), 7.58 (m, 6H, py) 3.97 (m, 12H, -CH_2-), 3.66 (m, 12H, -CH_2-). ^{13}C NMR [100 MHz, 10% CD_3OD in CD_3CN]: δ_C = 172.5, 148.6, 147.9, 143.9, 142.5, 126.3, 123.3, 109.6, 58.9, 55.0. Found: C, 36.33; H, 3.28; N, 14.02%; $C_{60}H_{60}N_{20}S_7Zn_3O_{20}Cl_4{\cdot}3H_2O$ requires C, 36.08; H, 3.33; N, 14.02%.

Synthesis of [$\mathbf{L}_2Cu_3(O_3POPh)$]$(ClO_4)_4$. To a solution of $Cu(ClO_4)_2{\cdot}6H_2O$ (10 mg. 0.027 mmol) in MeCN (1 ml) was added a suspension of ligand **L** (12 mg, 0.019 mmol) in MeCN and the reaction sonicated until a clear solution had formed. To this was added water (~1 ml) containing Na_2O_3POPh (2.3 mg, 0.009 mmol) and the solution slowly allowed to evaporate giving green crystals which were isolated by filtration and dried (yield = 9 mg, 47 %).

Synthesis of [$\mathbf{L}_2Zn_3(PO_4)$]$(ClO_4)_3$. To a solution of $Zn(ClO_4)_2{\cdot}6H_2O$ (10 mg. 0.027 mmol) in MeCN (1 ml) was added a suspension of ligand **L** (12 mg, 0.019 mmol) in acetone and the reaction sonicated until a clear solution had formed. To this was added water (~1 ml) containing Na_2O_3POPh (2.3 mg,

0.009 mmol) and the solution slowly allowed to evaporate giving yellow crystals which were isolated by filtration and dried (yield = 10 mg, 54 %). The [**L**$_2$Mn$_3$(PO$_4$)](ClO$_4$)$_3$ complex was prepared in an analogous fashion using Mn(ClO$_4$)$_2$·6H$_2$O and a few drops of MeOH to aid dissolution, giving yellow crystals (yield = 9 mg, 50%). ^{1}H NMR (400 MHz, 10% CD$_3$OD in CD$_3$CN): δ_H (ppm) = 8.50 (m, 12H, py), 7.89 (d, 6H, *J* = 5.2, py), 7.44 (sextet, 6H, *J* = 7.4, py), 7.33 (s, 6H, thiazole) 3.97 (t, 12H, *J* = 5.6, -CH$_2$-), 3.76 (t, 12H, *J* = 5.7 Hz, -CH$_2$-). ^{13}C NMR [100 MHz, 10% CD$_3$OD in CD$_3$CN]: δ_C = 173.2, 149.3, 147.7, 144.7, 141.5, 125.8, 122.6, 107.5, 59.1, 56.5. [L$_2$Zn$_3$(PO$_4$)](BF$_4$)$_3$. Found: C, 40.86; H, 3.6; N, 15.13%; $C_{60}H_{60}N_{20}S_6Zn_3PO_4B_3F_{12}\cdot C_3H_6O$ requires C, 40.61; H, 3.57; N, 15.04%.

[L$_2$Cu$_3$(SO$_4$)](ClO$_4$)$_4$. Yield = 76%. Found: C, 36.19; H, 3.03; N, 13.89%; $C_{60}H_{60}N_{20}S_7Cu_3Cl_4O_{20}\cdot 2H_2O$ requires C, 36.5; H, 3.27; N, 14.19%.

[L$_2$Cu$_3$(PO$_4$)](ClO$_4$)$_3$. Yield = 99%. Found: C, 38.47; H, 3.24; N, 14.91%; $C_{60}H_{60}N_{20}S_6Cu_3PCl_3O_{16}\cdot H_2O$ requires C, 38.84; H, 3.37; N, 15.10%.

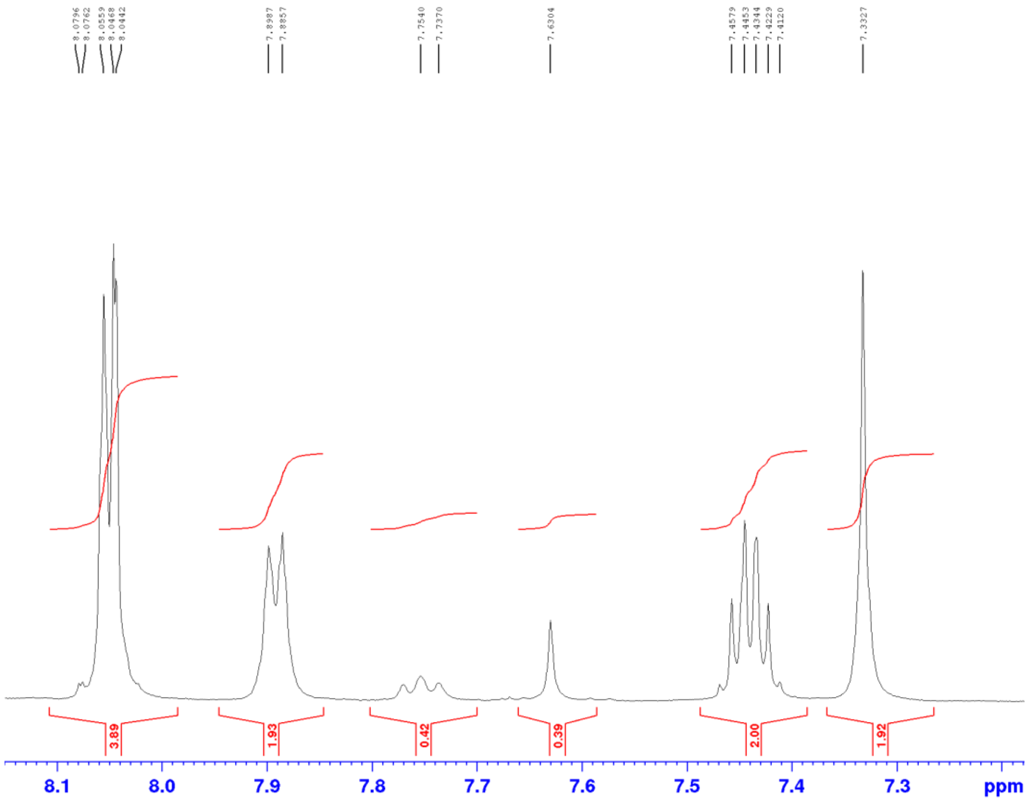

Supplementary Figure 1.2 Aromatic region of the ^{1}H NMR (10% CD$_3$OD in CD$_3$CN) spectra of the aromatic region for [**L**$_2$Zn$_3$(PO$_4$)]$^{3+}$.

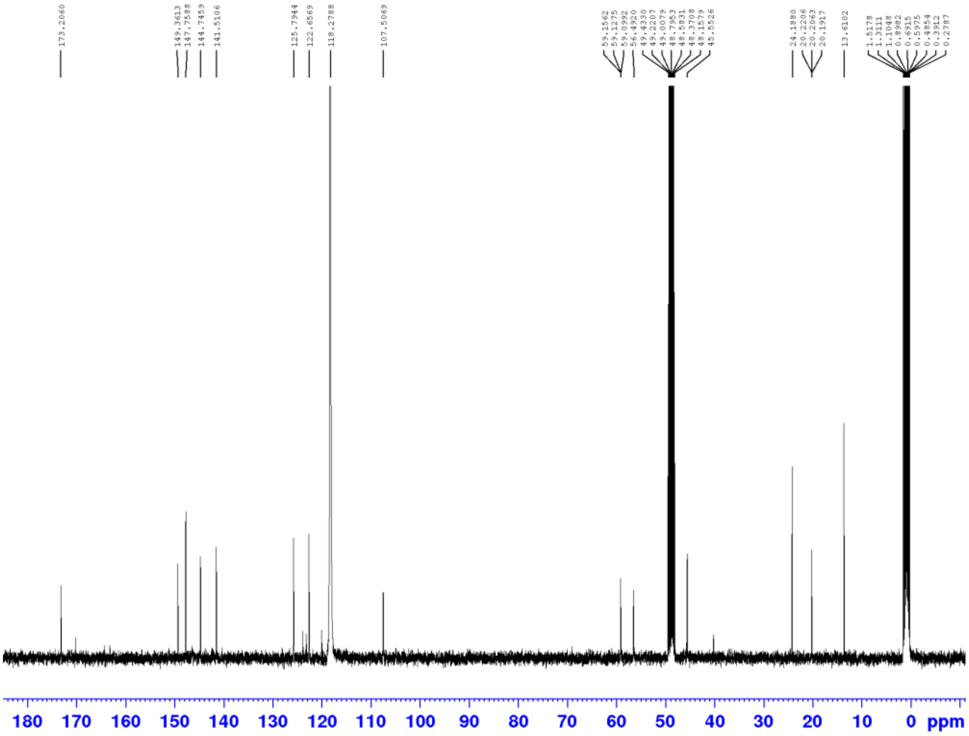

Supplementary Figure 1.3 ^{13}C NMR (10% CD_3OD in CD_3CN) spectra of $[\mathbf{L}_2Zn_3(PO_4)]^{3+}$. Signals 45 – 10 ppm correspond to Bu_4N^+ used in the synthesis. Signals at 0.6 and 118.3 ppm arise from CD_3CN and 49.0 ppm from CD_3OD.

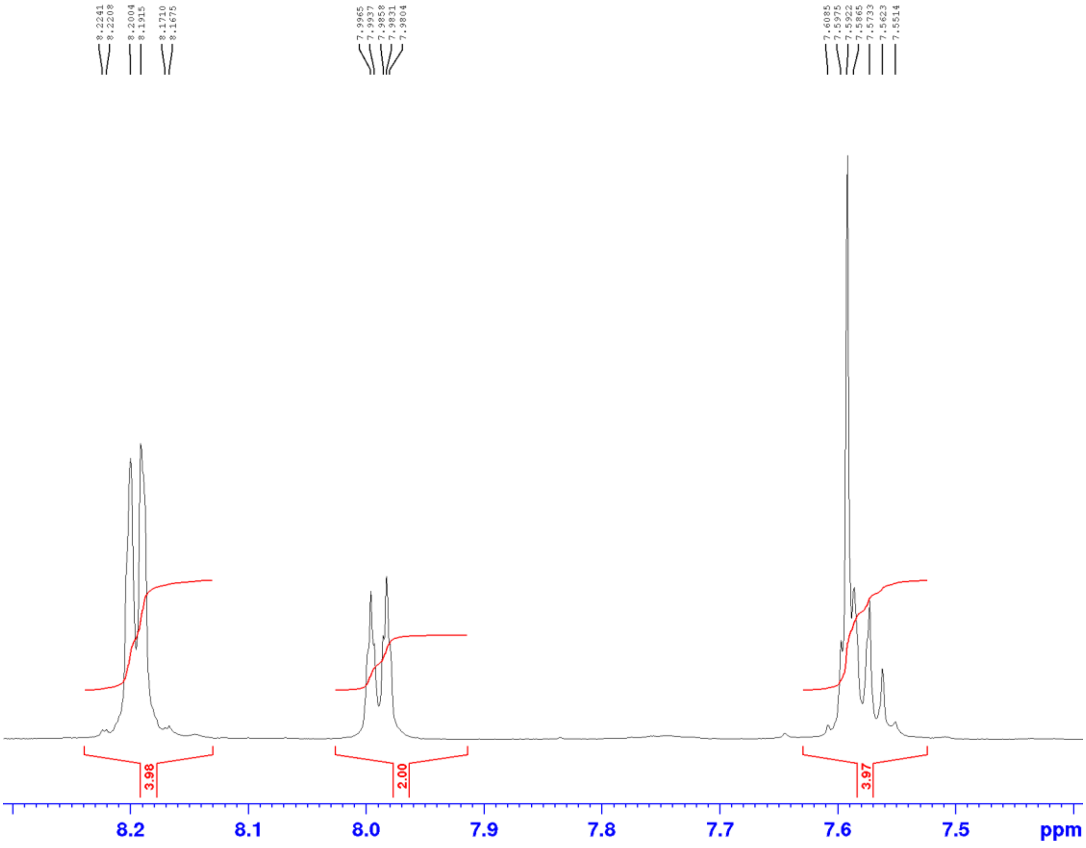

Supplementary Figure 1.4 Aromatic region of the ^{1}H NMR (10% CD_3OD in CD_3CN) spectra of the aromatic region for $[\mathbf{L}_2Zn_3(SO_4)]^{4+}$.

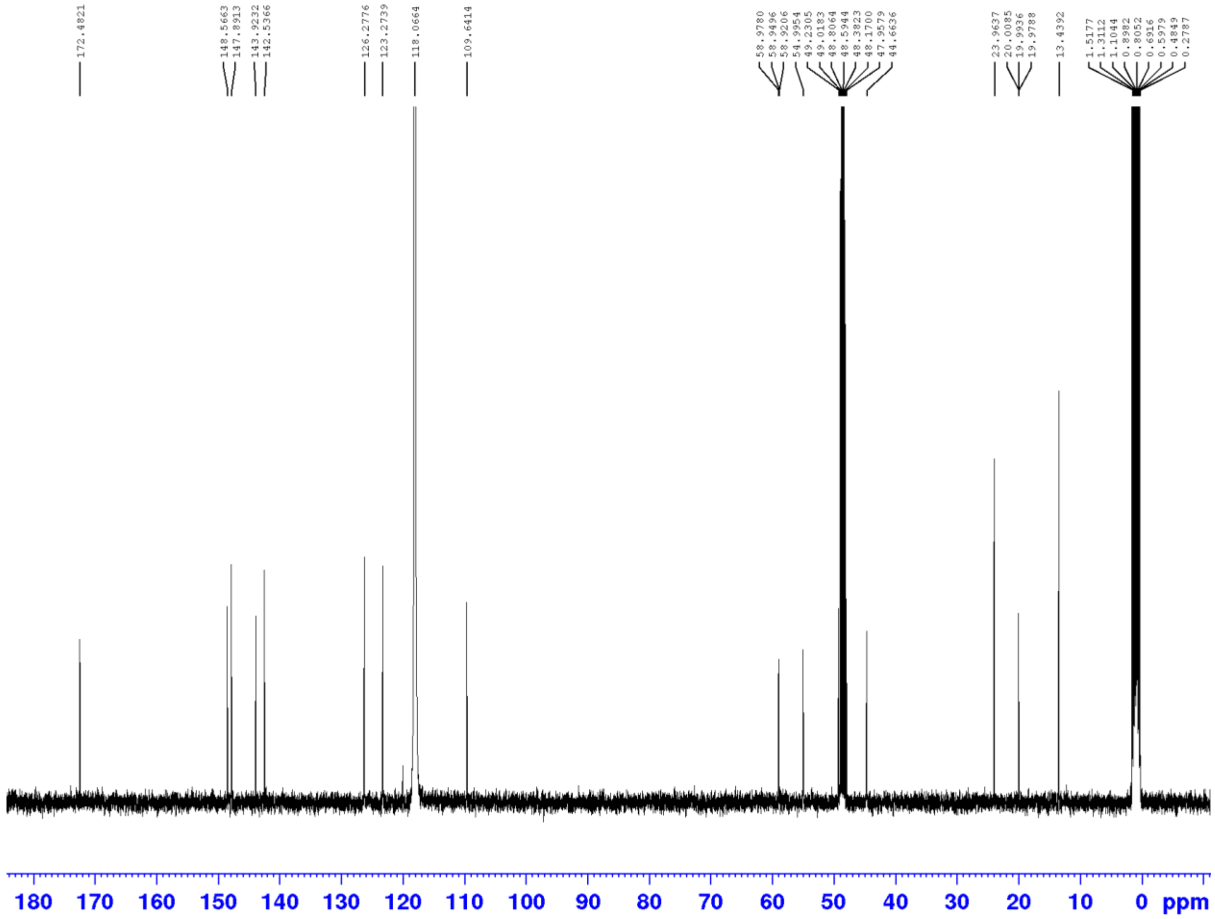

Supplementary Figure 1.5 ^{13}C NMR (10% CD_3OD in CD_3CN) spectra for [$\mathbf{L}_2Zn_3(SO_4)$]$^{4+}$. Signals 45 – 10 ppm correspond to Bu_4N^+ used in the synthesis. Signals at 0.6 and 118.3 ppm arise from CD_3CN and 49.0 ppm from CD_3OD.

2. Mass spectrometry studies.

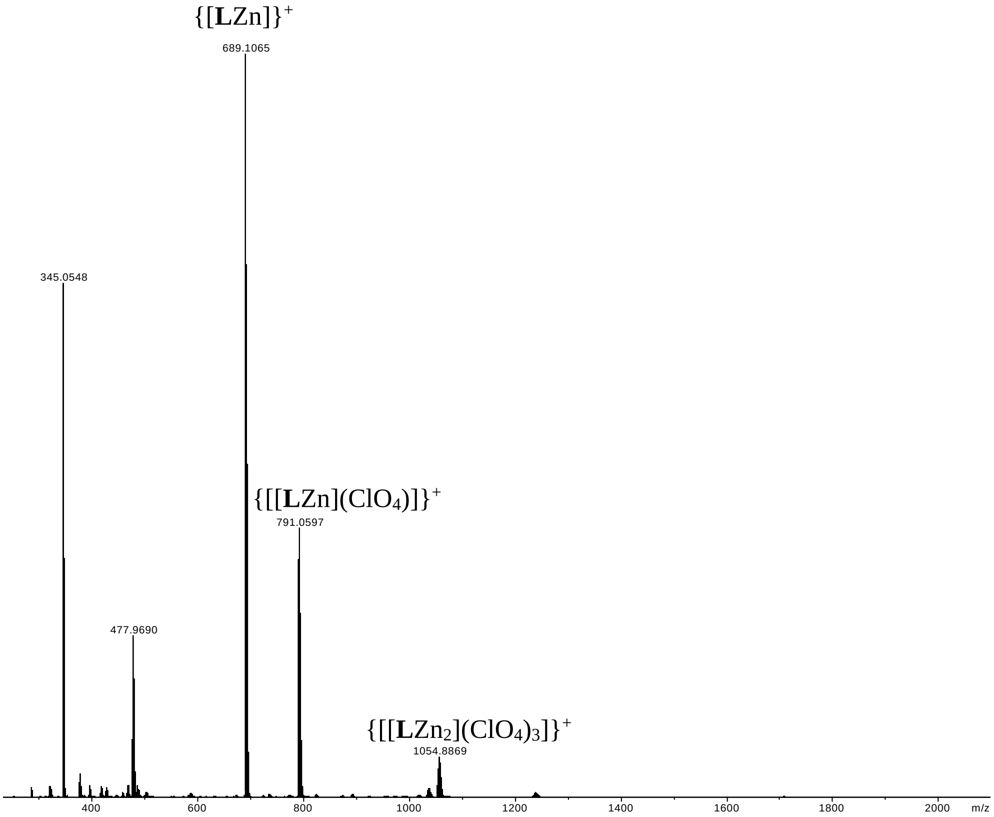

Supplementary Figure 2.1. ESI-MS of [**L**Zn]$(ClO_4)_2$.

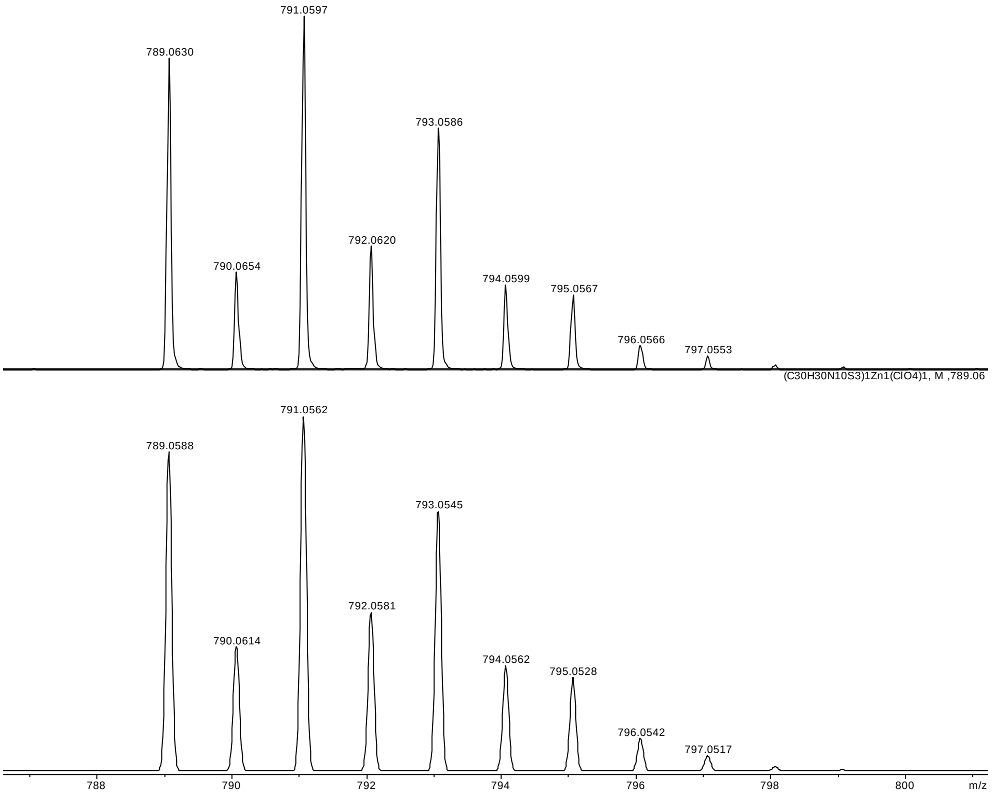

Supplementary Figure 2.2: ESI-MS isotope pattern for *m/z* 789 {[[**L**Zn](ClO_4)]}$^+$ (Top: actual signal, bottom: simulated pattern).

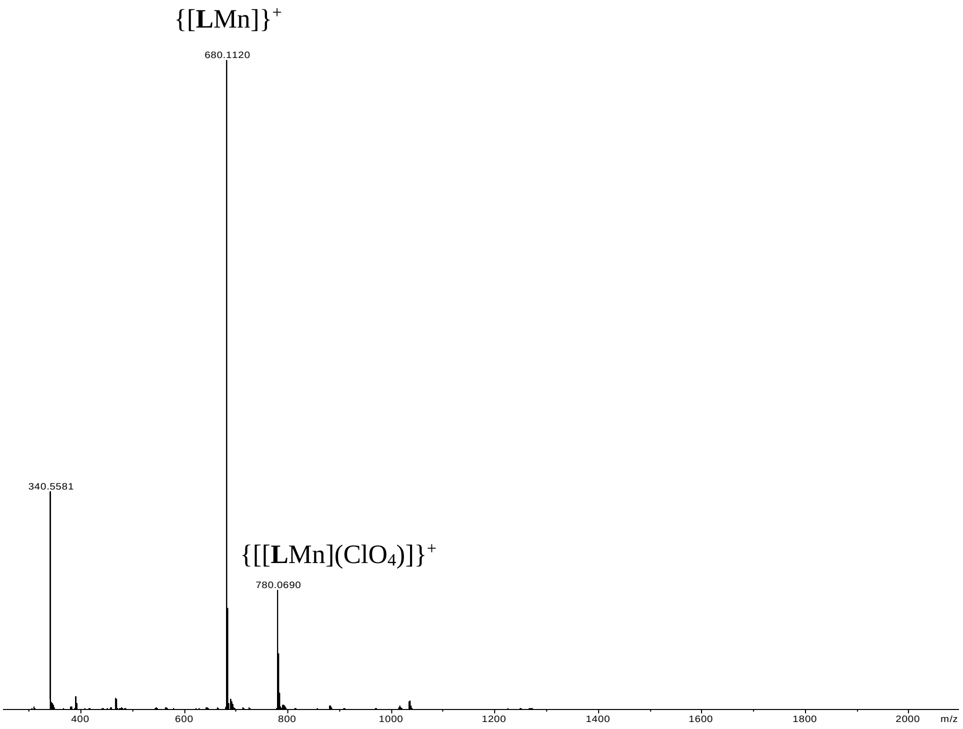

Supplementary Figure 2.3. ESI-MS of [**L**Mn](ClO_4)$_2$.

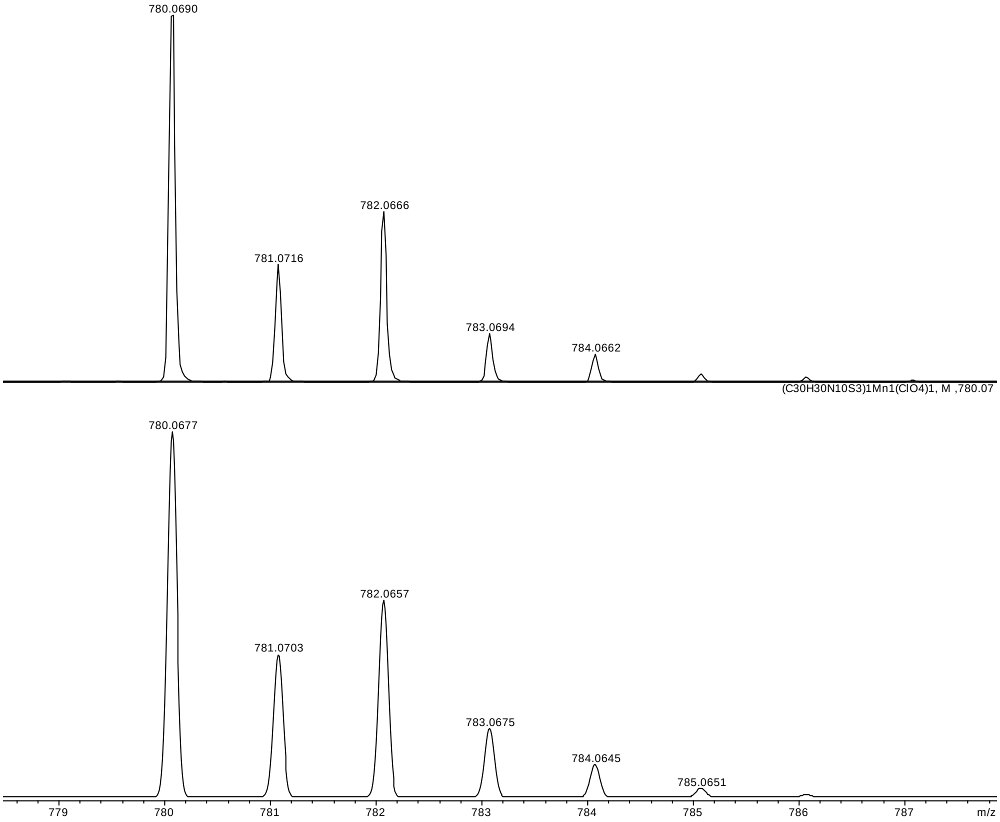

Supplementary Figure 2.4: ESI-MS isotope pattern for *m/z* 780 {[[**L**Mn](ClO_4)]}$^+$ (Top: actual signal, bottom: simulated pattern).

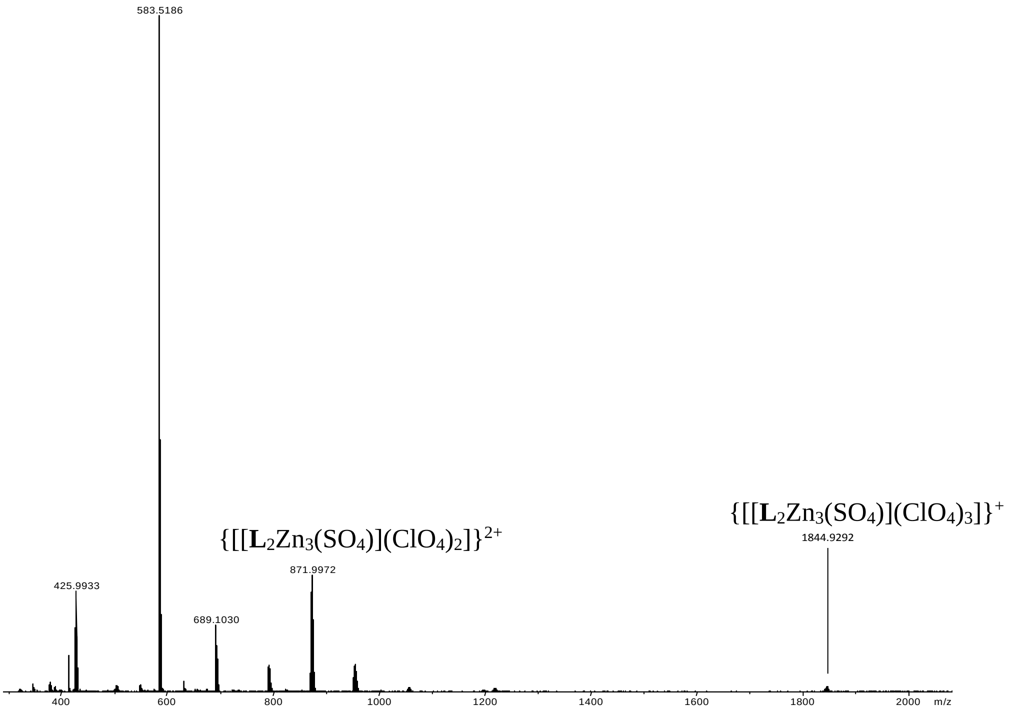

Supplementary Figure 2.5. ESI-MS of [$\mathbf{L}_2Zn_3(SO_4)$]$(ClO_4)_4$.

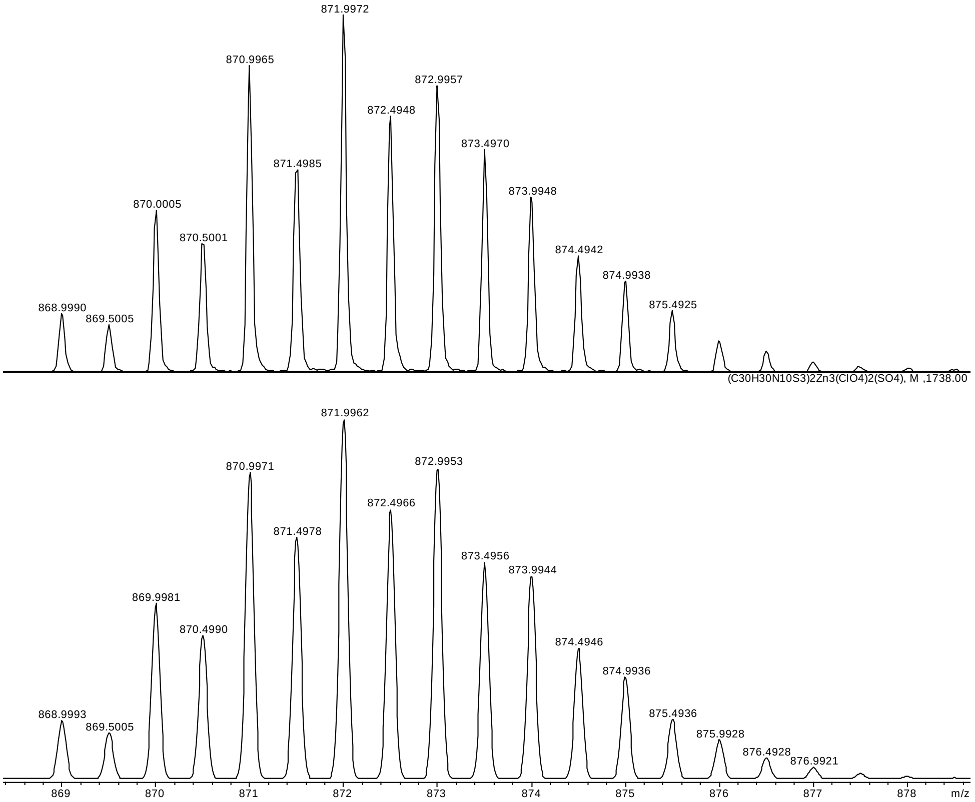

Supplementary Figure 2.6: ESI-MS isotope pattern for *m/z* 1738 {[[$\mathbf{L}_2Zn_3(SO_4)$]$(ClO_4)_2$]}$^{2+}$ (Top: actual signal, bottom: simulated pattern).

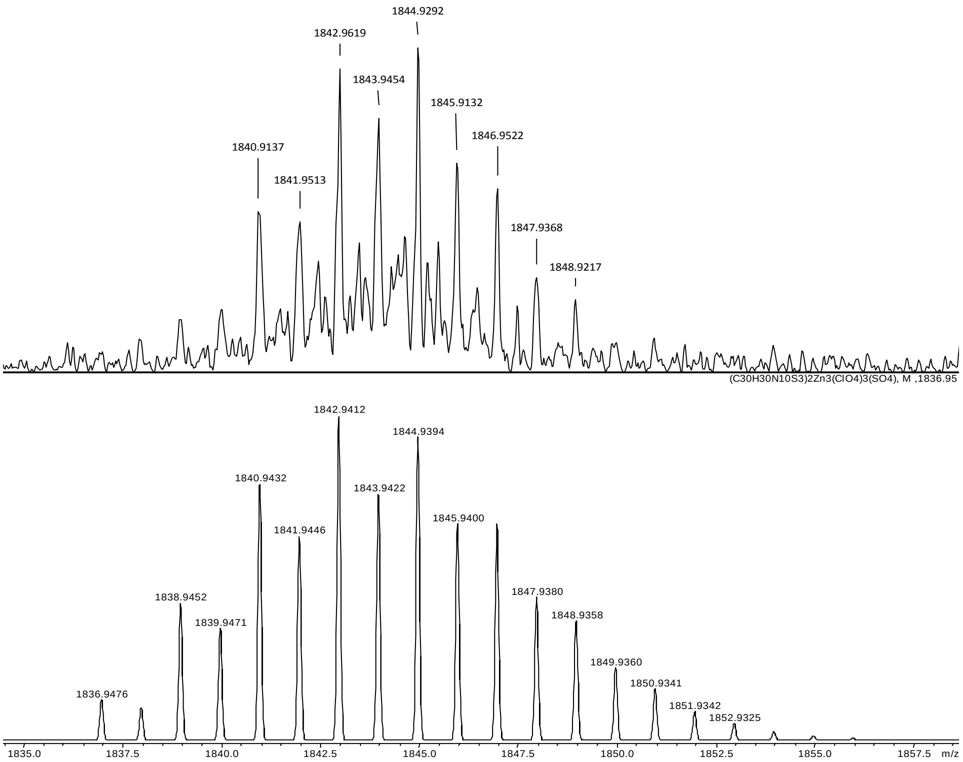

Supplementary Figure 2.7: ESI-MS isotope pattern for *m/z* 1837 {[[**L**$_2$Zn$_3$(SO$_4$)](ClO$_4$)$_3$]}$^+$ (Top: actual signal, bottom: simulated pattern).

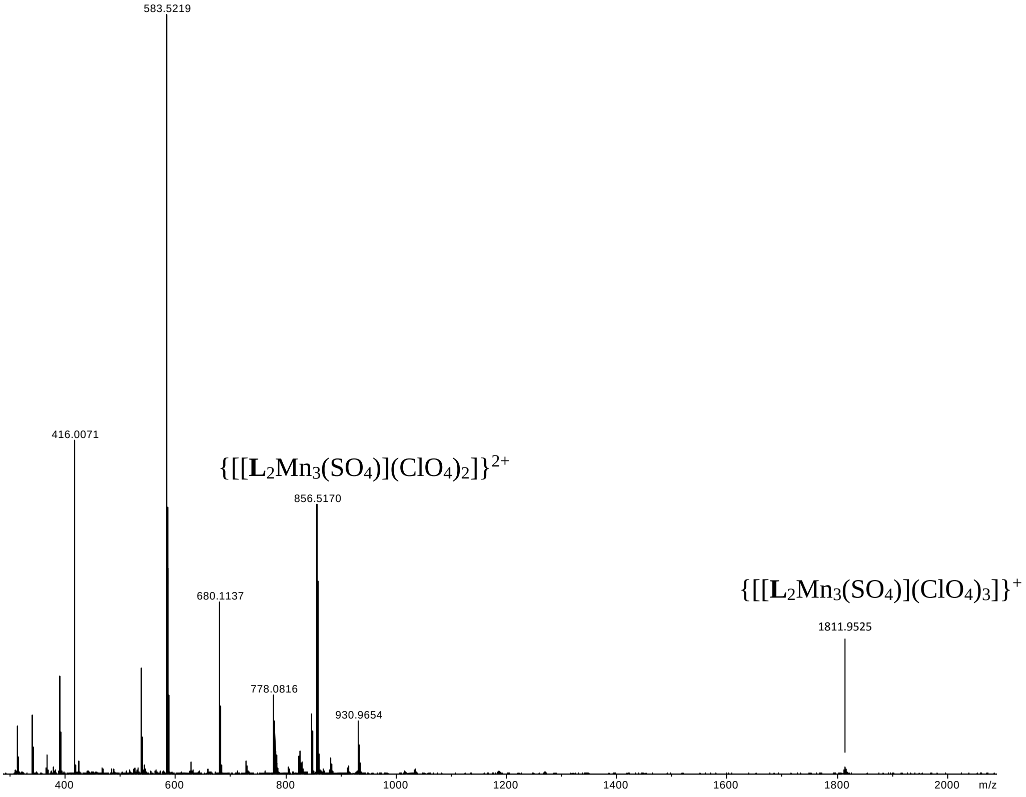

Supplementary Figure 2.8. ESI-MS of [**L**$_2$Mn$_3$(SO$_4$)](ClO$_4$)$_4$.

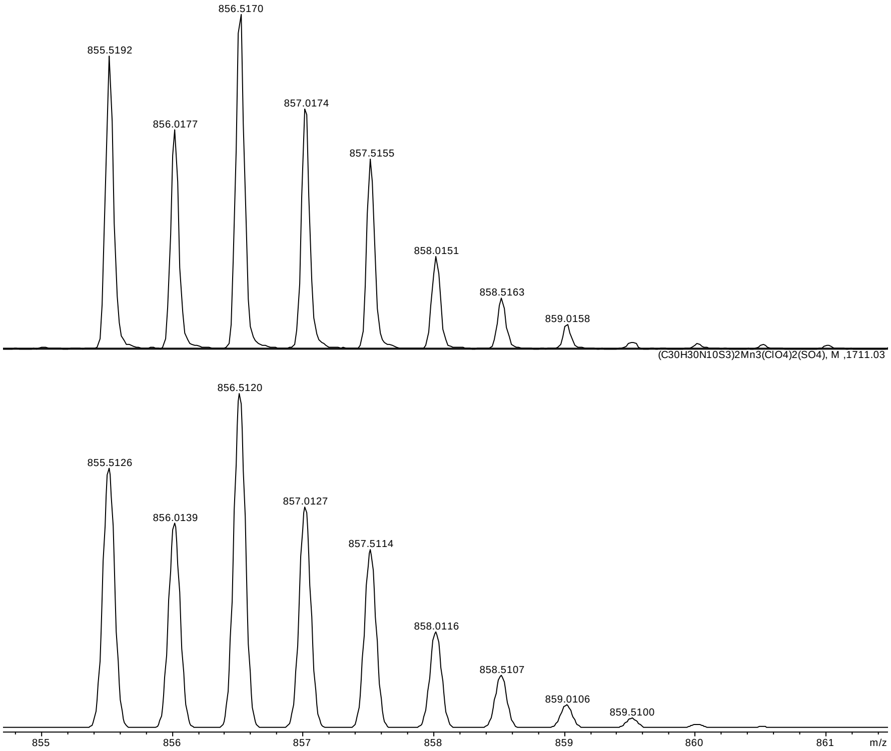

Supplementary Figure 2.9: ESI-MS isotope pattern for *m/z* 1711 {[[**L**$_2$Mn$_3$(SO$_4$)](ClO$_4$)$_2$]}$^{2+}$ (Top: actual signal, bottom: simulated pattern).

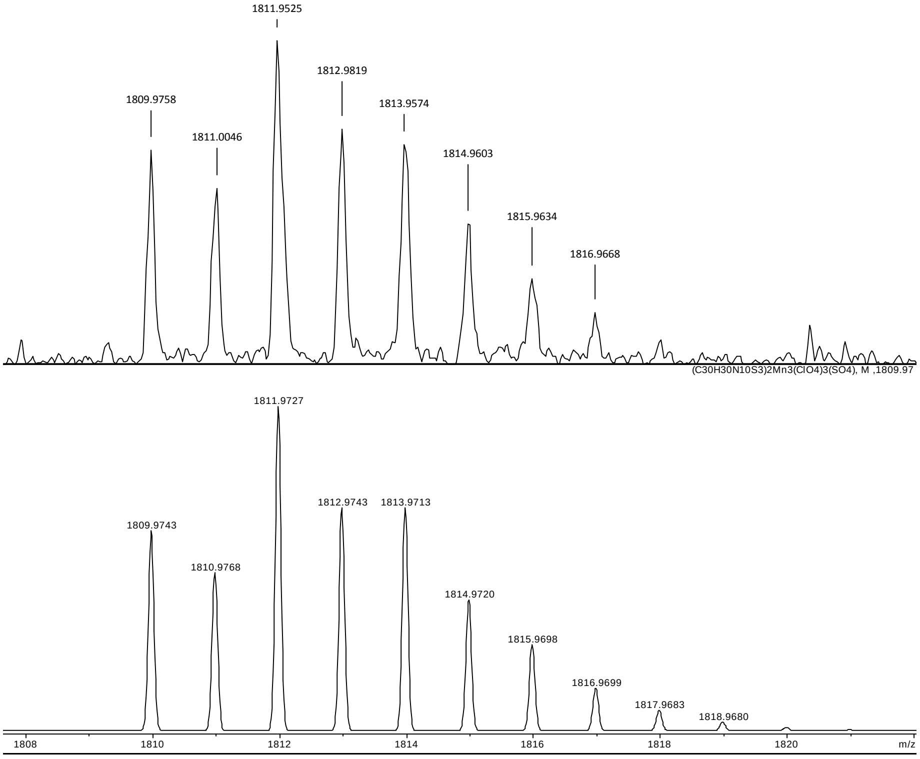

Supplementary Figure 2.10: ESI-MS isotope pattern for *m/z* 1810 {[[**L**$_2$Mn$_3$(SO$_4$)](ClO$_4$)$_2$]}$^{2+}$ (Top: actual signal, bottom: simulated pattern).

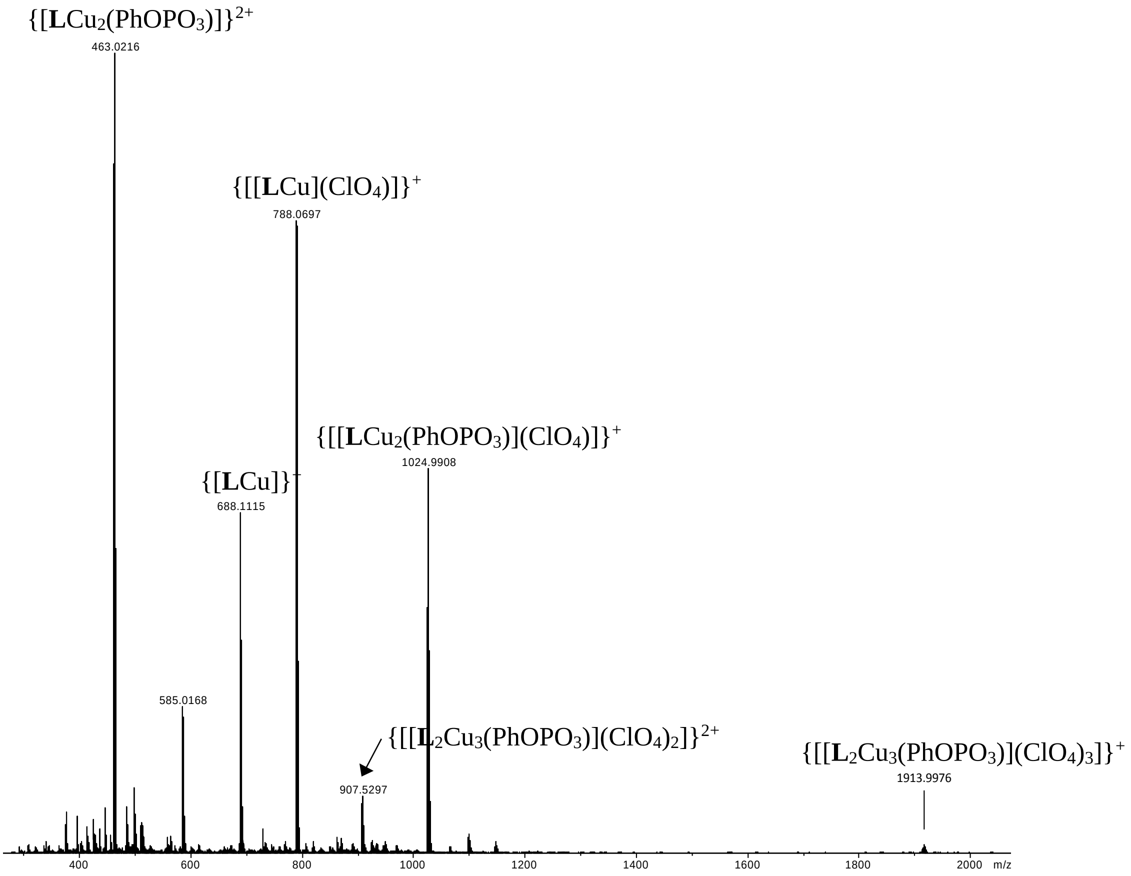

Supplementary Figure 2.11. ESI-MS of [**L**$_2$Cu$_3$(PhOPO$_3$)](ClO$_4$)$_4$.

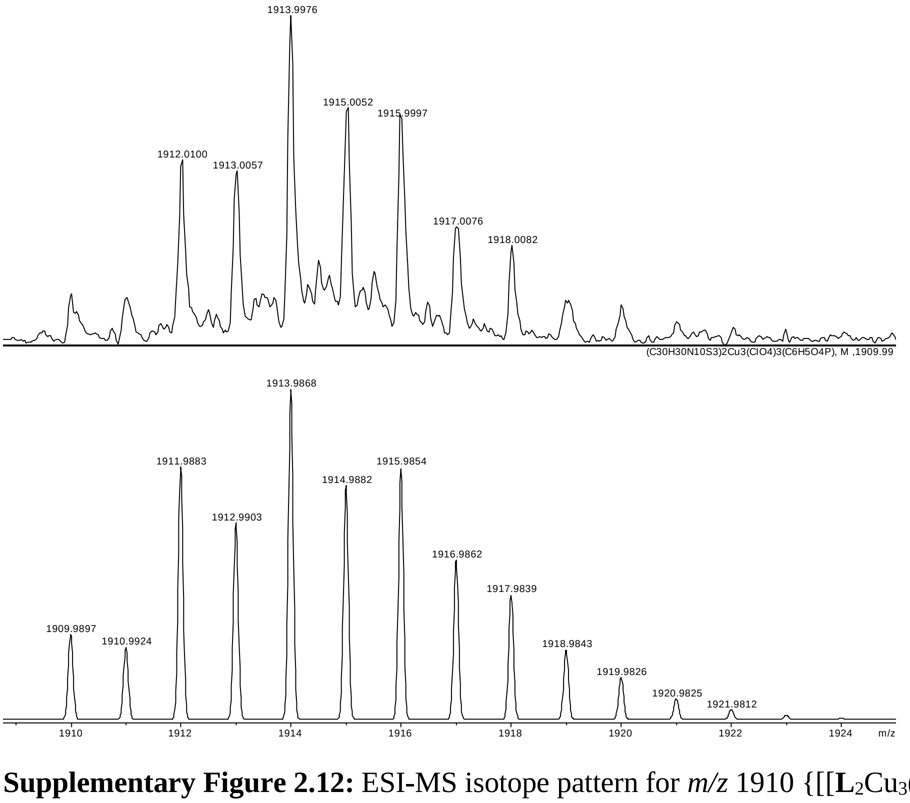

Supplementary Figure 2.12: ESI-MS isotope pattern for *m/z* 1910 {[[**L**$_2$Cu$_3$(PhOPO$_3$)](ClO$_4$)$_2$]}$^+$ (Top: actual signal, bottom: simulated pattern).

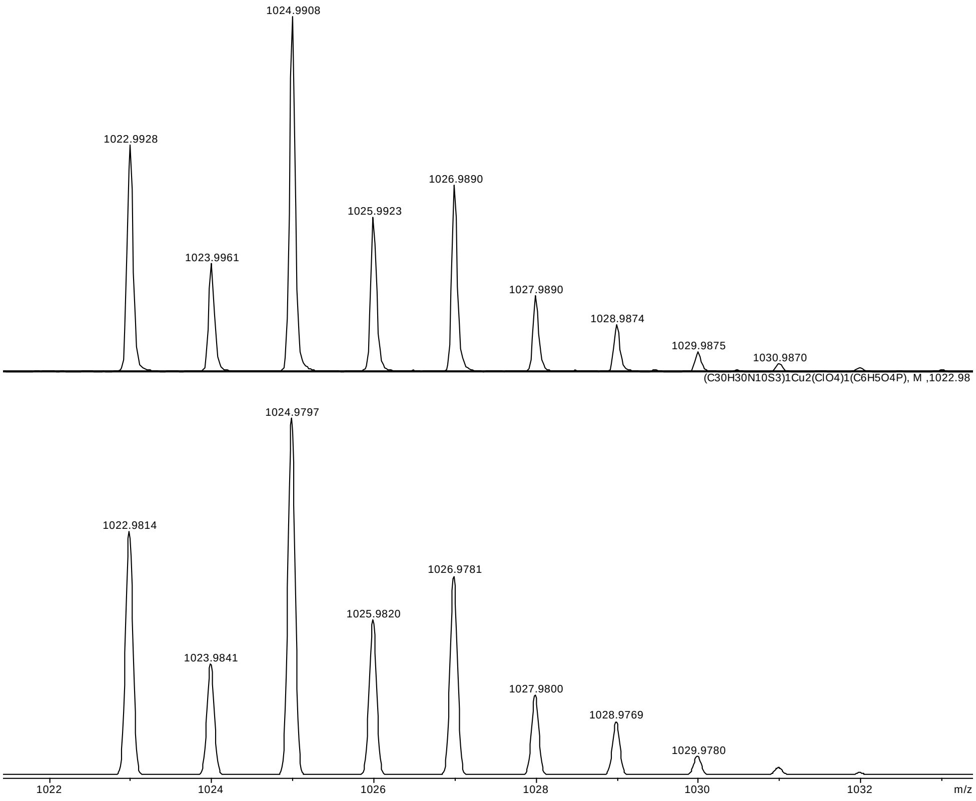

Supplementary Figure 2.13: ESI-MS isotope pattern for *m/z* 1023 {[[**L**Cu_2($PhOPO_3$)](ClO_4)]}$^+$ (Top: actual signal, bottom: simulated pattern).

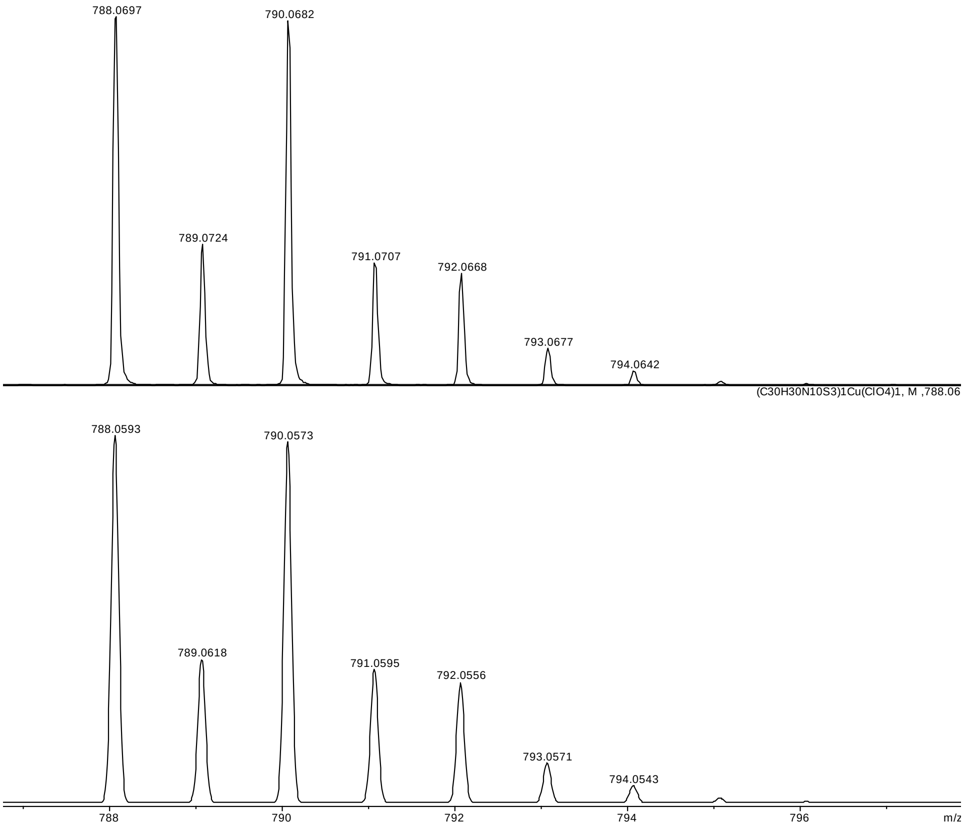

Supplementary Figure 2.14: ESI-MS isotope pattern for *m/z* 788 {[[**L**Cu](ClO_4)]}$^+$ (Top: actual signal, bottom: simulated pattern).

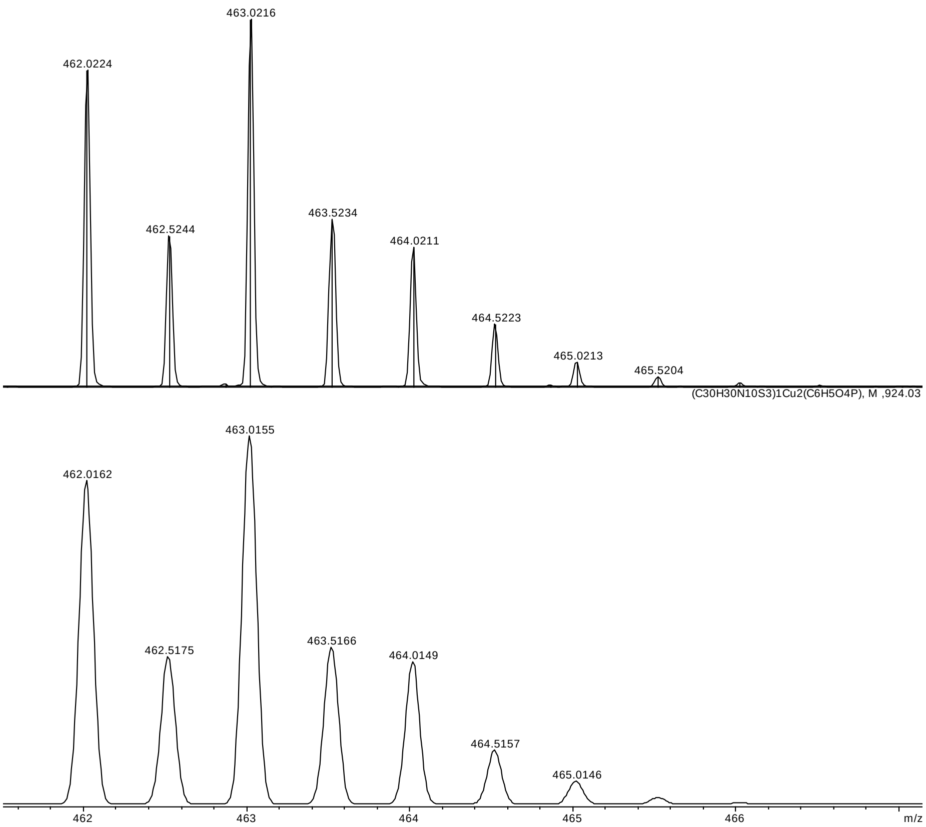

Supplementary Figure 2.15: ESI-MS isotope pattern for *m/z* 924 {[**L**Cu_2($PhOPO_3$)]}$^{2+}$ (Top: actual signal, bottom: simulated pattern).

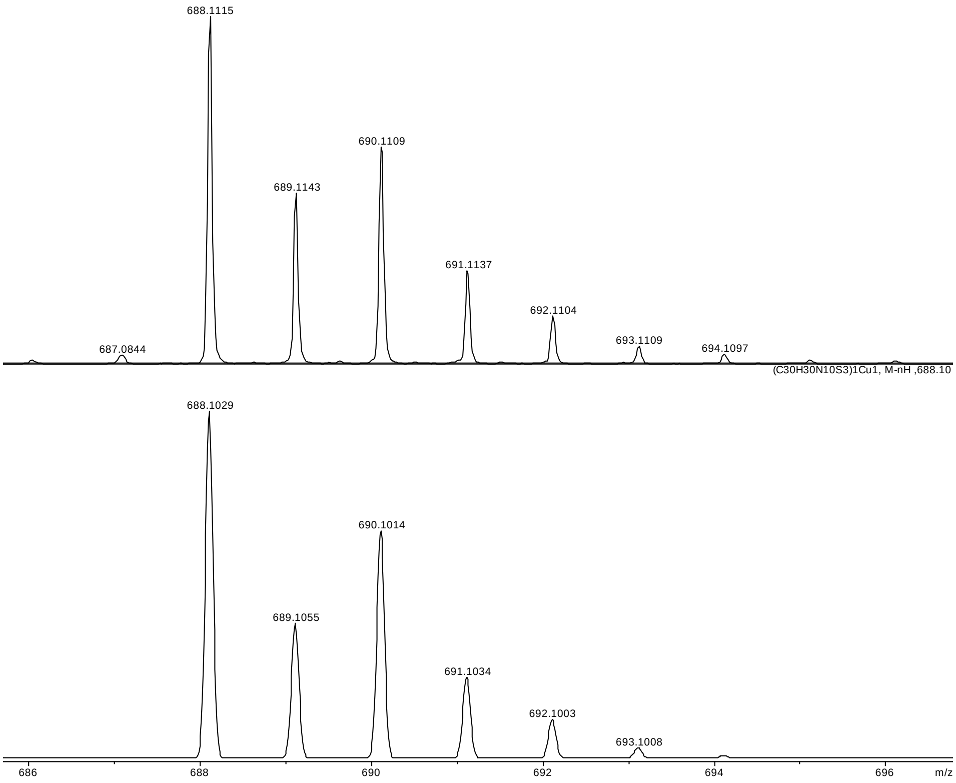

Supplementary Figure 2.16: ESI-MS isotope pattern for *m/z* 688 {[**L**Cu]}$^{+}$ (Top: actual signal, bottom: simulated pattern).

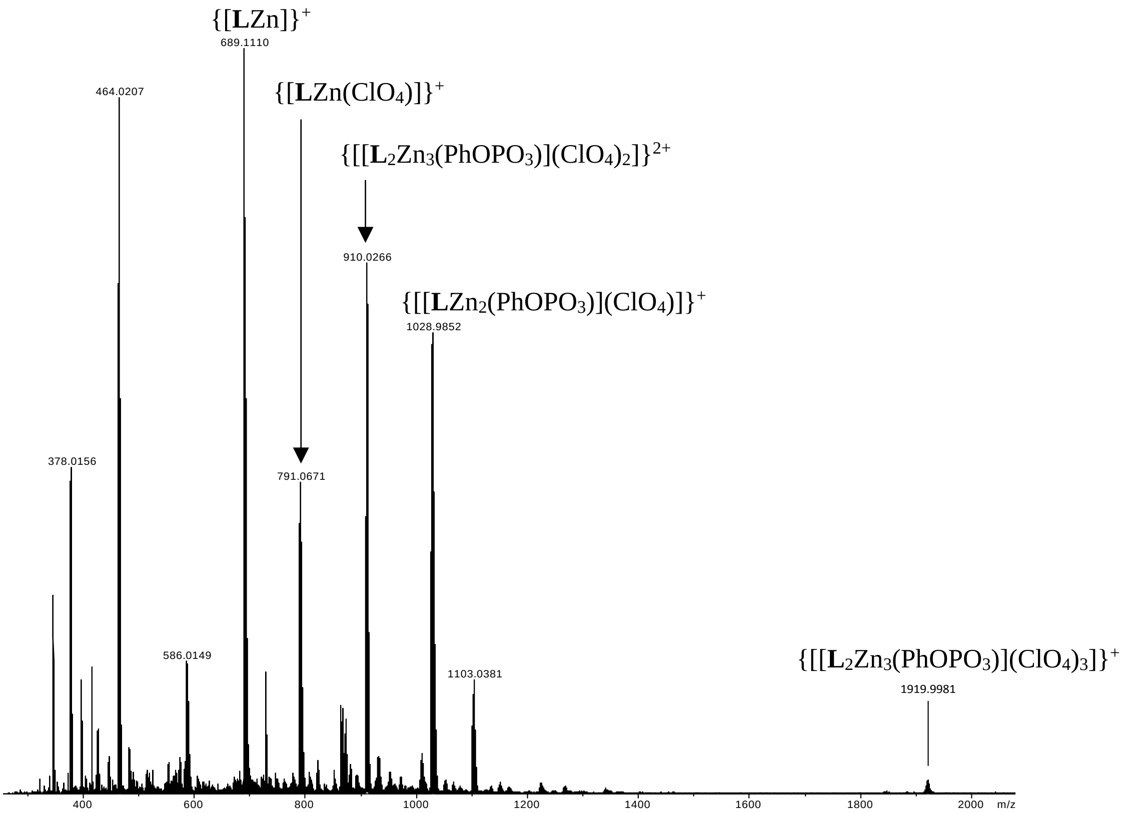

Supplementary Figure 2.17. ESI-MS of $[\mathbf{L}_2Zn_3(PhOPO_3)](ClO_4)_4$.

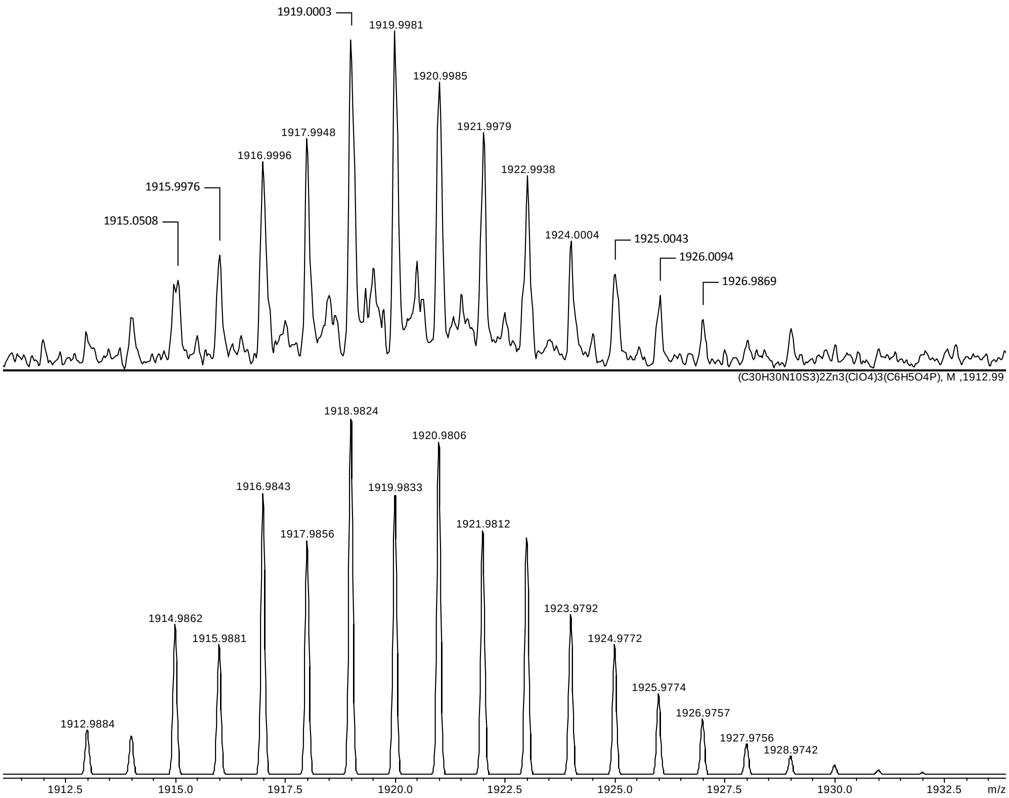

Supplementary Figure 2.18: ESI-MS isotope pattern for *m/z* 1913 $\{[[\mathbf{L}_2Zn_3(PhOPO_3)](ClO_4)_3]\}^+$ (Top: actual signal, bottom: simulated pattern).

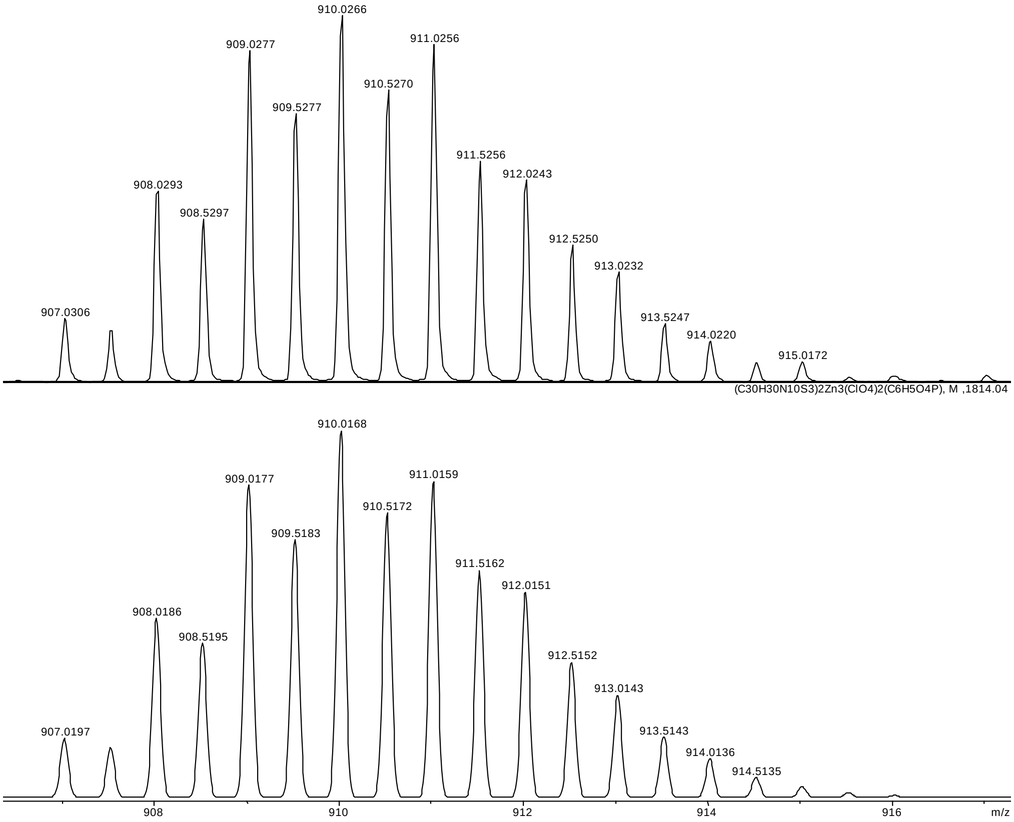

Supplementary Figure 2.19: ESI-MS isotope pattern for *m/z* 907 $\{[[\mathbf{L}_2Zn_3(PhOPO_3)](ClO_4)_2]\}^{2+}$ (Top: actual signal, bottom: simulated pattern).

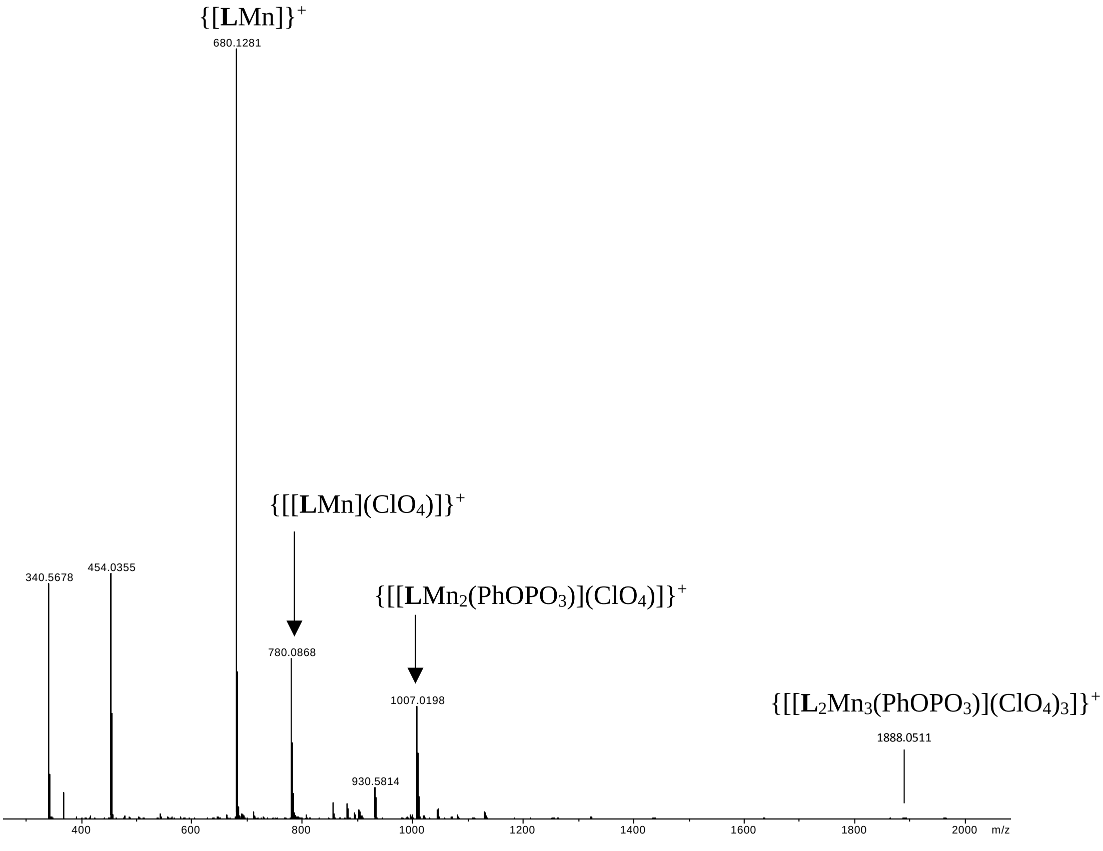

Supplementary Figure 2.20. ESI-MS of $[\mathbf{L}_2Mn_3(PhOPO_3)](ClO_4)_4$.

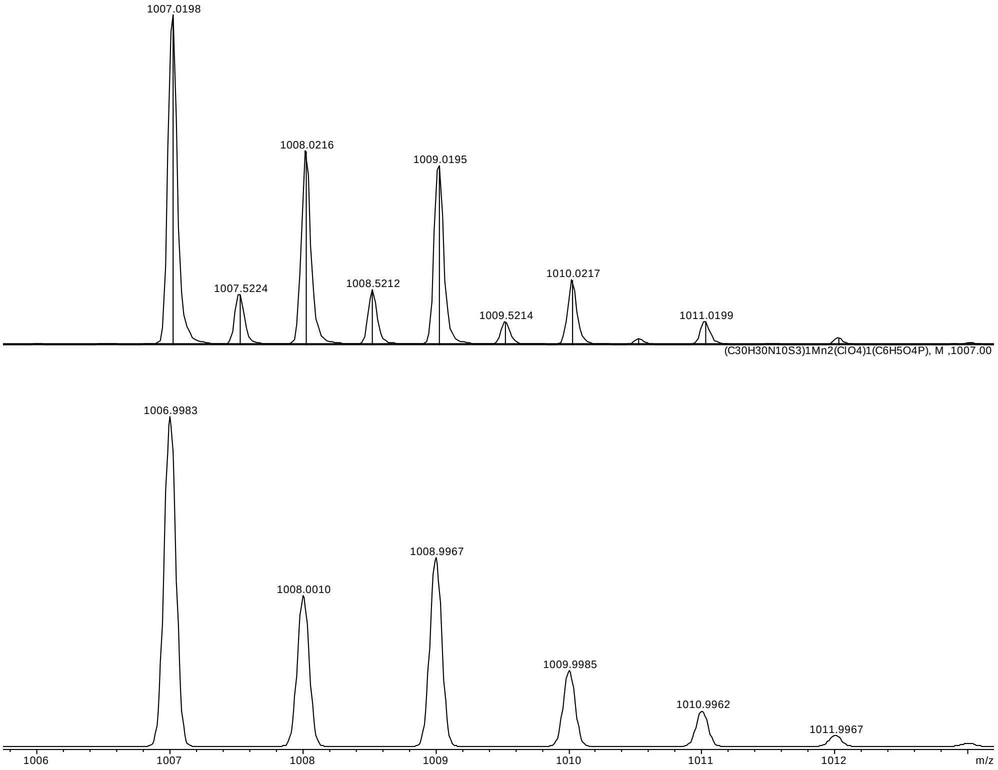

Supplementary Figure 2.21: ESI-MS isotope pattern for *m/z* 1007 {[[**L**Mn_2($PhOPO_3$)](ClO_4)]}$^+$ (Top: actual signal, bottom: simulated pattern).

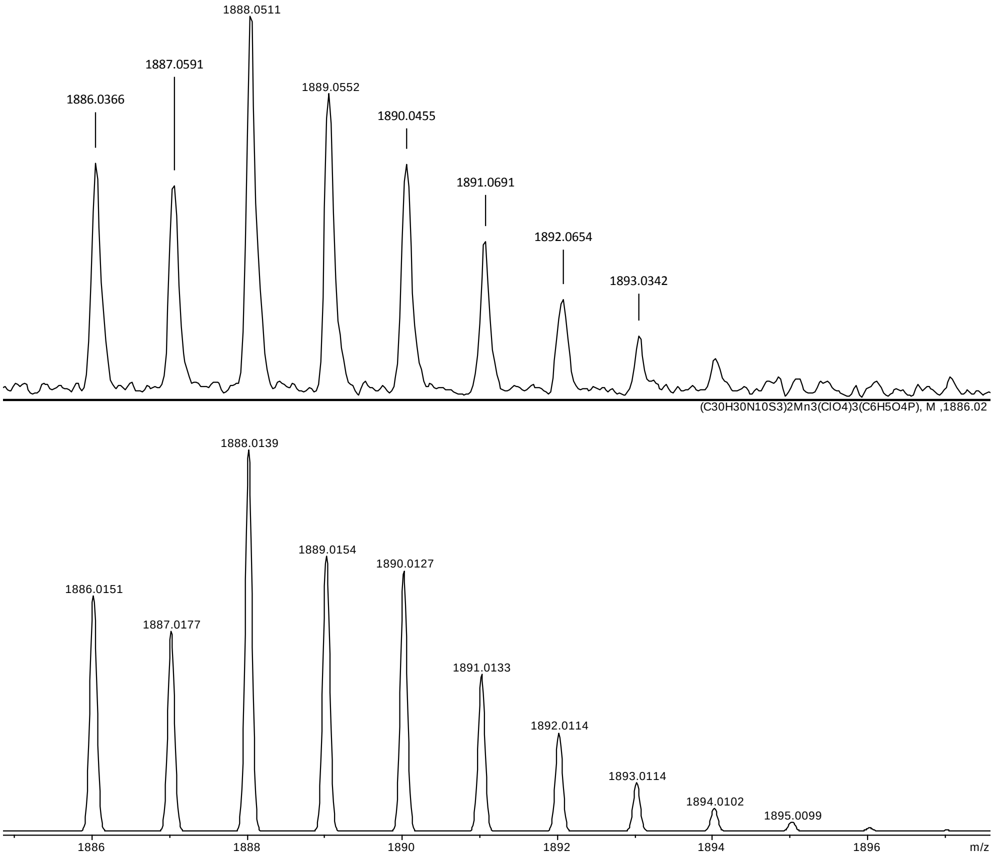

Supplementary Figure 2.22: ESI-MS isotope pattern for *m/z* 1886 {[[L_2Mn_3($PhOPO_3$)](ClO_4)$_3$]}$^+$ (Top: actual signal, bottom: simulated pattern).

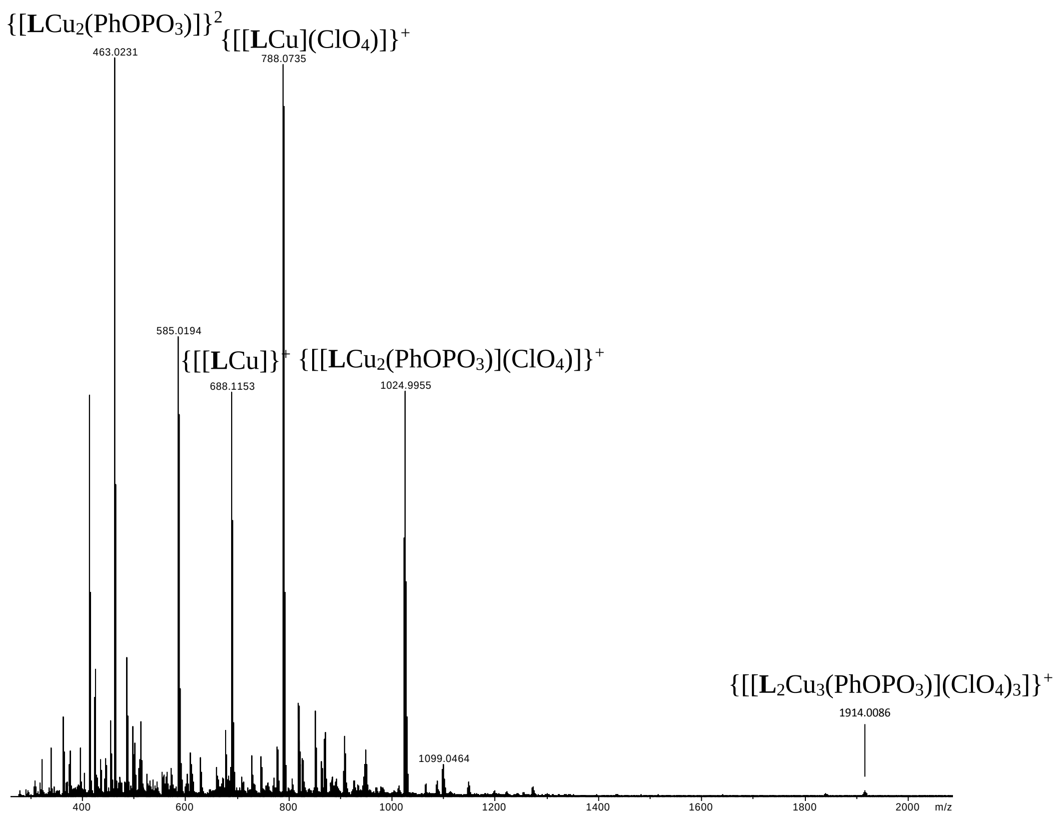

Supplementary Figure 2.23. ESI-MS of [$L_2Cu_3(PhOPO_3)](ClO_4)_4$ after being heated at 80°C for 1 hour.

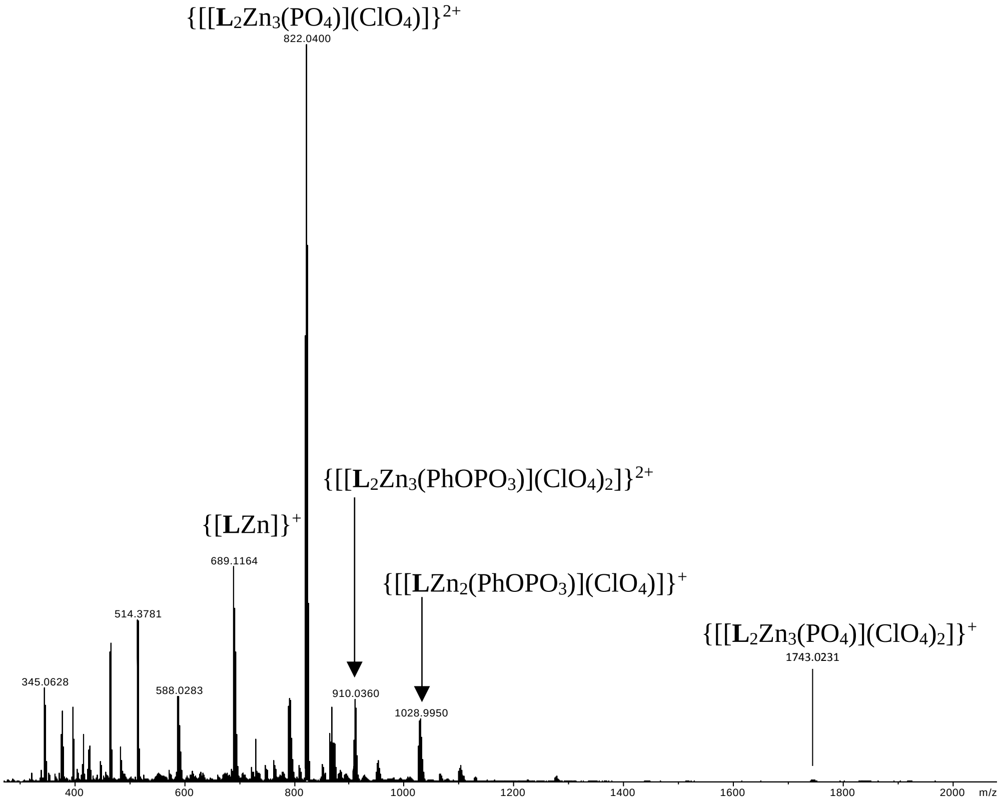

Supplementary Figure 2.24. ESI-MS of [$L_2Zn_3(PhOPO_3)](ClO_4)_4$ after being heated at 80°C for 1 hour.

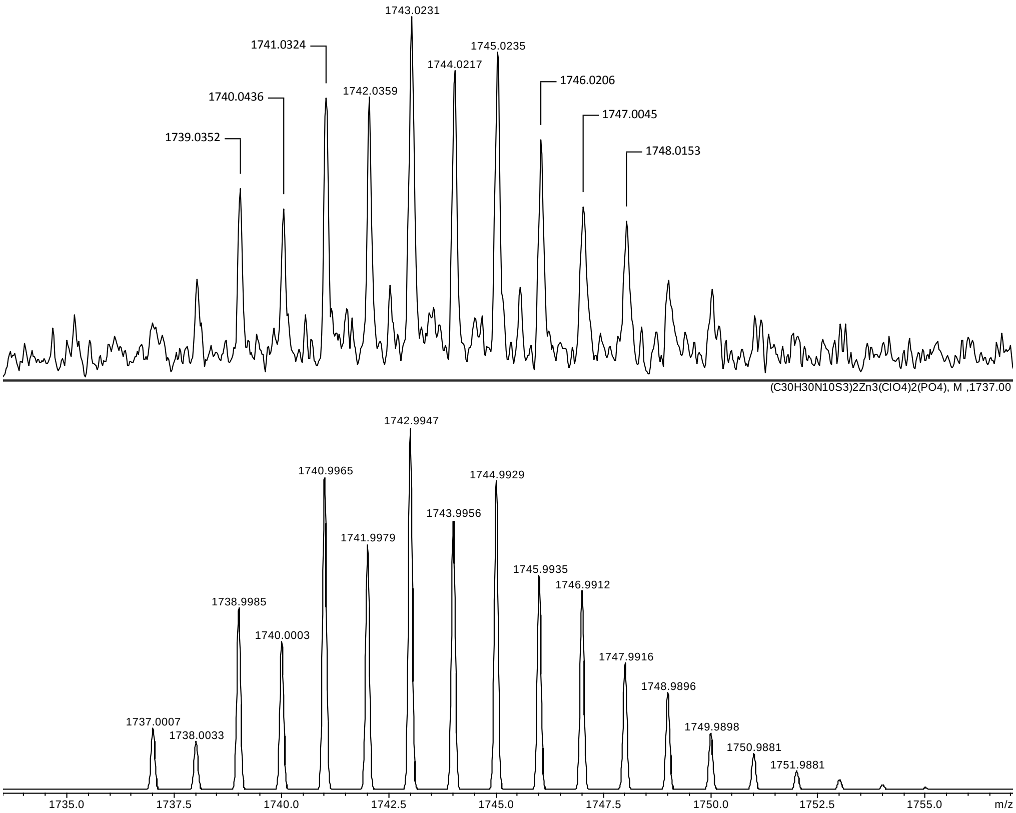

Supplementary Figure 2.25: ESI-MS isotope pattern for *m/z* 1737 {[[**L**$_2$Zn$_3$(PO$_4$)](ClO$_4$)$_2$]}$^+$ (Top: actual signal, bottom: simulated pattern).

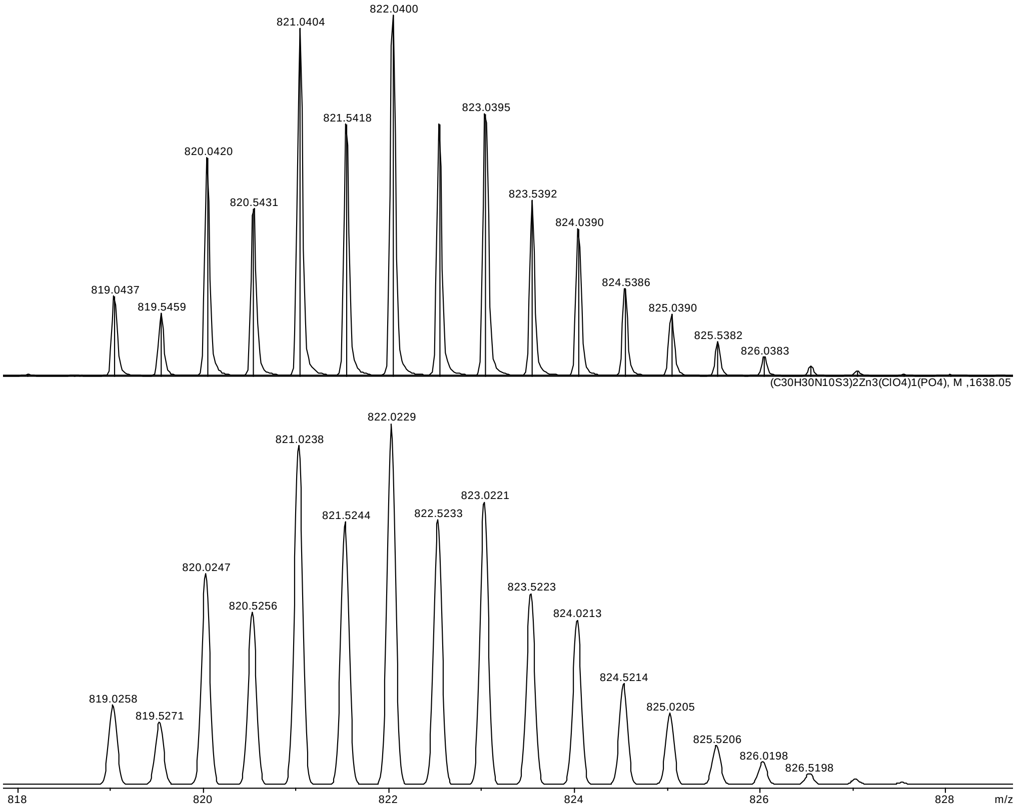

Supplementary Figure 2.26: ESI-MS isotope pattern for *m/z* 819 {[[**L**$_2$Zn$_3$(PO$_4$)](ClO$_4$)]}$^{2+}$ (Top: actual signal, bottom: simulated pattern).

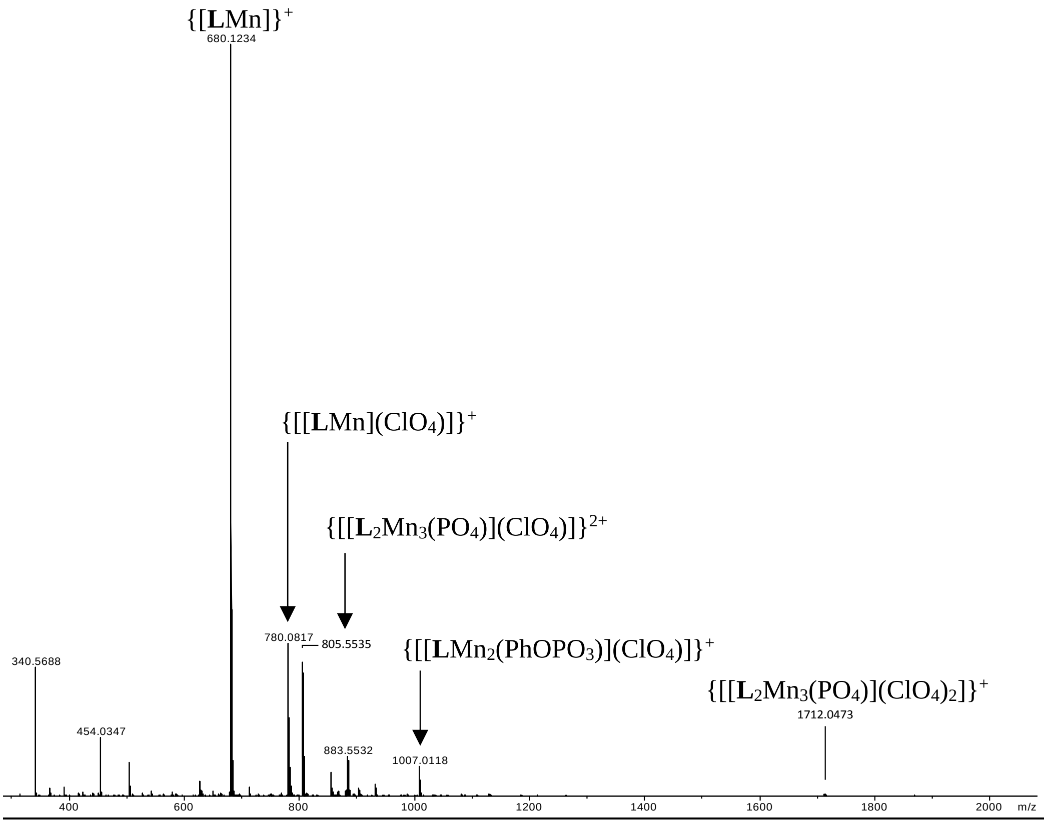

Supplementary Figure 2.27. ESI-MS of [$\mathbf{L}_2Mn_3(PhOPO_3)](ClO_4)_4$ after being heated at 80°C for 1 hour.

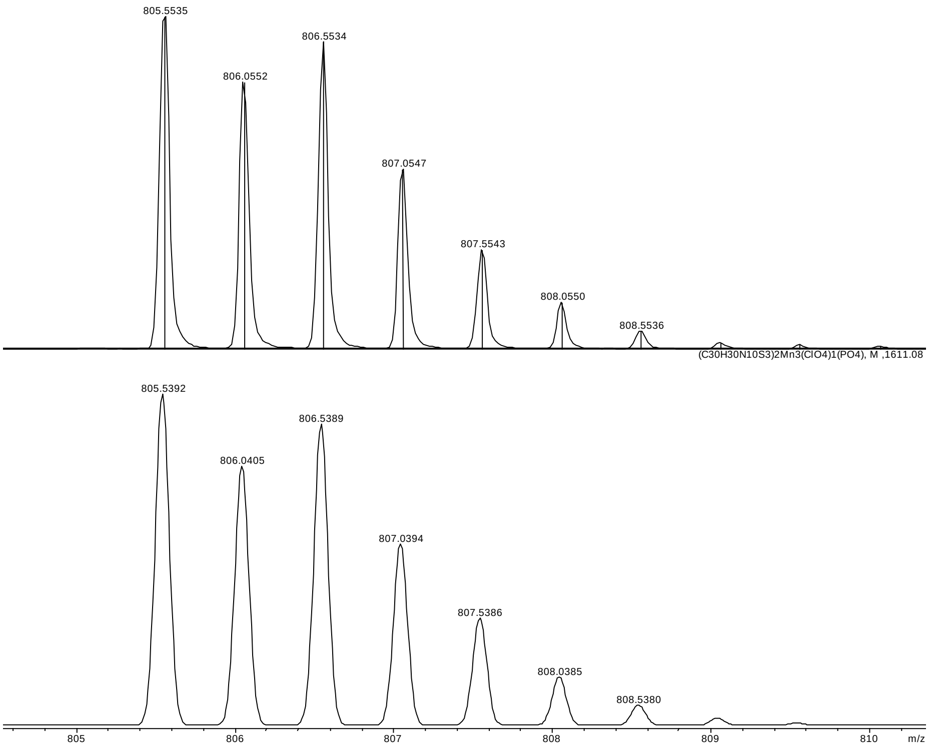

Supplementary Figure 2.28: ESI-MS isotope pattern for *m/z* 805 $\{[[\mathbf{L}_2Mn_3(PO_4)](ClO_4)]\}^{2+}$ (Top: actual signal, bottom: simulated pattern).

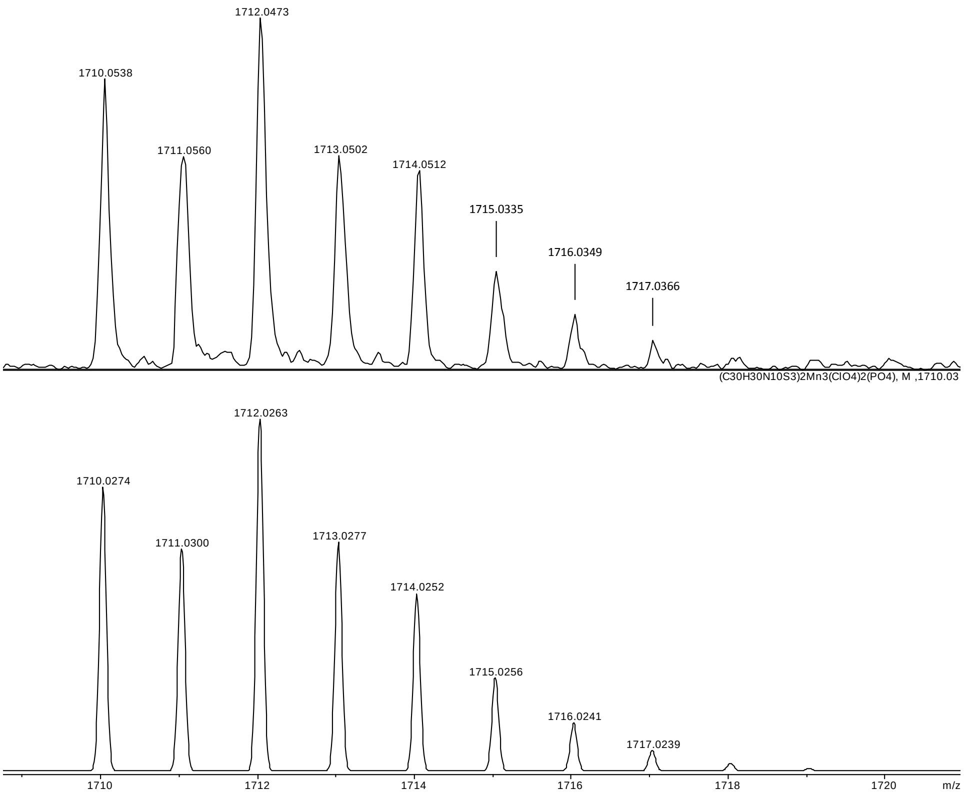

Supplementary Figure 2.29: ESI-MS isotope pattern for *m/z* 1710 $\{[[\mathbf{L}_2Mn_3(PO_4)](ClO_4)_2]\}^+$ (Top: actual signal, bottom: simulated pattern).

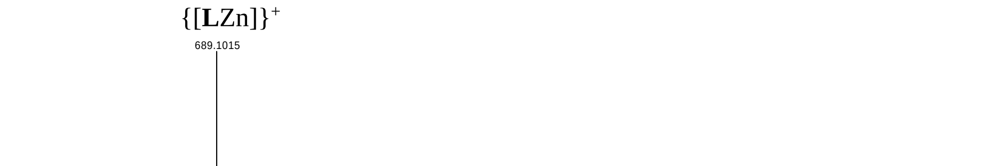

Supplementary Figure 2.30. ESI-MS of $[\mathbf{L}_2Zn_3(ATP)(CH_3CO_2)_2]$.

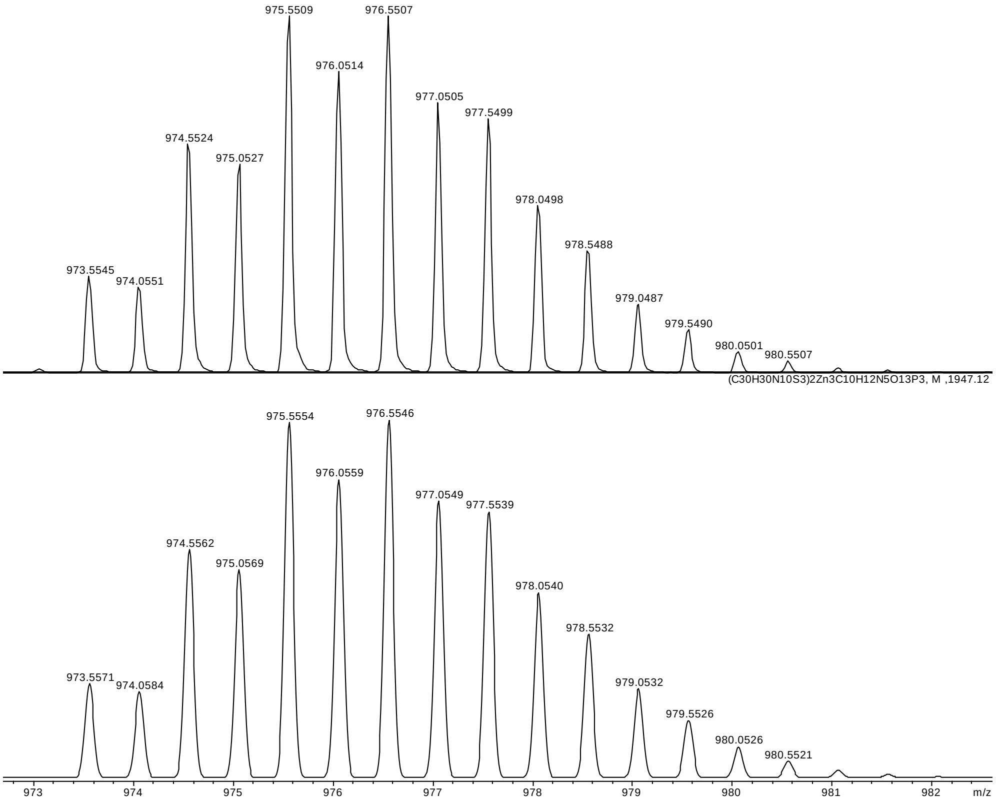

Supplementary Figure 2.31: ESI-MS isotope pattern for *m/z* 973 {[$\mathbf{L}_2Zn_3$(ATP)]}$^{2+}$ (Top: actual signal, bottom: simulated pattern).

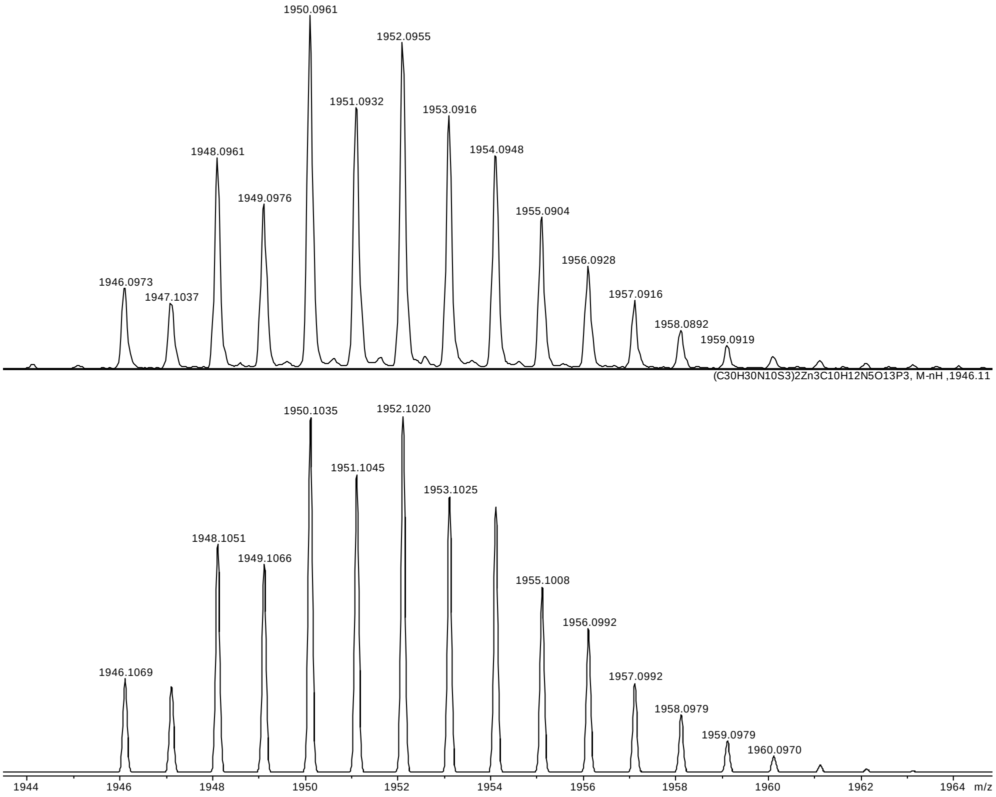

Supplementary Figure 2.32: ESI-MS isotope pattern for *m/z* 1946 {[$\mathbf{L}_2Zn_3$(ATP)]}$^{+}$ (Top: actual signal, bottom: simulated pattern).

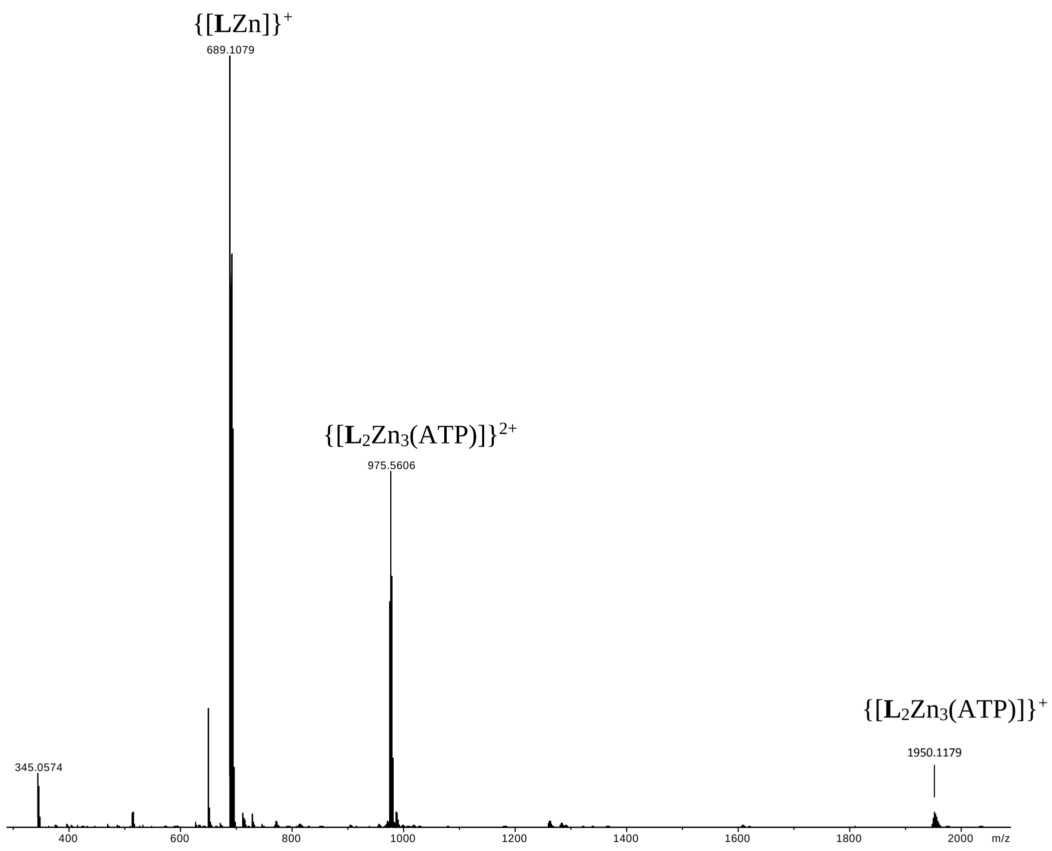

Supplementary Figure 2.33. ESI-MS of [$\mathbf{L}_2Zn_3(ATP)(CH_3CO_2)_2$] after heating at 37°C for 60 hours.

3. Crystallography.

Single crystal X-ray diffraction data was collected at 150(2) K on a Bruker D8 Venture diffractometer equipped with a graphite monochromated Mo(Kα) radiation source and a cold stream of N_2 gas. Solutions were generated by conventional heavy atom Patterson or direct methods and refined by full-matrix least squares on all F^2 data, using SHELXS-97, SHELXL and Olex2 software respectively.[2,3] Absorption corrections were applied based on multiple and symmetry-equivalent measurements using SADABS.[4]Almost all the structures contained some form of disordered ether with solvent molecules and/or counter anions (generally substitutional or rotation disorder). In these cases, the atoms were modelled using the *PART* instruction in the least squares refinement and refined over two positions. The anisotropic displacement parameters were treated with *SIMU*, *DELU* and in some cases *ISOR* where needed. Due to the diffuse nature of the electron density map the hydrogen atoms were not added to disordered solvent molecules. The structure $[L_2Zn_3SO_4](BF_4)_{3.5}$ contained extensively disordered tetrafluoroborate counter anions, one of which refined poorly and was modelled with 50% occupancy.

Compound	$[L_2Cu_3(PhOPO_3)](BF_4)_4$·2 MeCN ·$3H_2O$	$[LZn](ClO_4)_2$·Me_2CO	$[L_2Zn_3(PO_4)](ClO_4)_3$·2MeCN	$[L_2Zn_3(SO_4)](BF_4)_{3.5}$· MeCN
Formula	$C_{70}H_{65}B_4Cu_3F_{16}N_{22}O_7PS_6$	$C_{33}H_{36}Cl_2N_{10}O_{9.50}S_3Zn$	$C_{64}H_{66}Cl_3N_{22}O_{16}PS_6Zn_3$	$C_{62}H_{60}B_{3.5}F_{14}N_{21}O_4S_7Zn_3$
M	2087.72	957.17	1925.17	1887.82
Crystal system	Triclinic	Triclinic	Monoclinic	Monoclinic
Space group	P -1	P -1	P 21/c	C 1 2/c 1
a (Å)	13.6338(10)	12.1349(5)	19.6676(8)	36.775(7)
b (Å)	14.3519(10)	13.5143(6)	22.0889(8)	20.777(4)
c (Å)	23.1506(18)	14.0968(6)	17.9988(8)	27.448(5)
α (°)	76.225(3)	70.316(2)	90	90
β (°)	83.869(3)	65.002(2)	98.433(2)	115.095(9)
γ (°)	85.279(3)	80.535(2)	90	90
V ($Å^3$)	4366.9(6)	1972.14(15)	7734.8(5)	18993(6)
Z	2	2	4	8
ρ_{calc} (Mg cm^{-1})	1.5876	1.612	1.653	1.3203
F (000)	2118.8058	984	3936	7669.9894
Crystal dimensions (mm)	0.18, 0.11, 0.03	0.22, 0.20, 0.09	0.12, 0.07, 0.07	0.18, 0.15, 0.10
Reflections measured	55621	30907	80079	121061
Range	$2.04 \leq \theta \geq 28.32^{\circ}$	$2.48 \leq \theta \geq 33.10^{\circ}$	$2.34 \leq \theta \geq 32.13^{\circ}$	$2.29 \leq \theta \geq 27.43^{\circ}$
hkl range indeces	$-18 \leq h \geq 18$, $-19 \leq k \geq 19$, $-30 \leq l \geq 30$	$-18 \leq h \geq 18$, $-20 \leq k \geq 19$, $-21 \leq l \geq 20$	$-28 \leq h \geq 28$, $-32 \leq k \geq 32$, $-22 \leq l \geq 26$	$-49 \leq h \geq 48$, $-27 \leq k \geq 27$, $-36 \leq l \geq 36$
N° independent reflections	21484	14937	25665	23507
Reflections with $I > 2\sigma(I)$	14358	10352	17419	15289
R_{int}	0.0771	0.0445	0.0636	0.0592
Final R_1 values	0.0725	0.0499	0.0479	0.0653
Final $wR(F^2)$ values	0.1969	0.1159	0.1011	0.1851
Final R_1 values (all data)	0.1124	0.0839	0.0857	0.1050

Final $wR(F^2)$ values (all data)	0.2326	0.1344	0.1155	0.2298
GOF	1.0879	1.020	1.015	1.1131
Refined parameters	1209	553	1057	1111
Restraints	81	24	42	82
Largest peak and hole (e Å^{-3})	1.5905, -1.5497	0.983, -0.920	1.147, -0.751	2.2859, -0.9214
CCDC number	2005186	2005132	2005189	2005221

Supplementary Table 3.1. X-ray crystallographic data for $[\mathbf{L}_2Cu_3(PhOPO_3)]^{4+}$, $[\mathbf{L}Zn]^{2+}$, $[\mathbf{L}_2Zn_3(PO_4)]^{3+}$ and $[\mathbf{L}_2Zn_3(SO_4)]^{4+}$.

Compound	$[LMn](ClO_4)_2 \cdot MeCN \cdot H_2O$	$[L_2Mn_3(PO_4)](ClO_4)_3 \cdot MeCN \cdot 2MeOH$	$[L_2Mn_3(SO_4)](ClO_4)_4 \cdot 3H_2O \cdot 2MeCN$
Formula	$C_{32}H_{39}Cl_2MnN_{11}O_{11}S_3$	$C_{65.804}H_{75}Cl_3Mn_3N_{21}O_{21}PS_6$	$C_{63.636}H_{67}Cl_4Mn_3N_{21.818}O_{26.085}S_7$
M	975.78	1990.65	2085.91
Crystal system	Monoclinic	Triclinic	Orthorhombic
Space group	P 1 21/c 1	P -1	P b c a
a (Å)	25.3201(10)	13.769(3)	26.4653(7)
b (Å)	17.9313(7)	15.509(3)	23.3289(5)
c (Å)	19.1082(6)	20.200(4)	28.1102(6)
α (°)	90	91.564(8)	90
β (°)	109.6360(10)	104.592(9)	90
γ (°)	90	96.722(11)	90
V (Å^3)	8171.0(5)	4138.4(13)	17355.4(7)
Z	8	2	8
ρ_{calc} (Mg cm^{-1})	1.5863	1.5974	1.5965
F (000)	4033.8382	2049.1485	8545.5634
Crystal dimensions (mm)	0.20, 0.18, 0.05	0.16, 0.12, 0.12	0.23, 0.21, 0.18
Reflections measured	67342	64143	164752
Range	$2.05 \leq \theta \geq 30.55^\circ$	$2.32 \leq \theta \geq 33.01^\circ$	$2.27 \leq \theta \geq 33.13^\circ$
hkl range indeces	$-27 \leq h \geq 36$, $-25 \leq k \geq 21$, $-27 \leq l \geq 27$	$-21 \leq h \geq 21$, $-23 \leq k \geq 23$, $-30 \leq l \geq 28$	$-39 \leq h \geq 40$, $-35 \leq k \geq 32$, $-43 \leq l \geq 40$
N° independent reflections	24905	30920	32977
Reflections with $I > 2\sigma(I)$	16699	21994	23821
R_{int}	0.0494	0.0453	0.0487
Final R_1 values	0.0580	0.0500	0.0544
Final $wR(F^2)$ values	0.1363	0.1219	0.1328
Final R_1 values (all data)	0.0973	0.0797	0.0839
Final $wR(F^2)$ values (all data)	0.1606	0.1464	0.1535
GOF	1.0879	1.0740	1.0833

Refined parameters	1140	1109	1258
Restraints	15	28	24
Largest peak and hole (e Å^{-3})	1.1456, -1.0796	1.3806, -1.6085	1.5168, -1.2101
CCDC number	2005133	2005190	2005222

Supplementary Table 3.1. X-ray crystallographic data for [**L**Mn]$^{2+}$, [**L**$_2$Mn$_3$(PO$_4$)]$^{3+}$ and [**L**$_2$Mn$_3$(SO$_4$)]$^{4+}$.

4. NMR studies.

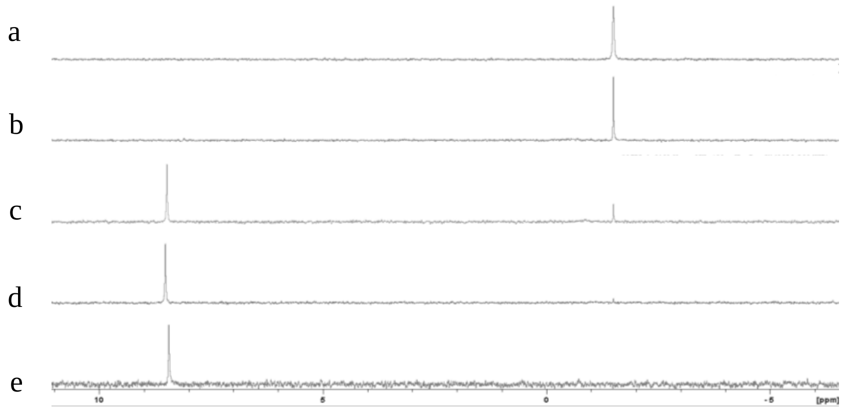

Supplementary Figure 4.1. ^{13}P NMR spectrum (600 µL HEPES 60 mmol (@ *p*H 7.4) and 200 µL d$_6$-DMSO) of a) PhOPO$_3$Na$_2$, 44hrs incubated @ 37°C, b) [**L**$_2$Zn$_3$]$^{6+}$ + PhOPO$_3$Na$_2$, T = 0, c) [**L**$_2$Zn$_3$]$^{6+}$ + PhOPO$_3$Na$_2$, T = 19 hrs incubated @ 37°C, d) [**L**$_2$Zn$_3$]$^{6+}$ + PhOPO$_3$Na$_2$, T = 44 hrs incubated @ 37°C, e) [**L**$_2$Zn$_3$]$^{6+}$ + Bu$_4$NH$_2$PO$_4$. The NMR solutions were prepared by dissolving **L** and Zn(C$_2$H$_3$O$_2$)$_2$ (in a ratio of 1:1.5) in d$_6$-DMSO (200 µL). The relevant anion (0.5 equiv w.r.t ligand) was dissolved in HEPES (600 µL, @ *p*H 7.4) and these solutions were then combined. Heating of the sample was carried out by incubating the sample @ 37 °C. HEPES = (4-(2-hydroxyethyl)-1-piperazineethanesulfonic acid).

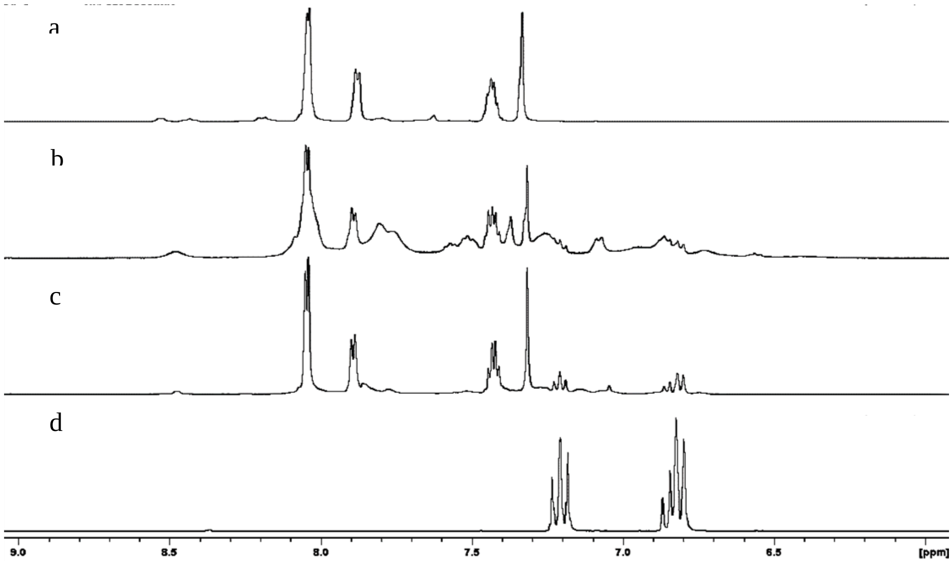

Supplementary Figure 4.2. ^{1}H NMR spectrum (10% D$_2$O in CD$_3$CN) of a) [**L**$_2$Zn$_3$]$^{6+}$ + Bu$_4$NH$_2$PO$_4$, b) [**L**$_2$Zn$_3$]$^{6+}$ + PhOPO$_3$Na$_2$, T = 0, c) [**L**$_2$Zn$_3$]$^{6+}$ + PhOPO$_3$Na$_2$, T = 1 hr incubated @ 80°C, d) phenol. The NMR solutions were prepared by dissolving **L** and Zn(C$_2$H$_3$O$_2$)$_2$ (in a ratio of 1:1.5) in CD$_3$CN (600 µL). The relevant anion (0.5 equiv w.r.t ligand) was dissolved in D$_2$O (60 µL) and these solutions were then combined. Heating of the sample was carried out @ 80 °C in a water bath for the specified amount of time.

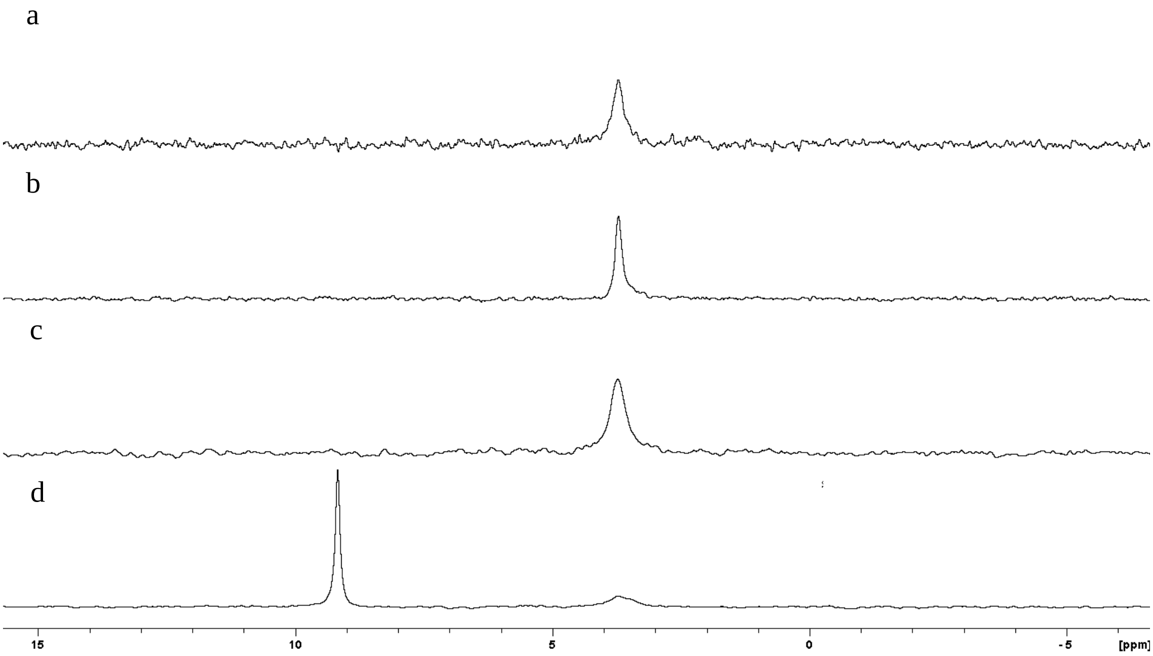

Supplementary Figure 4.3. ^{31}P NMR spectra (600 µL HEPES (@ *p*H 7.4) and 200 µL d$_6$-DMSO) of a) [$\mathbf{L}_2Zn_3$]$^{6+}$ + peptide-OPO_3^{2-} (HMRSAMS*GLHLVKRR) t = 0, b) [$\mathbf{L}_2Zn_3$]$^{6+}$ + peptide-OPO_3^{2-} t = 48 hrs @ 37°C, c) [$\mathbf{L}_2Zn_3$]$^{6+}$ + peptide-OPO_3^{2-} t = 48 hrs @ 37°C and then 2 hrs 80°C, d) [$\mathbf{L}_2Zn_3$]$^{6+}$ + peptide-OPO_3^{2-} + $Bu_4NH_2PO_4$ with the shift at ~9 ppm corresponding to [$\mathbf{L}_2Zn_3(PO_4)$]$^{3+}$. The NMR solutions were prepared by dissolving **L** and $Zn(C_2H_3O_2)_2$ (in a ratio of 1:1.5) in d$_6$-DMSO (200 µL). The peptide-OPO_3^{2-} (0.5 equiv w.r.t ligand) was dissolved in HEPES (600 µL, 60 mmol @ *p*H 7.4) and these solutions were then combined. Heating of the sample was carried out by incubating the sample @ 37 °C and a further 2 hrs @ 80 °C in a water bath. HEPES = (4-(2-hydroxyethyl)-1-piperazineethanesulfonic acid).

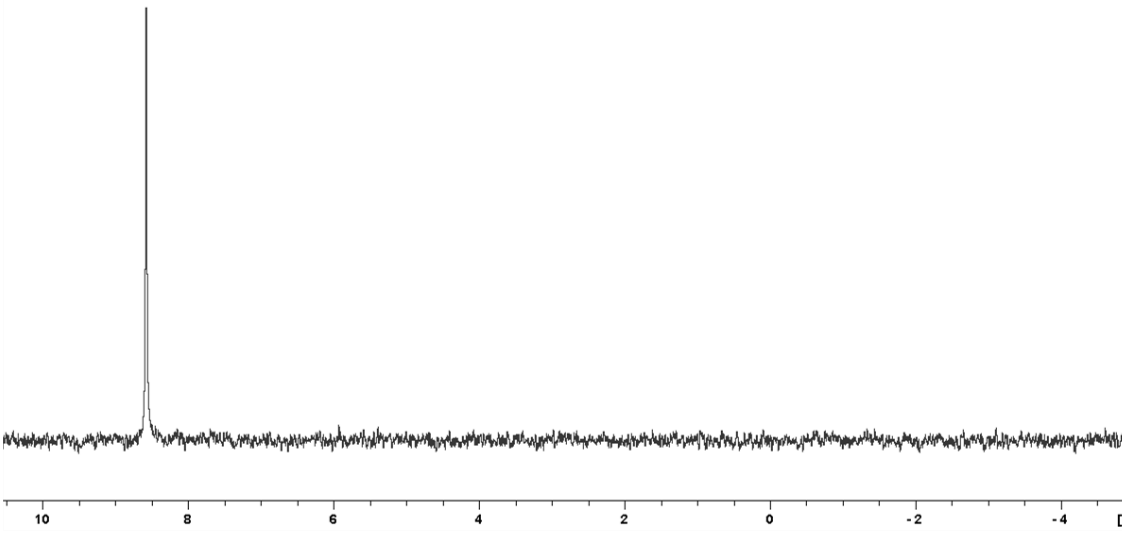

Supplementary Figure 4.4. ^{31}P NMR spectra (600 µL HEPES (60 mmol @ *p*H 7.4) and 200 µL d$_6$-DMSO) of [$\mathbf{L}_2Zn_3$]$^{6+}$ showing the signal at ~ 9 ppm corresponding to [$\mathbf{L}_2Zn_3(PO_4)$]$^{3+}$ demonstrating no competition from HEPES as no observed signal at ~ 0 ppm corresponding to free phosphate. Solution contains an approximately 10-fold excess of HEPES.

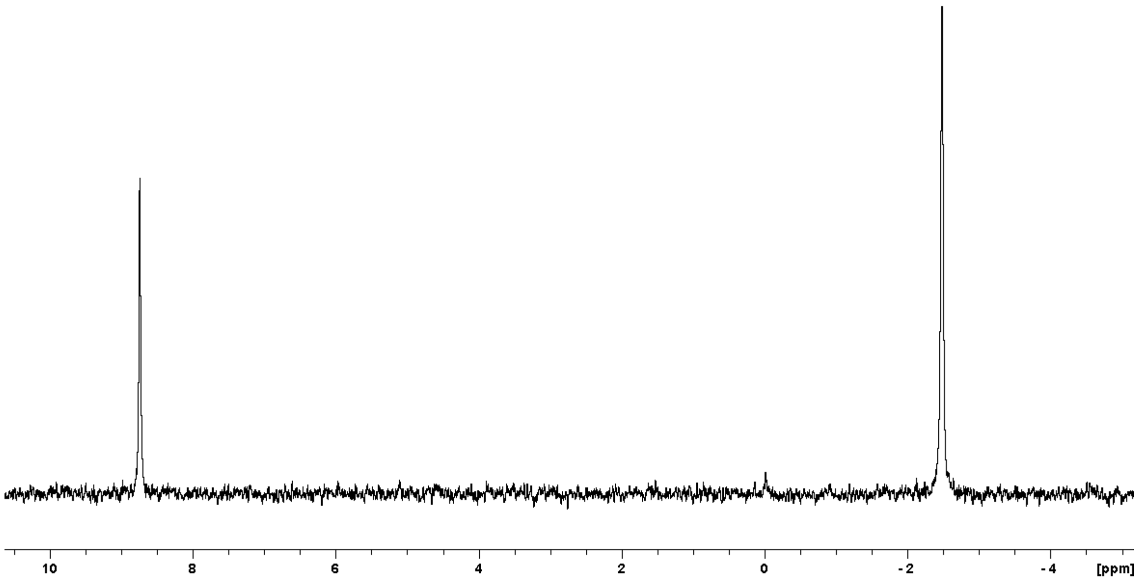

Supplementary Figure 4.5. ^{31}P NMR spectra (600 µL HEPES (60 mmol @ *p*H 7.4) and 200 µL d_6-DMSO) of $[L_2Zn_3(PO_4)]^{3+}$ plus one equivalent of Na_2O_2POPh showing the signal at ~ 9 ppm corresponding to $[L_2Zn_3(PO_4)]^{3+}$ and -2.5 ppm corresponding to Na_2O_2POPh. No change in either the ratio of $[L_2Zn_3(PO_4)]^{3+}$ to Na_2O_2POPh and no signal at ~ 0 ppm (corresponding to free PO_4^{3-}) was observed, indicating lack of catalytic activity.

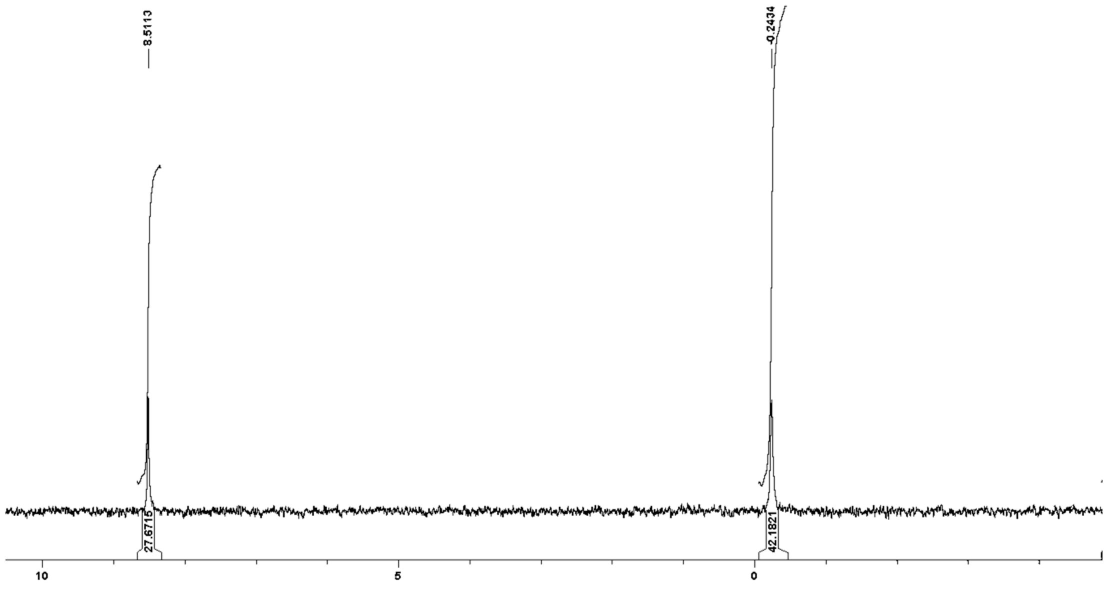

Supplementary Figure 4.6. ^{31}P NMR spectra (600 µL HEPES (60 mmol @ *p*H 7.4) and 200 µL d_6-DMSO) of $[L_2Zn_3(SO_4)]^{4+}$ plus one equivalent of NaH_2PO_4 showing the signal at ~ 9 ppm corresponding to $[L_2Zn_3(PO_4)]^{3+}$ and ~ 0 ppm corresponding to unbound NaH_2PO_4. This indicates that a proportion, but not all, of the sulfate has been displaced but the presence of "free" NaH_2PO_4 demonstrates that $[L_2Zn_3(SO_4)]^{4+}$ still persists as the major species (~ 66%).

5. UV-Vis studies of the hydrolysis of disodium 4-nitrophenylphosphate.

UV-Vis studies were carried out on a Cary 60 UV-Vis spectrophotometer with Thermo cycler attachment and the wavelength range of 800 – 250 nm was scanned every hour for 43 hours whilst the solution was heated at a constant temperature of 37 °C. Progress of hydrolysation of 4-nitrophenyl phosphate to 4-nitrophenol was measured by monitoring the increase in absorption at the wavelength of 400 nm (typical for 4-nitrophenol). HEPES buffer was prepared at *p*H 7.4.

For [[$\mathbf{L}_2Cu_3$]($C_2H_3O_2$)$_6$], [[$\mathbf{L}_2Zn_3$]($C_2H_3O_2$)$_6$] and [[$\mathbf{L}_2Mn_3$]($C_2H_3O_2$)$_6$]:

Solutions were prepared by dissolving the ligand **L** (3.1 mg, 0.005 mmol) and the relevant metal salt (1.5 equiv w.r.t. ligand) in DMSO (5 mL). 4-nitrophenyl phosphate (18.6 mg, 0.050 mmol) was dissolved in HEPES buffer (~10 mL, 60 mmol) These solutions were then combined and made up volumetrically to 50 mL with HEPES buffer.

For [[$\mathbf{L}_2Cu_3(PO_4)$]($C_2H_3O_2$)$_3$], [[$\mathbf{L}_2Zn_3(PO_4)$]($C_2H_3O_2$)$_3$] and [[$\mathbf{L}_2Mn_3(PO_4)$]($C_2H_3O_2$)$_3$]:

Solutions were prepared by dissolving the ligand **L** (3.1 mg, 0.005 mmol) and the relevant metal salt (1.5 equiv w.r.t. ligand) in DMSO (5 mL). Tetrabutylammonium phosphate (0.84 mg, 0.0025 mmol) was dissolved in HEPES buffer (~1 mL, 60 mmol) and these solutions were combined. The 4-nitrophenyl phosphate (18.6 mg, 0.050 mmol) was dissolved separately in HEPES buffer (~10 mL) before being combined with the solution containing the complex and making up volumetrically to 50 mL with HEPES buffer.

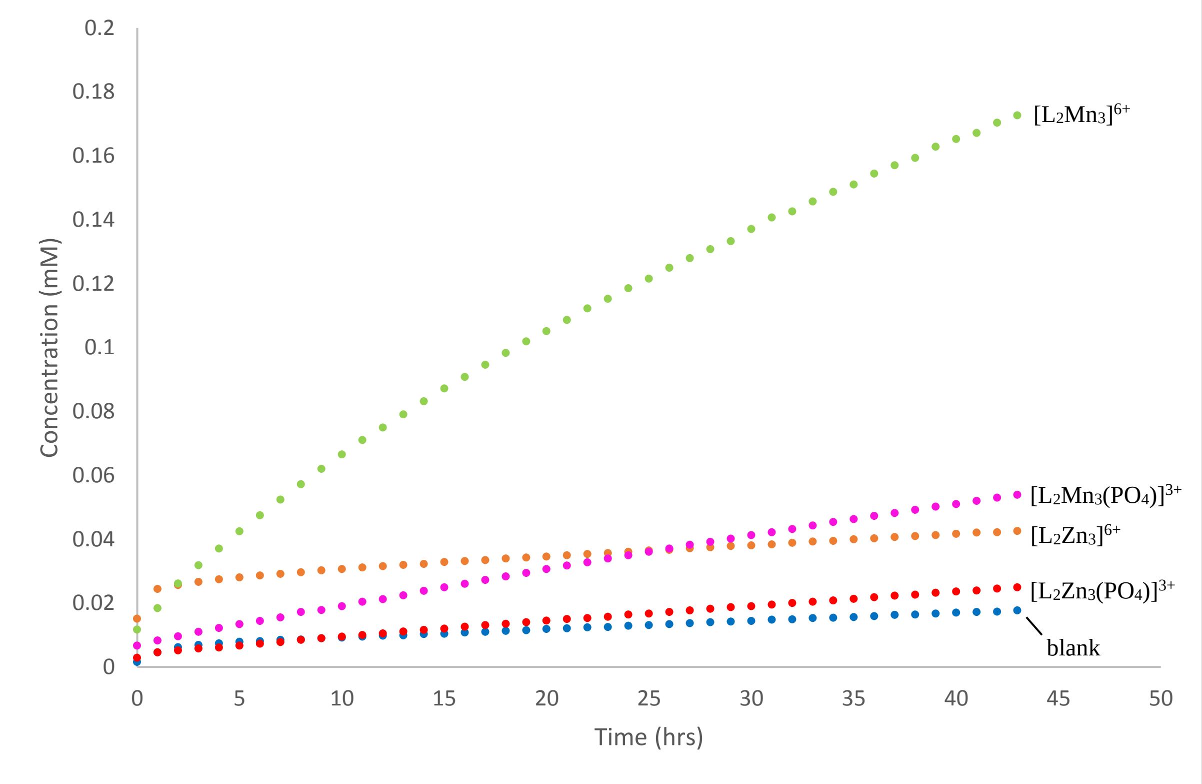

Supplementary Figure 5.1. Concentration of 4-nitrophenol (mM) verses time in the presence of different complexes.

Compound	Concentration of 4-nitrophenol (mM) after 24 hours at 37°C
blank	0.0129
$[L_2Zn_3](CH_3CO_2)_6$	0.0361
$[L_2Mn_3](CH_3CO_2)_6$	0.1185
$[L_2Zn_3(PO_4)](CH_3CO_2)_3$	0.0164
$[L_2Mn_3(PO_4)](CH_3CO_2)_3$	0.0350

Supplementary Figure 5.2. Concentration of 4-nitrophenol (nM) at 24 hr at 37°C.

Calibration standards:

Calibration of 4-nitrophenol was carried out by observing the 400 nm wavelength of a series of standards over the range of 0.01, 0.025, 0.05, 0.075, 0.1, 0.15, 0.175, 0.200, 0.225 and 0.250 mM.

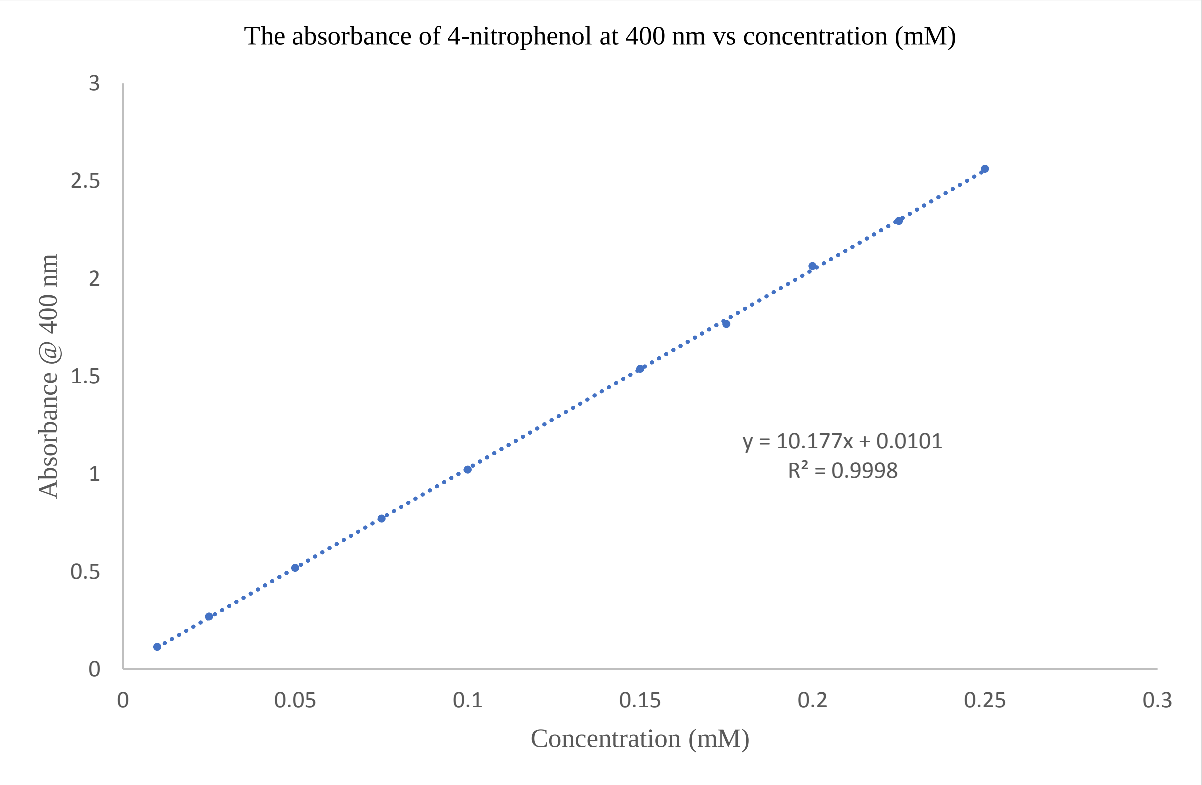

Supplementary Figure 5.3. Calibration curve of 4-nitrophenol.

B. Biological studies

Supplementary Results

Chemosensitivity studies results: The effect of anions on the potency and selectivity of all test compounds evaluated is presented in Supplementary Figures 6-8. The inclusion of anions either increased, decreased or had no effect on both potency (Supplementary Fig. 6) and the selectivity (Supplementary Fig. 7) of [$\mathbf{L}_2Cu_3$]$^{6+}$ and [$\mathbf{L}_2Zn_3$]$^{6+}$. Enhanced or reduced effects on potency and selectivity relative to [$\mathbf{L}_2Cu_3$]$^{6+}$ and [$\mathbf{L}_2Zn_3$]$^{6+}$ was strongly cell line dependent (Supplementary Fig. 8). Particularly marked enhancement of both potency and selectivity (relative to ARPE-19) was observed for PSN1 and HCT116 p53$^{+/+}$ cells treated with Cu^{2+} and Zn^{2+} ligands plus sulfate and phenyl phosphate anions. In contrast, the potency of the Cu^{2+} ligand plus the phenyl phosphate anion against BxPC3 cells is significantly reduced resulting in a reduction in selectivity compared to ARPE-19 cells. Different effects on non-cancer cell lines were also observed with only marginal effects of anion inclusion observed for the Cu^{2+} ligand (against both ARPE-19 and MCF10A cells) whereas for the Zn^{2+} ligand, its activity in the presence of all the anions tested was significantly reduced resulting in a relative loss of selectivity (Supplementary Fig. 8). The mechanistic reasons for these differential effects require further investigation but it suggests that the activity and selectivity of complexes can be tailored to individual cell lines, presumably via modulation of specific kinase inhibition activity and cell line dependent susceptibility to subsequent effects on the kinome. The ligand (**L**) and the mononuclear complex ([**L**M]$^{2+}$) are cytotoxic to cells *in vitro* but these species lack the selectivity exhibited by [$\mathbf{L}_2M_3$]$^{6+}$ (Supplementary Fig. 9)

Inhibition of kinase activity: The effect of [$\mathbf{L}_2Cu_3$]$^{6+}$ and [$\mathbf{L}_2Zn_3$]$^{6+}$on the activity of 140 human recombinant kinases is presented in Supplementary Fig. 10. Both compounds are multi-kinase inhibitors and visually, there are clear differences between the kinases that are inhibited by [$\mathbf{L}_2Cu_3$]$^{6+}$ and [$\mathbf{L}_2Zn_3$]$^{6+}$with more [$\mathbf{L}_2Cu_3$]$^{6+}$ inhibiting more kinases than [$\mathbf{L}_2Zn_3$]$^{6+}$. The differences that exist indicate that a different spectrum of inhibitory activity exists with evidence of selectivity. Of interest, are the results demonstrating that the activity of some kinases is enhanced following drug treatment, particularly Src where significant stimulation of kinase activity was observed in these cell free assays.

Phosphatase assay results: The effects of [$\mathbf{L}_2Zn_3$]$^{6+}$ concentration and duration of [$\mathbf{L}_2Zn_3$]$^{6+}$ exposure on dephosphorylation of recombinant AMPK (pT172 AMPKα1 and pS108 AMPKβ1) are presented in Supplementary Figure 11. For analysis of effects on pT172 AMPKα1, purified human $\alpha1\beta2\gamma1$ AMPK kinase was used. For analysis of effects on pS108 of AMPKβ1, purified human $\alpha2\beta1\gamma1$ AMPK kinase was used. Where total levels of the AMPK subunit decreased in response to incubation with [$\mathbf{L}_2Zn_3$]$^{6+}$, we hypothesise that this could be due to either some precipitation of the kinase by the [$\mathbf{L}_2Zn_3$]$^{6+}$complex or a consequence of dephosphorylation of the kinase by [$\mathbf{L}_2Zn_3$]$^{6+}$ resulting in its destabilisation and increased susceptibility to degradation. Dependency on ATP for dephosphorylation and the impact of AMP were also studied and are presented in Figure S12. Effects of [$\mathbf{L}_2Zn_3$]$^{6+}$ on p-T172 appeared to be dependent on the presence of ATP or AMP although observed effects were quite modest (Supplementary Fig. 12). AMP binding to the regulatory γ AMPK subunit is known to help protect p-T172 from physiological phosphatases.[5] In an AMPK kinase activity assay (Promega), however, increasing AMP concentration (0-160μM) had no adverse effect on the percentage inhibition of AMPK kinase activity by [$\mathbf{L}_2Zn_3$]$^{6+}$ (Supplementary Fig. 12c).

Autophagy studies: The effects of 3.125μM [$\mathbf{L}_2Cu_3$]$^{6+}$, [$\mathbf{L}_2Zn_3$]$^{6+}$ and [$\mathbf{L}_2Mn_3$]$^{6+}$ treatments (40h) on the induction of cellular vacuoles and autophagy in the HCT116 p53$^{-/-}$, HCT116 p53$^{+/+}$ and ARPE19 cells are presented in Supplementary Figures 13-15 with representative cell images shown. At this concentration, induction of autophagy was most pronounced in the HCT116 p53$^{-/-}$cells with [$\mathbf{L}_2Mn_3$]$^{6+}$ treatment followed by [$\mathbf{L}_2Zn_3$]$^{6+}$ with no or little autophagy induced by [$\mathbf{L}_2Cu_3$]$^{6+}$ compared to vehicle control treated cells (Supplementary Fig. 11). In the HCT116 p53$^{+/+}$ cells, induction of autophagy was

also evident in the [$\mathbf{L}_2Mn_3$]$^{6+}$ treated cells but was barely detectable in the [$\mathbf{L}_2Zn_3$]$^{6+}$ treated cells at this concentration compared to in the HCT116 p53$^{-/-}$ cells (Supplementary Fig. 12). This is consistent with [$\mathbf{L}_2Zn_3$]$^{6+}$ being ~6-fold more active towards the HCT116 p53$^{-/-}$ cells than the HCT116 p53$^{+/+}$ cells based on 96h IC_{50} values. In the ARPE19 cells, levels of autophagy were low with all treatments and similar to basal levels in control-treated cells (Supplementary Fig. 13).

Cellular uptake of [$\mathbf{L_2Zn_3}$]$^{6+}$: Following the treatment of chemosensitive HCT116 p53$^{+/+}$ and chemoresistant ARPE-19 cells with 25μM [$\mathbf{L}_2Zn_3$]$^{6+}$ for 1 hour, an increase in TSQ fluorescent signal intensity above background (TSQ treatment only) was observed in both cell lines (Supplementary Fig. 16). These results suggest that (i) the complex is able to enter cells and (ii) the differential response of the cells (>300 fold difference in IC_{50} values) is unlikely to be due to differential uptake but reflects differences in drug/target interactions and downstream effects in each cell line. Whilst further studies would be required to validate these findings in other cell lines and with other complexes, these results suggest that differential drug uptake is not a major factor that determines differential chemosensitivity *in vitro*.

Cellular ATP studies: The effects of 20h treatment with a range of different concentrations of [$\mathbf{L}_2Cu_3$]$^{6+}$, [$\mathbf{L}_2Zn_3$]$^{6+}$ and [$\mathbf{L}_2Mn_3$]$^{6+}$ on total cellular ATP levels in HCT116 p53$^{+/+}$ and ARPE19 cells are presented in Supplementary Fig. 17. Differential effects are observed depending on the metal in the complex, with [$\mathbf{L}_2Mn_3$]$^{6+}$ having the most profound effects on both the HCT116 p53$^{+/+}$ cancer and ARPE19 non-cancer cells. 6.25μM [$\mathbf{L}_2Mn_3$]$^{6+}$ treatment for 20h reduced ATP levels in the HCT116 p53$^{+/+}$ cancer cells to <50% with ATP levels declining less in the ARPE19 non-cancer cells but reaching ~40% with 100μM [$\mathbf{L}_2Mn_3$]$^{6+}$ treatment. [$\mathbf{L}_2Zn_3$]$^{6+}$ also reduced ATP levels in the HCT116 p53$^{+/+}$ cancer cells with levels reduced to <50% at concentrations of ≥25μM [$\mathbf{L}_2Zn_3$]$^{6+}$ whereas in the ARPE19 cells ATP levels remained >70% with 100μM [$\mathbf{L}_2Zn_3$]^{6} treatment. Effects of [$\mathbf{L}_2Cu_3$]$^{6+}$ on ATP levels was much less, with ATP levels in the HCT116 cells reduced to <50% only at the highest concentration of 100μM.

LDH-A phosphorylation at tyrosine 10: Given the phosphatase activity of [$\mathbf{L}_2Zn_3$]$^{6+}$ towards specific kinases and also the observed inhibitory effects of [$\mathbf{L}_2Zn_3$]$^{6+}$ on glycolysis in the HCT116 cells, effects of [$\mathbf{L}_2Zn_3$]$^{6+}$ on phosphorylated Y10 levels of LDH-A were analysed as a potential phospho-substrate of [$\mathbf{L}_2Zn_3$]$^{6+}$. Phospho-Y10 levels decreased modestly in the ARPE19 cells relative to total LDH-A levels and to a lesser extent in the HCT116 cells (Supplementary Fig. 18).

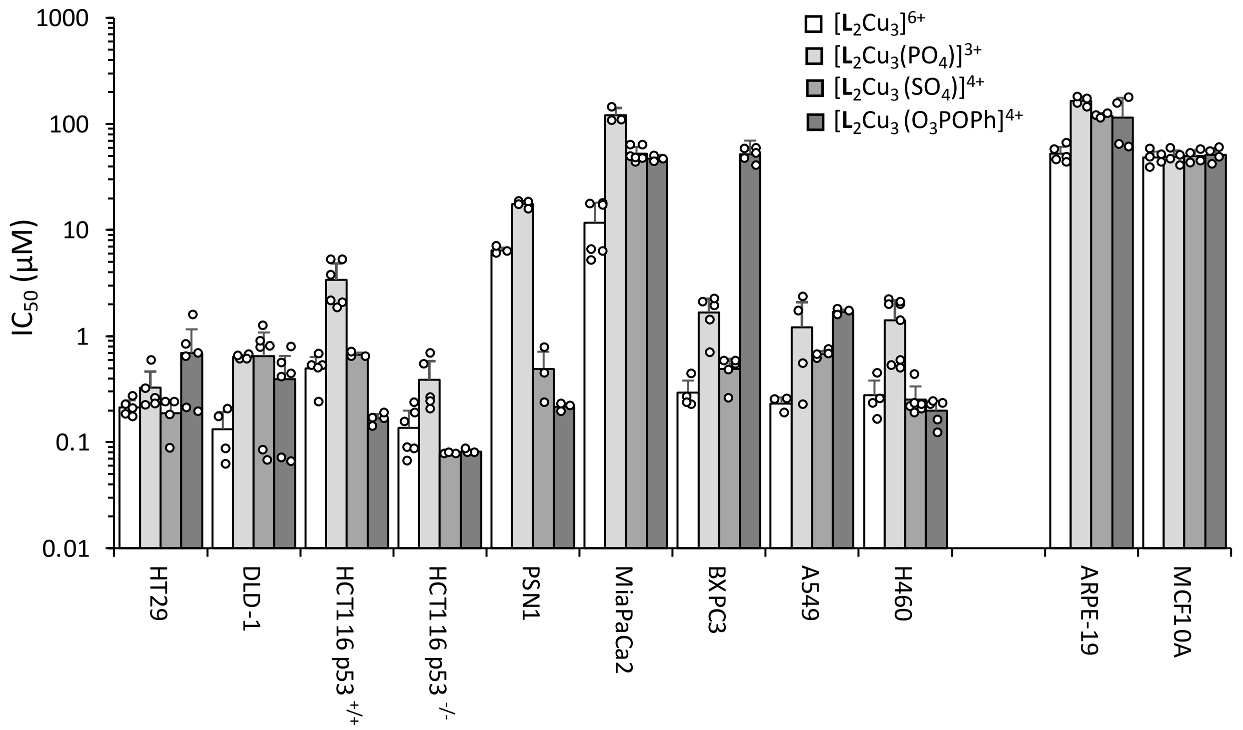

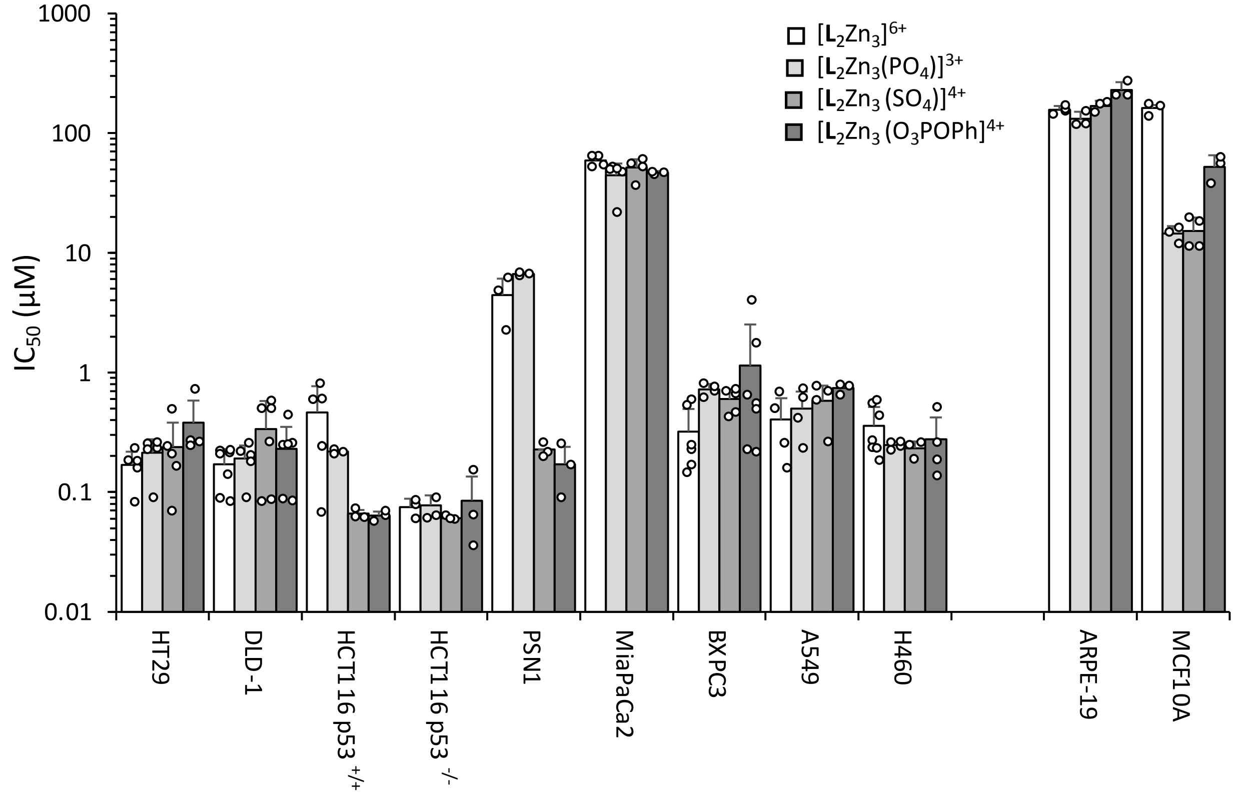

Supplementary Figure 6. The effect of anions of the potency of $[L_2Cu_3]^{6+}$ and $[L_2Zn_3]^{6+}$. The results represent the mean IC_{50} values ± standard deviation for at least three independent experiments.

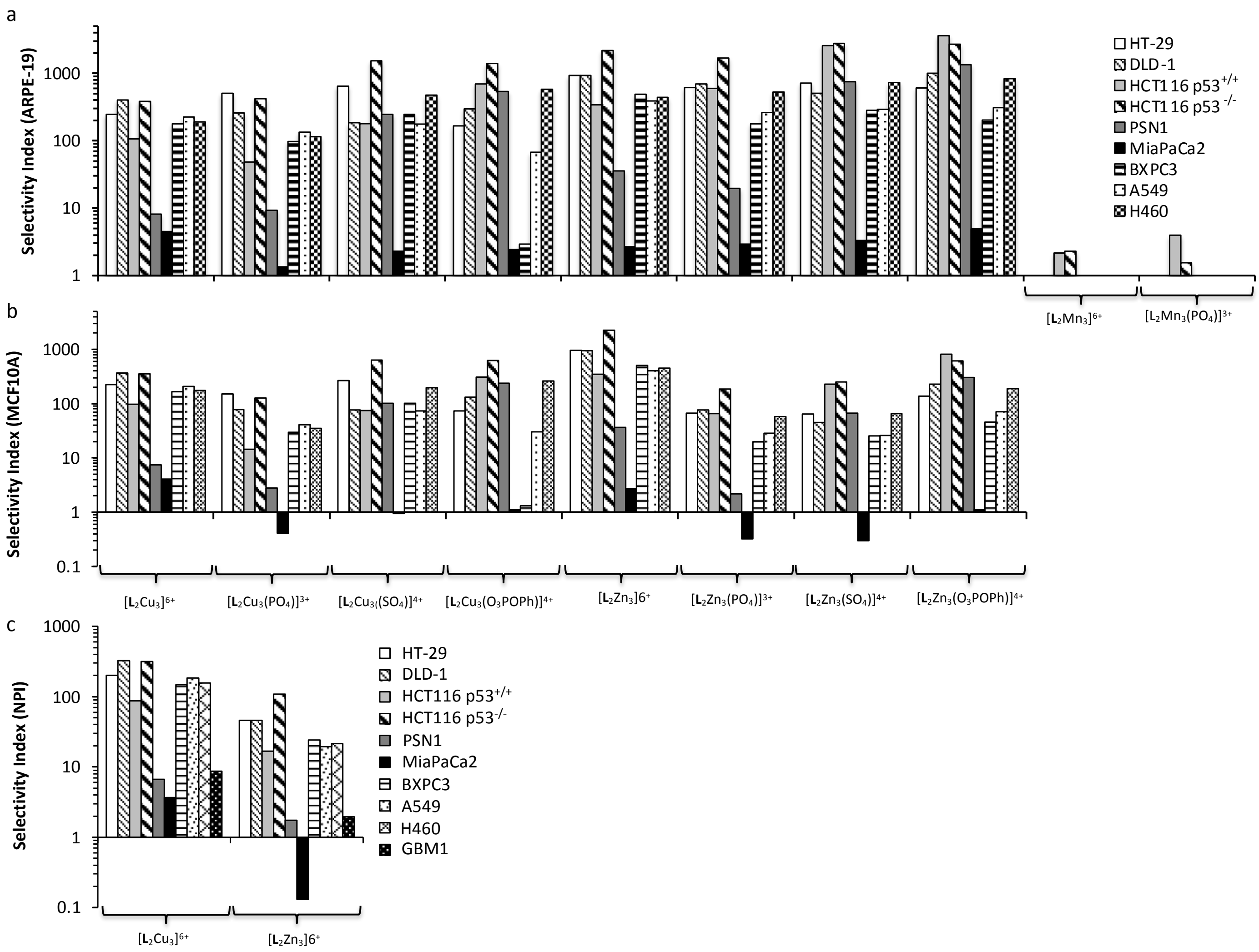

Supplementary Figure 7. The effect of anions on the selectivity of $[L_2Cu_3]^{6+}$ and $[L_2Zn_3]^{6+}$. All values presented here were determined from the mean IC_{50} values in Fig. S6 for each of the non-cancer cell lines (ARPE-19, MCF10A and NP1) used in this study. As mean IC_{50} values are used to calculate SI, no error bars are presented. The experimental error is accounted for in Supplementary Figure 6.

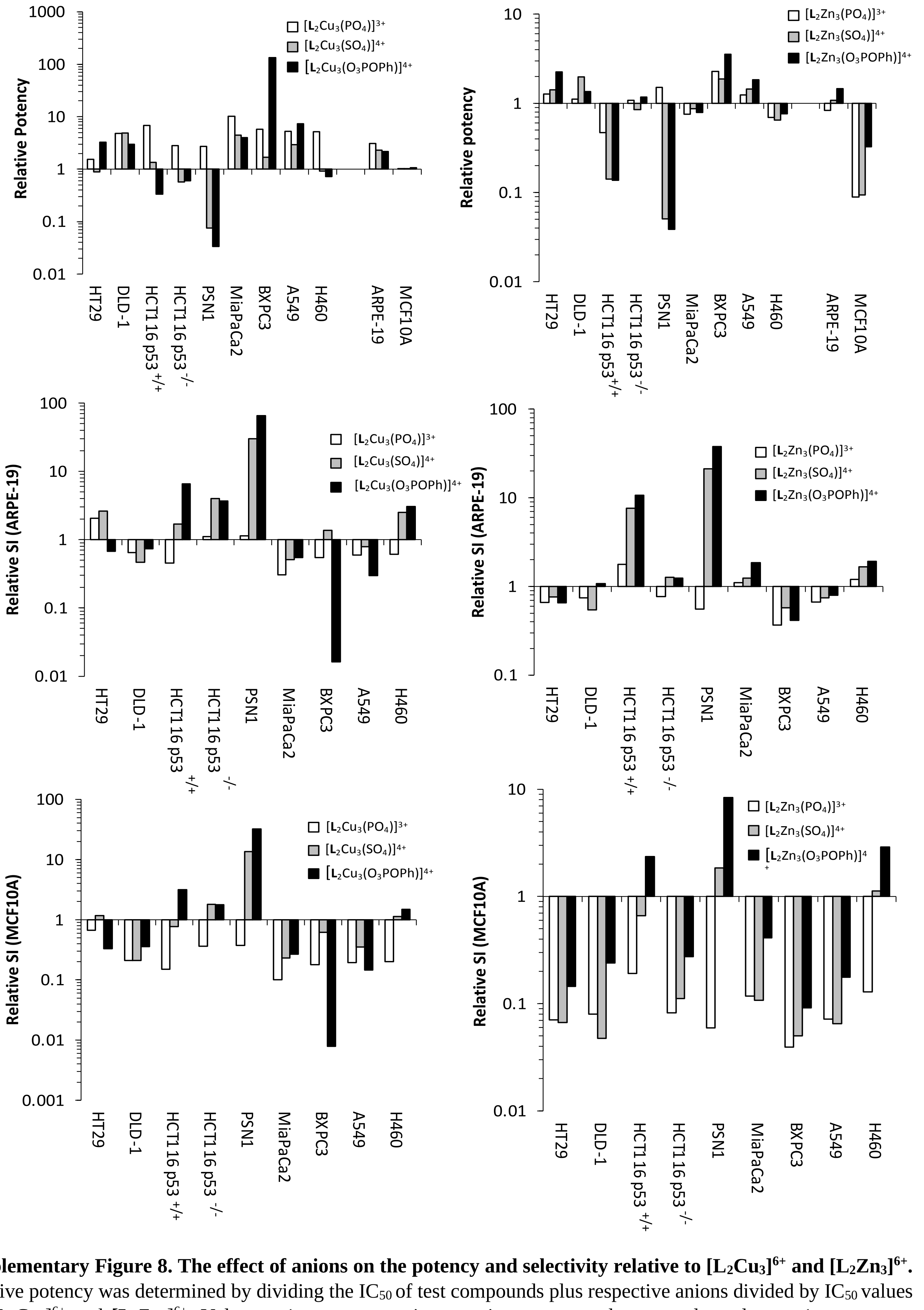

Supplementary Figure 8. The effect of anions on the potency and selectivity relative to [L_2Cu_3]$^{6+}$ and [L_2Zn_3]$^{6+}$. Relative potency was determined by dividing the IC_{50} of test compounds plus respective anions divided by IC_{50} values for [**L**$_2$Cu$_3$]$^{6+}$ and [**L**$_2$Zn$_3$]$^{6+}$. Values > 1 represent an increase in potency and conversely, values < 1 represent a reduction in potency. Relative selectivity index (SI) values were determined by dividing the SI value for test compounds plus respective anions divided by SI values for [**L**$_2$Cu$_3$]$^{6+}$ and [**L**$_2$Zn$_3$]$^{6+}$. Values > 1 represent an increase in selectivity and conversely, values < 1 represent a reduction in selectivity.

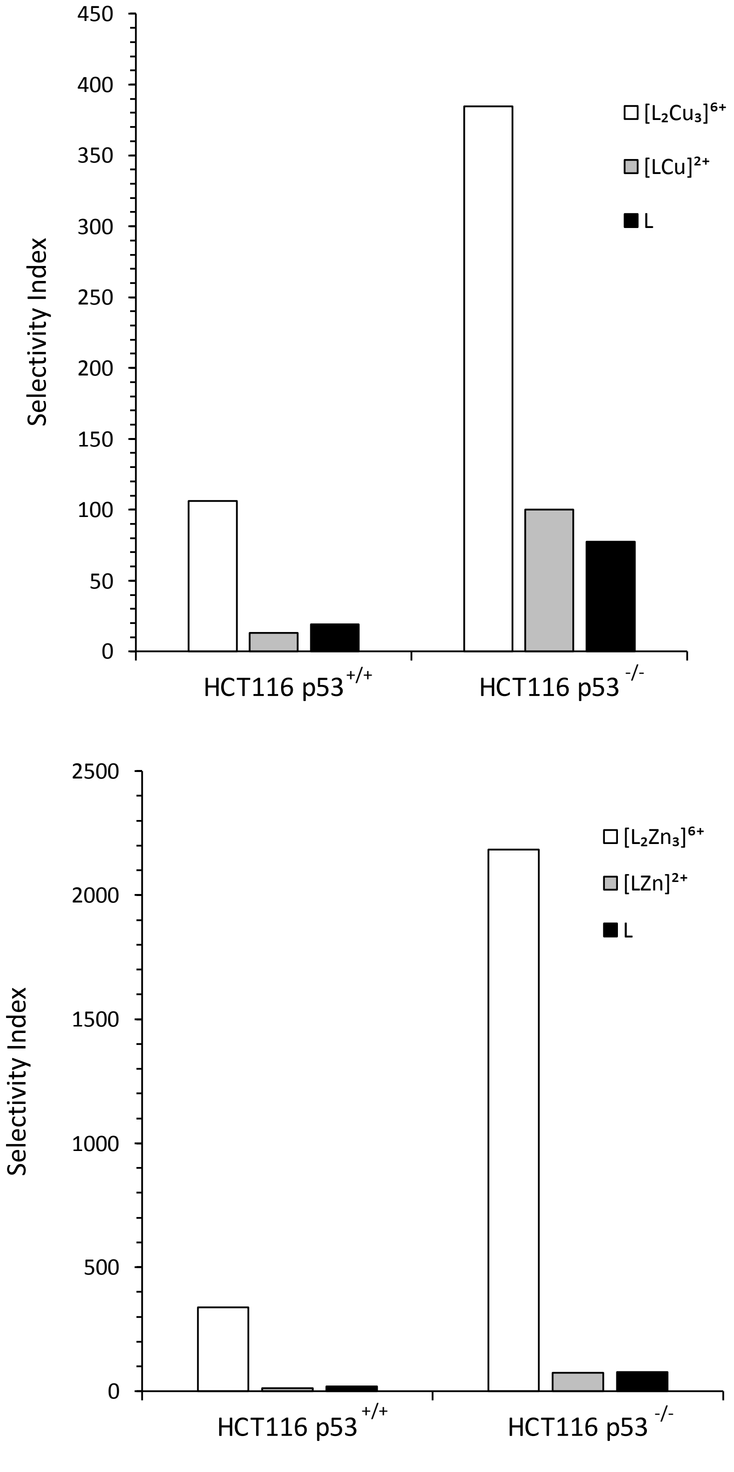

Supplementary Figure 9. Comparative selectivity of $[L_2M_3]^{6+}$ to $[LM]^{2+}$ and L. The selectivity index (SI) values were determined by dividing the mean IC_{50} of each test compound against ARPE-19 cells by the mean IC_{50} for HCT116 $p53^{+/+}$ and $p53^{-/-}$ cells. The results demonstrate that whilst $[\mathbf{L}M]^{2+}$ and L are cytotoxic to cells, selectivity for cancer cells is property confined primarily to the $[\mathbf{L}_2M_3]^{6+}$ complexes. $[\mathbf{L}M]^{2+}$ refers to an experiment where stoichiometric amounts of ligand and metal ions are used (as opposed to 2**L** plus $3M^{2+}$) thereby forcing the formation of $[LM]^{2+}$. In all likelihood, in the presence of tetrahedral oxoanions, this would result in forming $[\mathbf{L}_2M_3(EO_4)]^{3/4+}$ (where E = P or S) and free ligand.

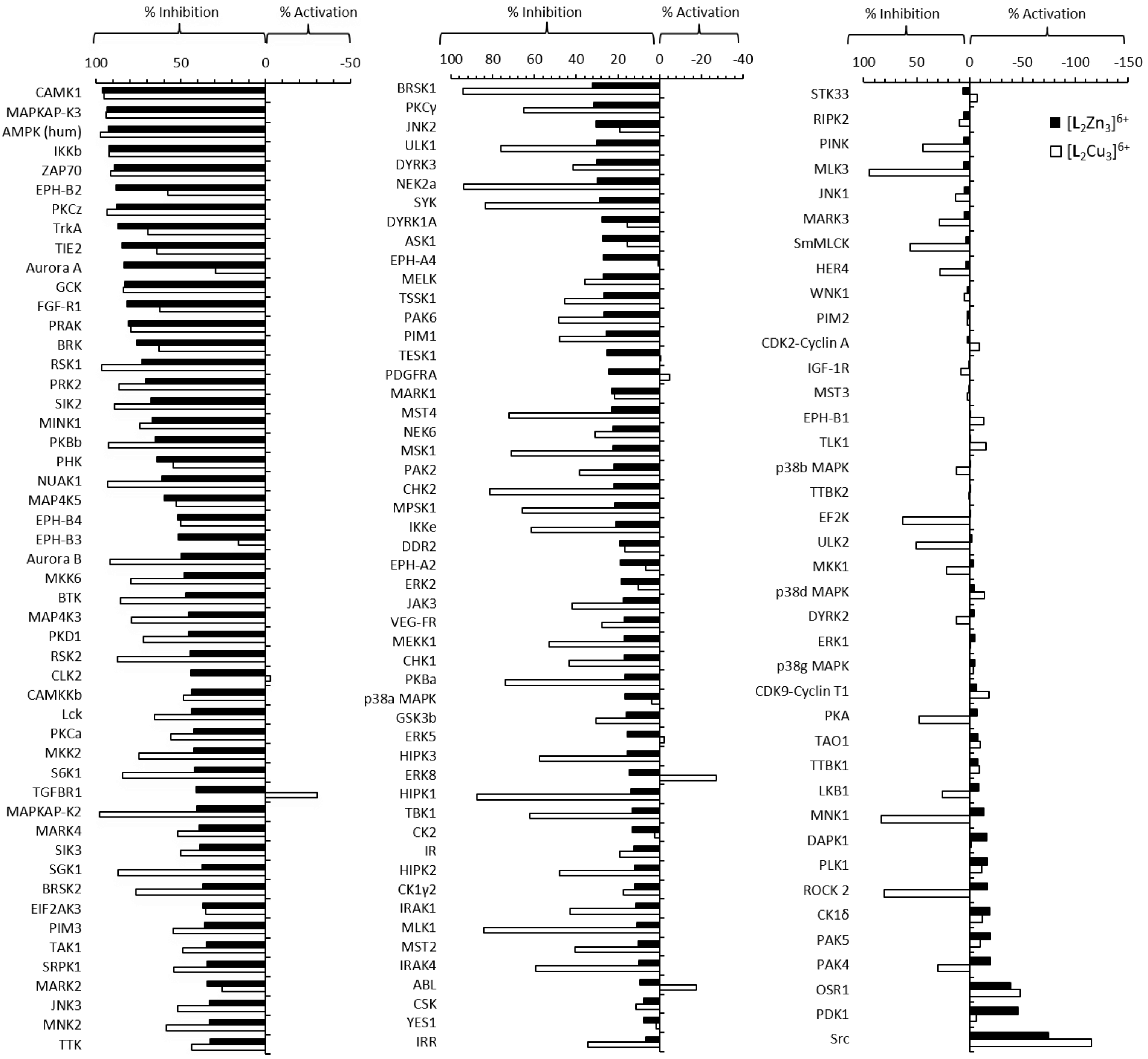

Supplementary Figure 10. The effect of $[L_2Zn_3]^{6+}$ and $[L_2Cu_3]^{6+}$ on the activity of recombinant human kinases. The compounds were submitted to the MRC Protein Phosphorylation and Ubiquitination Unit International Centre for Kinase Profiling (University of Dundee) and tested at a concentration of 10 μM against 140 human kinases (Premier Screen).

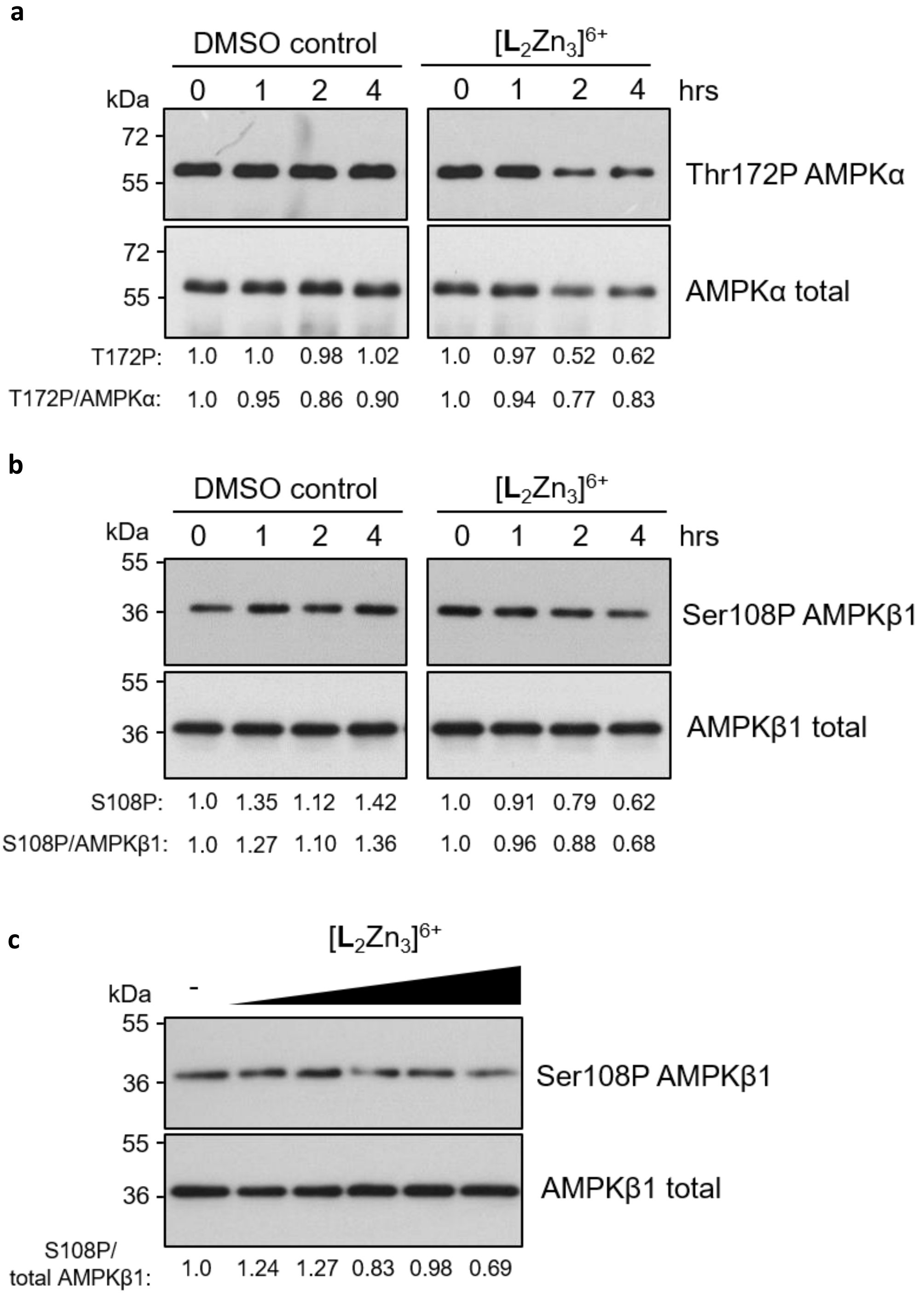

Supplementary Figure 11. Effects of $[\mathbf{L}_2Zn_3]^{6+}$ on phospho-T172 levels of recombinant AMPKα1 and phospho-S108 levels of recombinant AMPKβ1. a, b Time course of 50µM $[\mathbf{L}_2Zn_3]^{6+}$ co-incubation with recombinant AMPK enzyme (α1β2γ1 for (**a**) and α2β1γ1 for (**b**)) and effects on phosphorylation levels of T172 of AMPKα1 (**a**) and S108 of AMPKβ1 (**b**). Densitometric quantification of levels of phosphorylated AMPK subunit and relative to total levels of the subunit are indicated; all quantification relative to t=0. **c,** Effect of increasing concentrations of $[\mathbf{L}_2Zn_3]^{6+}$ (0-50µM, 2-fold dilution series) on phospho-S108 AMPK β1 relative to total AMPK β1. Similar results were obtained in a minimum of n=2 independent experiments.

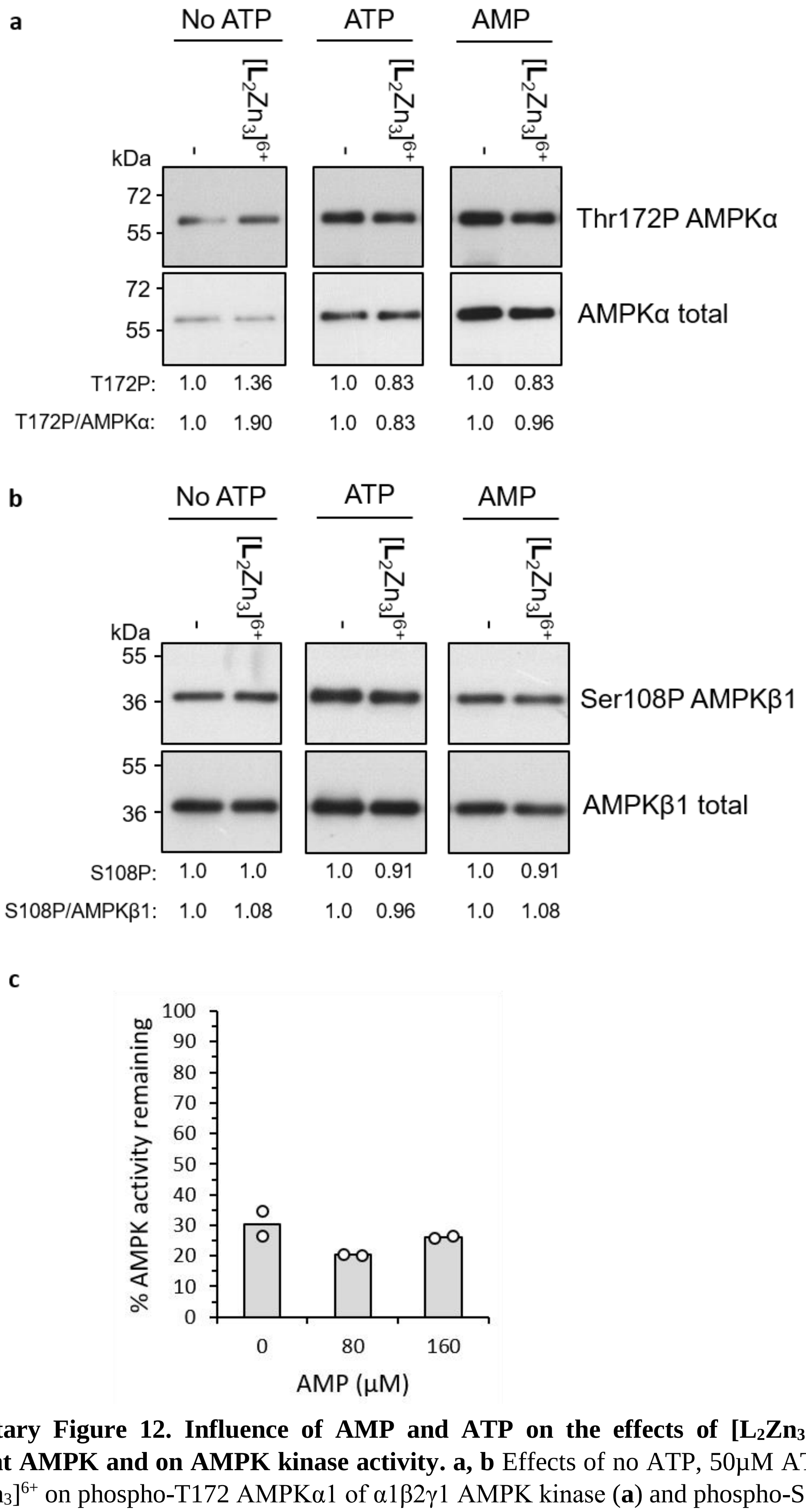

Supplementary Figure 12. Influence of AMP and ATP on the effects of $[\mathbf{L}_2Zn_3]^{6+}$ on phosphorylation levels of recombinant AMPK and on AMPK kinase activity. a, b Effects of no ATP, 50µM ATP or 50µM AMP on the effects of 50µM $[\mathbf{L}_2Zn_3]^{6+}$ on phospho-T172 AMPKα1 of α1β2γ1 AMPK kinase (**a**) and phospho-S108 of AMPKβ1 of α2β1γ1 AMPK kinase (**b**). Densitometric quantification of levels of phosphorylated AMPK subunit and relative to total levels of the subunit are indicated; all quantification relative to solvent control. For **a** and **b**, similar results were obtained in a minimum of n=2 independent experiments. **c,** Effect of increasing concentrations of AMP (0, 80, 160µM) on AMPK kinase inhibition by $[\mathbf{L}_2Zn_3]^{6+}$. % AMPK activity is relative to activity in the vehicle control ± AMP (0, 80 or 160µM AMP). n=2 independent experiments, with corresponding data points as indicated.

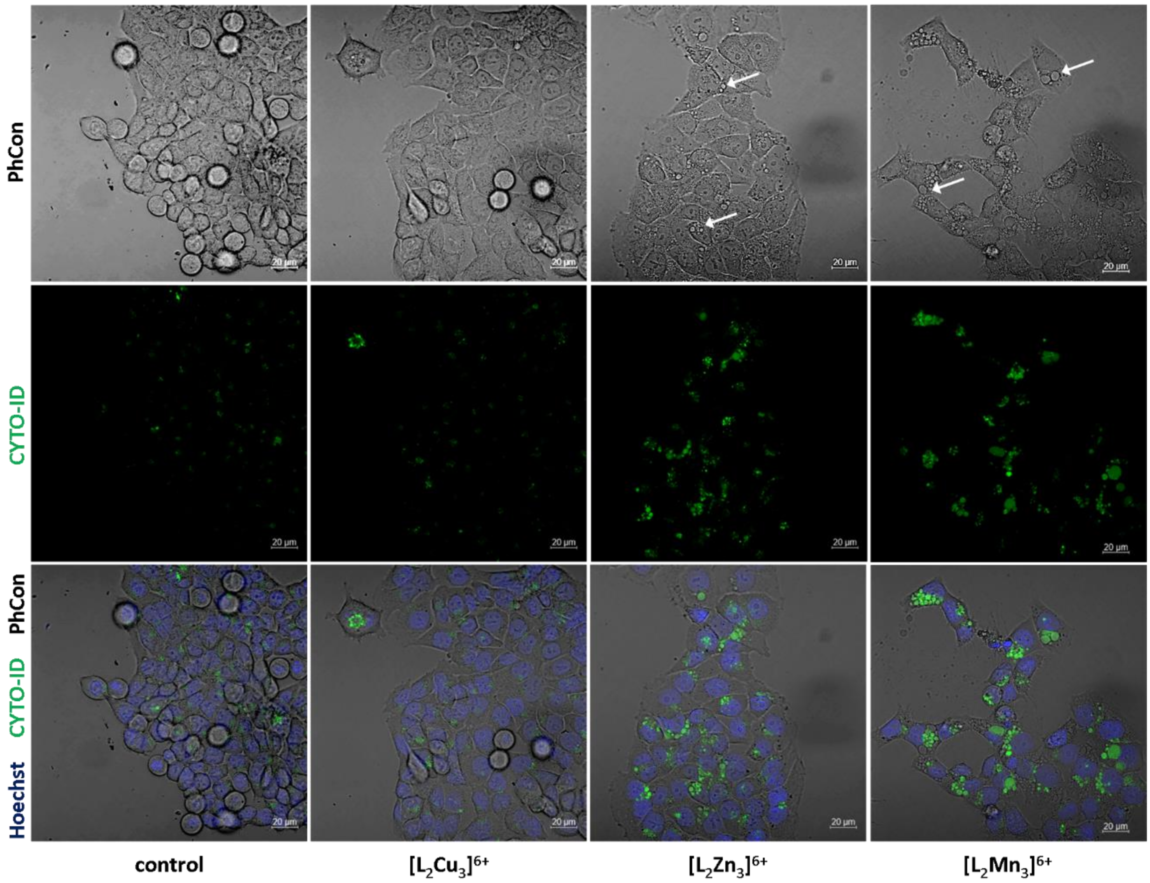

Supplementary Figure 13. The effects of [L_2Cu_3]$^{6+}$, [L_2Zn_3]$^{6+}$ and [L_2Mn_3]$^{6+}$ on cellular vacuole formation and autophagy in the HCT116 p53$^{-/-}$ cancer cells. Representative confocal images are shown of HCT116 p53$^{-/-}$ cancer cells following 40h treatment with vehicle control or 3.125µM [**L**$_2$Cu$_3$]$^{6+}$, [**L**$_2$Zn$_3$]$^{6+}$, or [**L**$_2$Mn$_3$]$^{6+}$. Upper panel shows phase contrast cell images with white arrows indicating intracellular vacuoles. Middle panel shows cells staining positive for autophagy (CYTO-ID autophagic dye, green). Lower panel shows overlay of phase contrast cell images with CYTO-ID autophagic staining (green, punctate, cytoplasmic) and counterstaining of nuclei (Hoechst). Similar results were obtained in n=3 independent experiments.

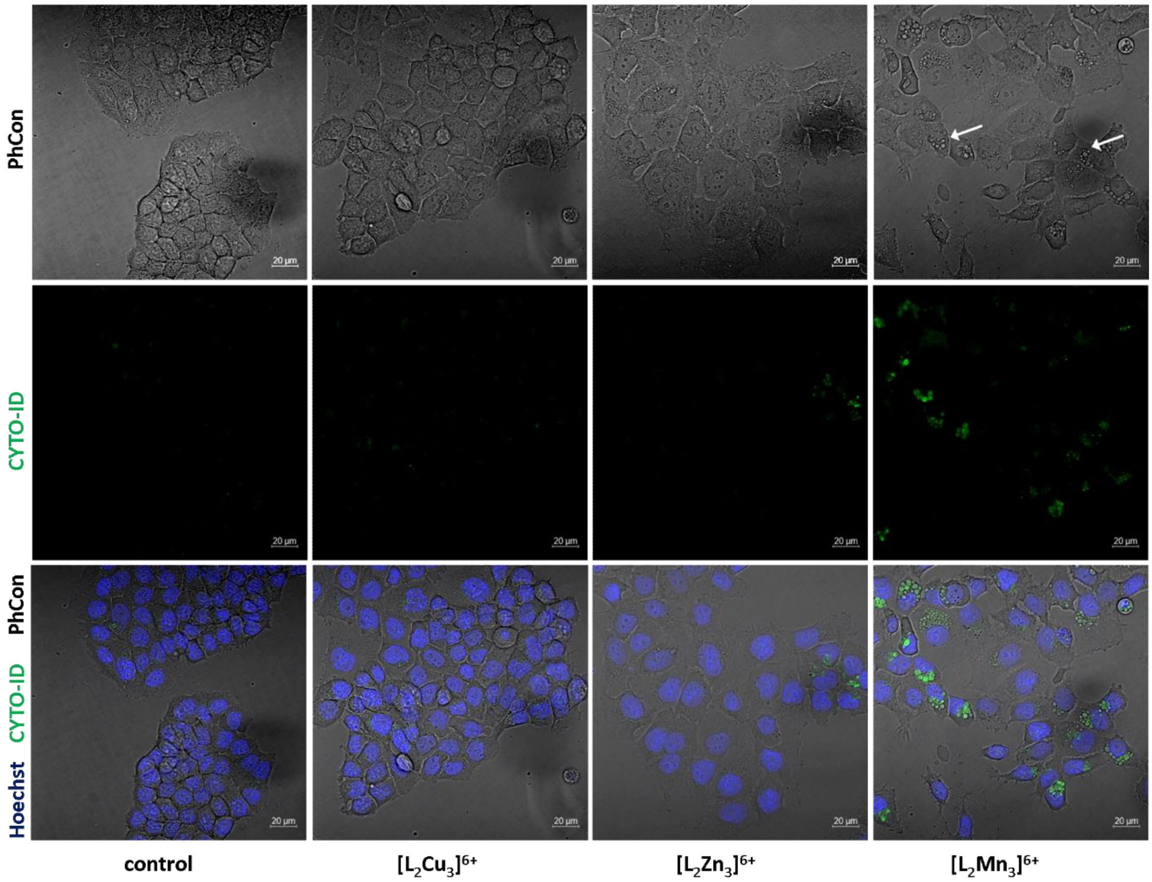

Supplementary Figure 14. The effects of $[L_2Cu_3]^{6+}$, $[L_2Zn_3]^{6+}$ and $[L_2Mn_3]^{6+}$ on cellular vacuole formation and autophagy in the HCT116 p53$^{+/+}$ cancer cells.

Representative confocal images are shown of HCT116 p53$^{+/+}$ cancer cells following 40h treatment with vehicle control or 3.125μM $[\mathbf{L}_2Cu_3]^{6+}$, $[\mathbf{L}_2Zn_3]^{6+}$, or $[\mathbf{L}_2Mn_3]^{6+}$. Upper panel shows phase contrast cell images with white arrows indicating intracellular vacuoles. Middle panel shows cells staining positive for autophagy (CYTO-ID autophagic dye, green). Lower panel shows overlay of phase contrast cell images with CYTO-ID autophagic staining (green, punctate, cytoplasmic) and counterstaining of nuclei (Hoechst). Similar results were obtained in n=3 independent experiments.

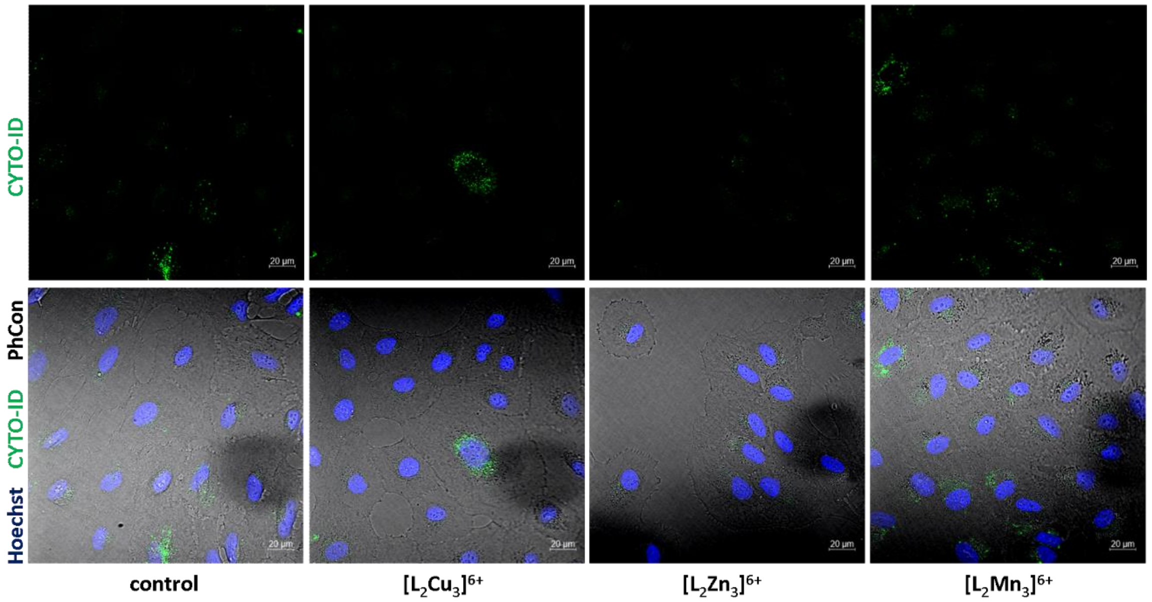

Supplementary Figure 15. The effects of $[L_2Cu_3]^{6+}$, $[L_2Zn_3]^{6+}$ and $[L_2Mn_3]^{6+}$ on cellular vacuole formation and autophagy in the ARPE19 non-cancer cells.

Representative confocal images are shown of ARPE19 non-cancer cells following 40h treatment with vehicle control or 3.125µM $[\mathbf{L}_2Cu_3]^{6+}$, $[\mathbf{L}_2Zn_3]^{6+}$, or $[\mathbf{L}_2Mn_3]^{6+}$. Upper panel shows cells staining positive for autophagy (CYTO-ID autophagic dye, green). Lower panel shows overlay of phase contrast cell images with CYTO-ID autophagic staining (green, punctate, cytoplasmic) and counterstaining of nuclei (Hoechst). Similar results were obtained in n=3 independent experiments.

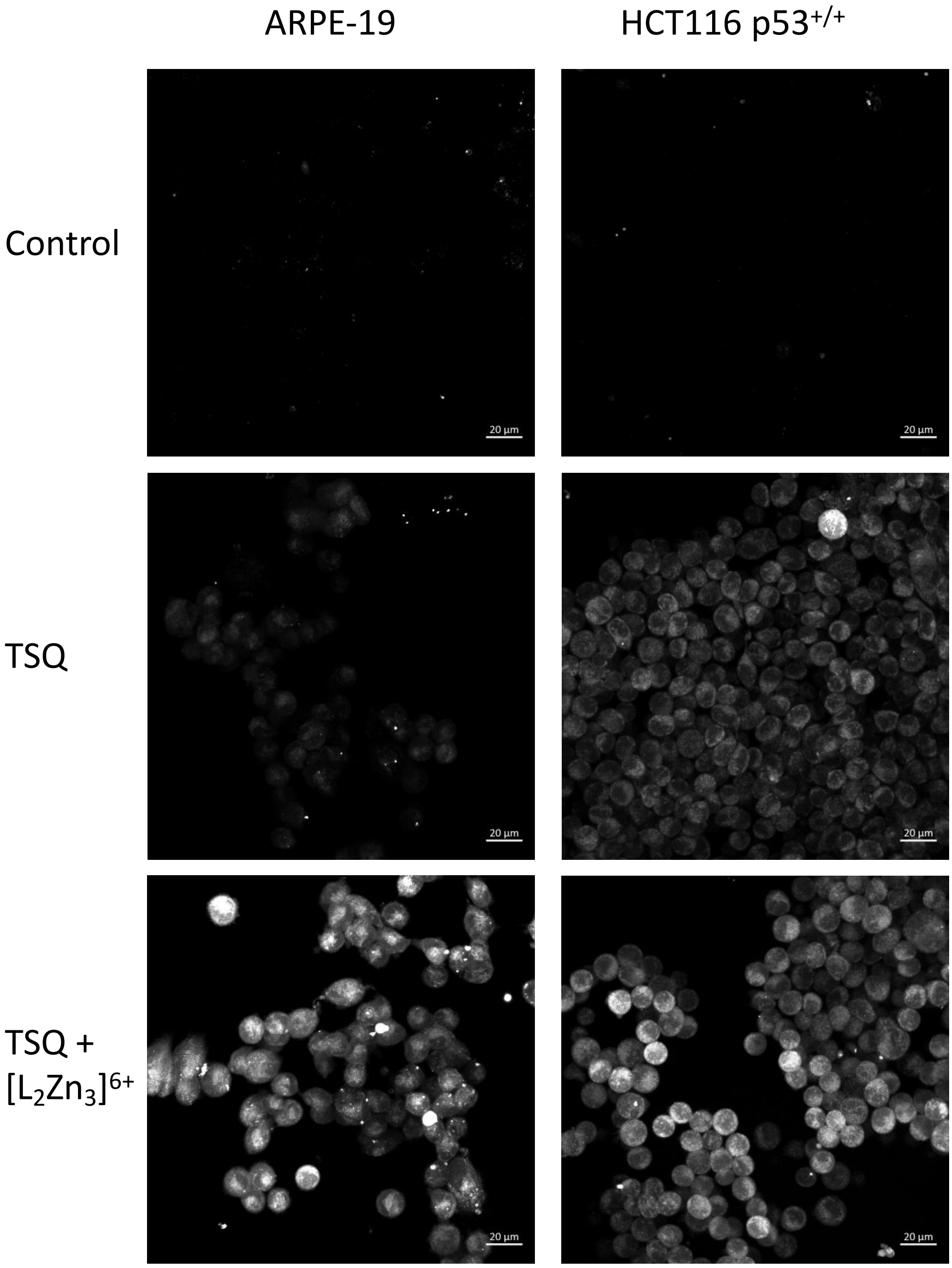

Supplementary Figure 16. Uptake of [L_2Zn_3]$^{6+}$ in the chemosensitive HCT116 p53$^{+/+}$ and chemoresistant ARPE-19 cell lines. Representative confocal images of cells treated without TSQ (control), with TSQ only and with TSQ + [**L**$_2$Zn$_3$]$^{6+}$ (25 μM for 1 hour). TSQ only treatment records background levels of Zn in cells. Similar results were obtained in n=2 independent experiments.

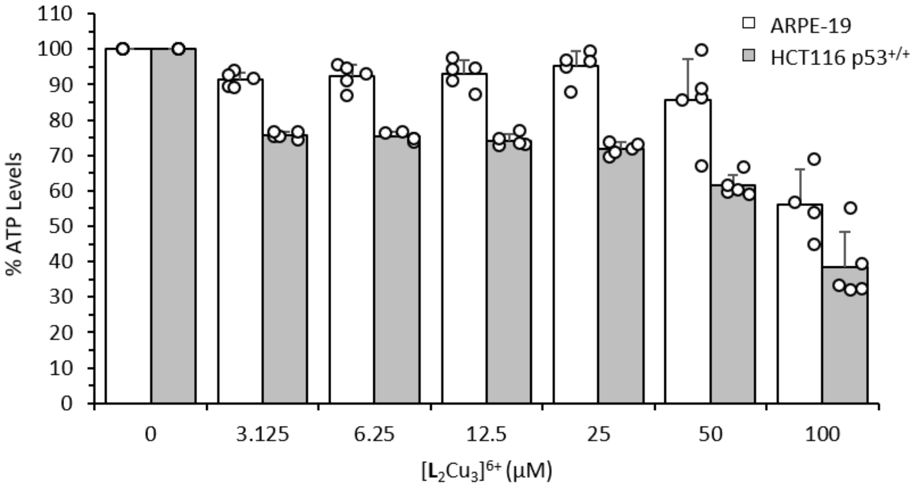

[L_2Cu_3]$^{6+}$ (μM)	P values (ARPE19 vs. HCT116)
0	N/A
3.125	$2.58x10^{-7}$
6.25	$6.72x10^{-6}$
12.5	$1.00x10^{-5}$
25	$3.76x10^{-6}$
50	$2.30x10^{-3}$
100	$3.28x10^{-2}$

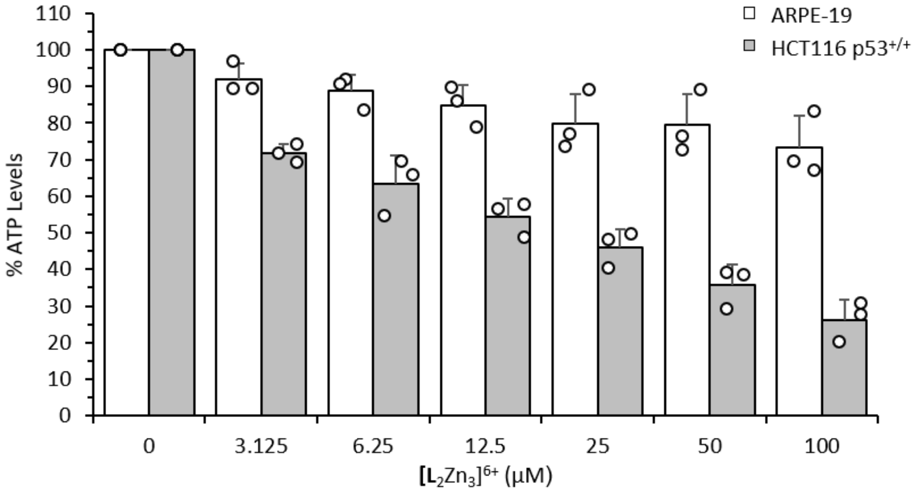

[L_2Zn_3]$^{6+}$ (μM)	P values (ARPE19 vs. HCT116)
0	N/A
3.125	$2.05x10^{-3}$
6.25	$8.04x10^{-3}$
12.5	$2.03x10^{-3}$
25	$3.54x10^{-3}$
50	$1.76x10^{-3}$
100	$1.37x10^{-3}$

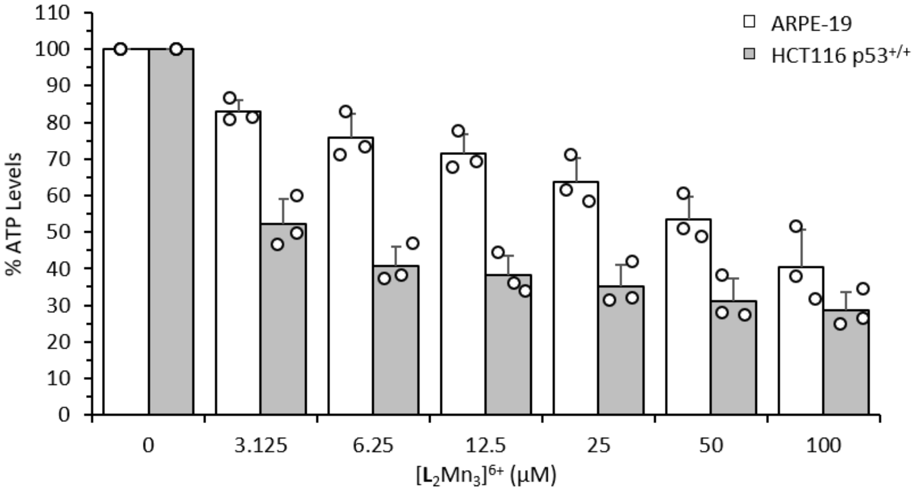

[L_2Mn_3]$^{6+}$ (μM)	P values (ARPE19 vs. HCT116)
0	N/A
3.125	$2.21x10^{-3}$
6.25	$1.85x10^{-3}$
12.5	$1.57x10^{-3}$
25	$4.86x10^{-3}$
50	$1.15x10^{-2}$
100	$1.46x10^{-1}$

Supplementary Figure 17. The effects of [L_2Cu_3]$^{6+}$, [L_2Zn_3]$^{6+}$ and [L_2Mn_3]$^{6+}$ on cellular ATP levels in the ARPE19 non-cancer and HCT116 p53$^{+/+}$ cancer cells. The effects of 20h treatment with the indicated concentrations of [$\mathbf{L}_2Cu_3$]$^{6+}$, [$\mathbf{L}_2Zn_3$]$^{6+}$, or [$\mathbf{L}_2Mn_3$]$^{6+}$ on total cellular levels of ATP compared to levels in vehicle control-treated ARPE-19 or HCT116$^{+/+}$ cells. Results are presented as the mean ± SD for a minimum of 3 biological repeats; two-sided students t-test (p values) to test for statistical significance between ARPE19 and HCT116 p53$^{+/+}$ cells as indicated.

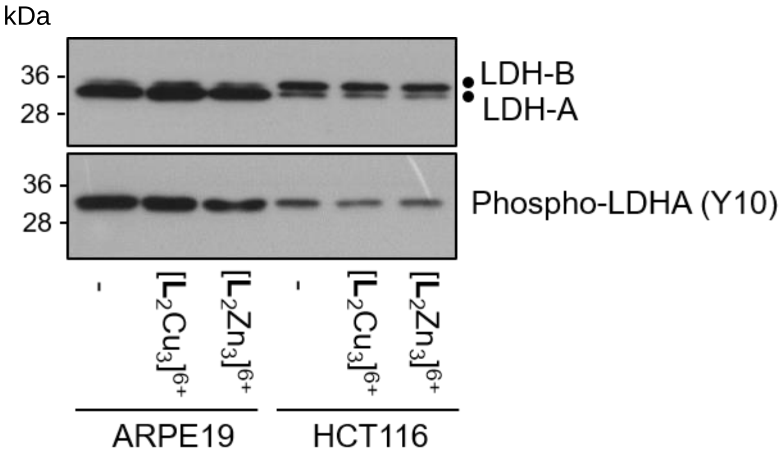

Supplementary Figure 18. Immunoblots showing the effects of $[L_2Zn_3]^{6+}$ and $[L_2Cu_3]^{6+}$ on phospho-Y10 of lactate dehydrogenase A. ARPE19 non-cancer cells and HCT116 cancer cells were treated with 10μM $[\mathbf{L}_2Cu_3]^{6+}$ or $[\mathbf{L}_2Zn_3]^{6+}$ for 4h prior to harvesting and immunoblot analysis for total levels of LDH-A and LDH-B and phosphorylated levels of tyrosine 10 of LDH-A. This experiment has been independently performed n=1.

Supplementary References

1. Rice, C. R., Slater, C., Faulkner, R. A. & Allan, R. L. *Angew. Chem.* **57**, 13255-13259 (2018).
2. Sheldrick, G. M. SHELXTL, Version 6.12 (Bruker Analytical X-ray Systems, Inc., Madison, WI, 2000).
3. Bourhis, L.J., Dolomanov, O.V., Gildea, R.J., Howard, J.A.K. & Puschmann, H. The anatomy of a comprehensive constrained, restrained refinement program for the modern computing environment – Olex2 dissected. *Acta Cryst.* **A71**, 59-75 (2015).
4. Sheldrick, G. M. *SADABS:* A Program for Absorption Correction. University of Göttingen, Germany (1996).
5. Hardie, D.G., Ross F.A, Hawley, S.A. AMPK: a nutrient and energy sensor that maintains energy homeostasis. *Nat. Rev. Mol. Cell. Biol.* **13**, 251-262 (2012).
