# Supplementary material for: Self-assembly of an anion receptor with metal-dependent kinase inhibition and potent in vitro anti-cancer properties
Source: Nat Commun. 2021 Jun 23;12:3898. doi: 10.1038/s41467-021-23983-3 (PMC8222254; doi:10.1038/s41467-021-23983-3)
Supplement: Supplementary file 3 — Reporting Summary [file 41467_2021_23983_MOESM3_ESM.pdf]

nature research

Corresponding author(s): Rice, C.R

Last updated by author(s): 15/05/21

Reporting Summary

Nature Research wishes to improve the reproducibility of the work that we publish. This form provides structure for consistency and transparency in reporting. For further information on Nature Research policies, see our Editorial Policies and the Editorial Policy Checklist.

Statistics

For all statistical analyses, confirm that the following items are present in the figure legend, table legend, main text, or Methods section.

n/a	Confirmed	
☐	☒	The exact sample size (n) for each experimental group/condition, given as a discrete number and unit of measurement
☐	☒	A statement on whether measurements were taken from distinct samples or whether the same sample was measured repeatedly
☐	☒	The statistical test(s) used AND whether they are one- or two-sided<br>*Only common tests should be described solely by name; describe more complex techniques in the Methods section.*
☒	☐	A description of all covariates tested
☒	☐	A description of any assumptions or corrections, such as tests of normality and adjustment for multiple comparisons
☐	☒	A full description of the statistical parameters including central tendency (e.g. means) or other basic estimates (e.g. regression coefficient) AND variation (e.g. standard deviation) or associated estimates of uncertainty (e.g. confidence intervals)
☐	☒	For null hypothesis testing, the test statistic (e.g. F, t, r) with confidence intervals, effect sizes, degrees of freedom and P value noted<br>*Give P values as exact values whenever suitable.*
☒	☐	For Bayesian analysis, information on the choice of priors and Markov chain Monte Carlo settings
☒	☐	For hierarchical and complex designs, identification of the appropriate level for tests and full reporting of outcomes
☒	☐	Estimates of effect sizes (e.g. Cohen's d, Pearson's r), indicating how they were calculated

Our web collection on statistics for biologists contains articles on many of the points above.

Software and code

Policy information about availability of computer code

Data collection	Computer code for crystallography: SHELXS-97 (for solution), SHELXL-2014 and Olex2 (for refinement) and SHELXL (for molecular graphics) software respectively.
Data analysis	Kinase gene IDs were translated into common IDs (HGNC symbol) using biomaRt package in R and R Studio and used as input to the KinMap programme. The KinMap output was then manually annotated to include the top activated and inhibited kinases as shown in Figure 7e,f. Densitometric quantification of blots was performed using Image J (Fiji) software.

For manuscripts utilizing custom algorithms or software that are central to the research but not yet described in published literature, software must be made available to editors and reviewers. We strongly encourage code deposition in a community repository (e.g. GitHub). See the Nature Research guidelines for submitting code & software for further information.

Data

Policy information about availability of data

All manuscripts must include a data availability statement. This statement should provide the following information, where applicable:

- Accession codes, unique identifiers, or web links for publicly available datasets
- A list of figures that have associated raw data
- A description of any restrictions on data availability

The raw data that support the findings of this study are provided in the main article, supplementary information or Source data file, or are otherwise available from the corresponding author(s) upon reasonable request.

Field-specific reporting

Please select the one below that is the best fit for your research. If you are not sure, read the appropriate sections before making your selection.

- [x] Life sciences
- [ ] Behavioural & social sciences
- [ ] Ecological, evolutionary & environmental sciences

For a reference copy of the document with all sections, see nature.com/documents/nr-reporting-summary-flat.pdf

Life sciences study design

All studies must disclose on these points even when the disclosure is negative.

Sample size	For assessment of in vitro anti-cancer activity of the self-assembling compounds reported in this paper and any selectivity towards cancer cells compared to non-cancer cells, a panel of 10 cancer cell lines of different tissue origin or of different genetic backgrounds were tested and compared to three validated non-cancer cell models. This ensured that conclusions were not based on a single cell line and standard requirements for rigorous analysis were met.<br><br>Sample sizes are disclosed in the methods or legends as appropriate. For cell-based assays, sample sizes were chosen based on previous determinations of optimal seeding density for log phase growth and to ensure that control treated cells remained in log phase growth and did not reach full confluency and enter plateau stage by the assay endpoint (e.g. for chemosensitivity testing, a linear relationship was maintained between endpoint absorbance and cell number). Samples sizes were sufficiently large to ensure no issues of minimum or maximum limits of detection being reached or loss of linearity).
Data exclusions	No data was excluded.
Replication	A minimum of 3 independent biological repeats with mean +/- SD unless otherwise indicated in the figure legends with similar results obtained between independent biological repeats.
Randomization	Randomization was not relevant for this study as this study did not involve human subjects.
Blinding	Blinding was not relevant to this study as we did not perform any manual group allocation.

Reporting for specific materials, systems and methods

We require information from authors about some types of materials, experimental systems and methods used in many studies. Here, indicate whether each material, system or method listed is relevant to your study. If you are not sure if a list item applies to your research, read the appropriate section before selecting a response.

Materials & experimental systems

n/a	Involved in the study	
[ ]	[x]	Antibodies
[ ]	[x]	Eukaryotic cell lines
[x]	[ ]	Palaeontology and archaeology
[x]	[ ]	Animals and other organisms
[x]	[ ]	Human research participants
[x]	[ ]	Clinical data
[x]	[ ]	Dual use research of concern

Methods

n/a	Involved in the study	
[x]	[ ]	ChIP-seq
[x]	[ ]	Flow cytometry
[x]	[ ]	MRI-based neuroimaging

Antibodies

Antibodies used	This information is provided in the Supplementary Information under 'Immunoblotting', details plus additional clone information as below:-<br>anti-Src (total) (Cell Signalling Technology #2123, clone 32G6, 1:1000), anti-phosphorylated Src (Y527) (Cell Signalling Technology #2105 1:1000), anti-phosphorylated Src (Y416) (Cell Signalling Technology #6943, clone D49G4 1:1000), anti-AMPKα (total) (Cell Signalling Technology #2532 1:1000), anti-phosphorylated AMPKα (T172) (Cell Signalling Technology #2535, 40H9 clone, 1:1000), anti-phospho-Tyr (pan) (Cell Signalling Technology #8954, P-Tyr-1000, 1:2000), anti-p53 (Santa Cruz, DO-1 clone, 1:1000,), anti-β-actin (Merck, MAB1501, clone C4, 1:40,000), anti-phosphorylated LDH-A (Y10) (Cell Signalling Technology #8176 1:1000), anti-LDH-A/B (total) (abcam ab134187 1:5000), anti-AMPKβ1 (total) (Cell Signalling Technology #4150 1:1000), anti-phosphorylated AMPKβ1 (S108) (abcam ab156890 1:500)<br>Anti-rabbit or anti-mouse secondary antibody as appropriate (HRP-conjugated; 1:5000, Dako P0448 (anti-rabbit) or Dako P0260 (anti-mouse)).
Validation	Cell Signalling Technology (CST) antibodies are validated in-house for specificity and cross-species reactivity. CST validation steps for western blotting include:-

-Examination of several cell lines and/or tissues of known expression levels allows accurate determination of species cross-reactivity and verifies specificity.
-Treatment of cell lines with growth factors, chemical activators or inhibitors, which induce or inhibit target expression, verifies specificity.
-Phosphatase treatment confirms phospho-specificity.
-The use of siRNA transfection or knockout cell lines verifies target specificity.
-Side-by-side comparison of lots to ensure lot-to-lot consistency.
-Optimal dilutions and buffers are predetermined, positive and negative cell extracts are specified, and detailed protocols are already optimized, saving valuable time and reagents.

Citations for the indicated antibodies are as follows (citeab):-
Src total (32G6), 110 citations
Y527P Src, 118 citations
Y416P Src, 224 citations
AMPKa, 1157 citations
T172P AMPKa, 1576 citations
phospho-Tyr(pan), 56 citations
p53 (DO1), 4275 citations
Actin (C4), 1807 citations
phosphorylated LDH-A (Y10), 12 citations
LDH-A/B total, 4 citations (validated by RNAi in Allison et al. 2014, Oncogenesis, 3, e102)
AMPKB1 total, 49 citations
phosphorylated S108 AMPKB1, 0 citations on citeab, validated by supplier (abcam)

Other validation details:-
- Src (32G6) Rabbit mAb detects endogenous levels of total Src proteins. The antibody does not cross-react with other proteins. Species Reactivity:Human, Mouse, Rat, Monkey.
-Phospho-Src (Tyr527) Antibody detects endogenous levels of Src only when phosphorylated at Tyr527. The antibody may cross-react with other Src family members such as Yes, Fyn, Fgr and Yrk when phosphorylated at the equivalent sites. Species Reactivity: Human, Mouse, Rat.
-Phospho-Src (Tyr416, clone D49G4) Rabbit mAb detects endogenous levels of Src only when phosphorylated at Tyr416. The antibody may cross-react with other Src family members (Lyn, Fyn, Lck, Yes and Hck) when phosphorylated at equivalent sites. It may cross react with overexpressed phosphorylated RTKs. Species Reactivity: Human, Mouse, Rat, Monkey.
-AMPKalpha Antibody detects endogenous levels of AMPKα protein. The antibody detects both the α1 and α2 isoforms of the catalytic subunit, but it does not detect the regulatory β or γ subunits. Species Reactivity: Human, Mouse, Rat, Hamster, Monkey.
-Phospho-AMPKα (Thr172) (40H9) Rabbit mAb detects endogenous AMPKα only when phosphorylated at threonine 172. The antibody detects both α1 and α2 isoforms of the catalytic subunit, but does not detect the regulatory β or γ subunits. Species Reactivity: Human, Mouse, Rat, Hamster, Monkey, D. melanogaster, S. cerevisiae.
-Phospho-Tyrosine (P-Tyr-1000) MultiMab™ Rabbit mAb mix recognizes a broad range of tyrosine-phosphorylated proteins and peptides. This antibody does not cross-react with proteins or peptides containing phospho-Ser or phospho-Thr residues. Species Reactivity:All Species Expected.
-p53 (DO-1) Mouse mAb recognizes endogenous levels of total p53 protein. Species Reactivity: Human
-AB1501 is a pan-actin antibody that binds to an epitope in a highly conserved region of actin; therefore, this antibody reacts with all six isoforms of vertebrate actin.
- #8176 phospho-Y10 LDH-A, Phospho-LDHA (Tyr10) Antibody recognizes endogenous levels of LDHA protein only when phosphorylated at Tyr10.
- ab134187 antibody - ab134187 can recognize LDH-A, B, and C, immunogen: synthetic peptide corresponding to Human Lactate Dehydrogenase aa 1-100.
- S108P AMPKB1 antibody, immunogen: ynthetic peptide within Human AMPK beta 1 aa 100-200 (phospho S108) conjugated to keyhole limpet haemocyanin. The exact sequence is proprietary.
- AMPKB1 total antibody - AMPKbeta1/2 (57C12) Rabbit mAb detects endogenous levels of both total AMPKβ1 and β2 proteins. The antibody does not cross-react with other related proteins.

Eukaryotic cell lines

Policy information about cell lines

Cell line source(s)	Established cancer cell lines HT29, DLD-1, PSN-1, BxPC-3, MiaPaCa2, A549, H460 were all obtained from ATCC.<br><br>Human ARPE-19 non-cancerous retinal epithelial cell line and human MCF10A non-cancerous breast epithelial cell line were both obtained from ATCC.<br><br>HCT116 p53+/+ and p53-/- isogenic clones were obtained from the originator's laboratory (Professor Bert Vogelstein) (Bunz et al. Science 1998). GBM1 and NP1 cells were obtained from Dr Heiko Wurdak, a co-author. GBM1 cells were established from a primary GBM tumour by Heiko Wurdak and colleagues (Wurdak et al. Cell Stem Cell 2010, Polson et.al., Sci Transl. Med., 2018). Non-cancerous adult brain neural progenitor (NP1) cells were derived from a patient undergoing surgery to treat epilepsy (Wurdak et al. Cell Stem Cell 2010, Polson et.al., Sci Transl. Med., 2018).
Authentication	Cell lines were authenticated by STR profiling by the supplier (ATCC or Prof. Bert Vogelstein). Cells were maintained at low passage in antibiotic-free growth media and with all experiments within 10 passages of authentication from liquid nitrogen stocks. GBM1 and NP1 are cells generated by one of the co-authors and such they have not been authenticated as they are not commercially available cell lines.
Mycoplasma contamination	All cell lines were certified to be mycoplasma negative by the supplier and this has been confirmed in-house for the stocks used. GBM1 and NP1 cells also tested negative for mycoplasma contamination (Polson et.al., Sci Transl. Med., 2018).
Commonly misidentified lines<br>(See ICLAC register)	None of the cell lines are reported as misidentified in the ICLAC register.
